# Multi-Target Anti-Aging and Wound-Healing Potential of Libyan *Thymus vulgaris* and *Thymus capitatus* Essential Oils: Integrated GC–MS, Network Pharmacology, Molecular Docking, and In Vitro Evaluation

**DOI:** 10.3390/plants15203238

**Published:** 2026-10-21

**Authors:** Sara Nasser Suliman, Anwagy Al-Masawri, Madiha A. Hassan, Sara Abouzeid

**Affiliations:** 1 Department of Pharmacognosy, Faculty of Pharmacy, Mansoura University, Mansoura 35516, Egypt; sara.attia8886@yahoo.com (S.N.S.); hassanmadiha00@gmail.com (M.A.H.); 2 Department of Pharmacognosy, Faculty of Pharmacy, University of Derna, Derna, Libya; 3 Department of Botany, Faculty of Science, University of Derna, Derna, Libya; anwagy.almasawri@uod.edu.ly

**Keywords:** *Thymus vulgaris*, *Thymus capitatus*, essential oil, GC–MS, network pharmacology, molecular docking, skin aging, enzyme inhibition, wound healing, chemotype, chemometrics

## Abstract

*Thymus vulgaris* (TV) and *Thymus capitatus* (TC) are traditionally used for skin-related disorders, but their comparative chemical composition and multi-target skin-protective mechanisms remain insufficiently characterized. In this study, essential oils from aerial parts collected in eastern Libya were analyzed by GC–MS and evaluated using network pharmacology, molecular docking, enzyme-inhibition and antioxidant assays, and a human fibroblast scratch-wound/RT-qPCR model. GC–MS identified 39 constituents, accounting for 98.95% and 99.23% of the TV and TC oils, respectively; carvacrol (41.38%) and thymol (20.91%) predominated in TV, whereas thymol (41.06%) and carvacrol (13.02%) predominated in TC. Chemometric comparison with 28 published oils per species (PCA and hierarchical clustering) classified the TC oil as a thymol chemotype, found in only seven of 28 regional *T. capitatus* oils (18 were carvacrol type), and the TV oil as a carvacrol-rich type most similar to Moroccan oils. Network pharmacology identified 1074 core targets linked to 14 skin-associated conditions and prioritised 50 hub genes, including *NFKB1* and *RELA*. Docking of 129 compound–target pairs showed that 84 (65%) achieved docking scores ≤−5.0 kcal/mol. TC showed stronger elastase, tyrosinase, hyaluronidase, and 5-LOX inhibition, whereas TV exhibited stronger COX-2 inhibition and antioxidant activity. At a low-toxicity concentration (1.99 µg/mL), TC was associated with accelerated fibroblast wound closure, which may reflect both migration and proliferation, and with increased *COL1A1*, *ITGA11*, and *HAS2* and decreased *MMP1* mRNA expression. Overall, the two oils displayed complementary multi-target anti-aging, anti-inflammatory, and antioxidant activities in vitro and in silico, with TC additionally suggesting pro-regenerative potential in fibroblasts, supporting their further investigation as topical cosmeceutical candidates.

## 1. Introduction

The skin is the largest organ of the human body and serves as a dynamic protective barrier that maintains hydration, defends against pathogens and ultraviolet (UV) radiation, and preserves internal homeostasis [1]. Its structural integrity and physiological function are primarily governed by extracellular matrix (ECM) components, including collagen, elastin, and hyaluronic acid [2]. In addition, melanin, a pigment synthesized by epidermal melanocytes, plays a central role in skin pigmentation [3]. The turnover and homeostasis of these biomolecules are tightly controlled by enzymatic systems such as collagenase, elastase, hyaluronidase, and tyrosinase. Dysregulation of these enzymes, often driven by oxidative stress and chronic inflammation, leads to ECM degradation, pigment imbalance, and functional impairment, thereby contributing to skin aging and a wide spectrum of dermatological disorders, including wrinkles, impaired wound healing, fibrosis, psoriasis, and atopic dermatitis [4,5].

Natural products, particularly plant-derived essential oils (EOs), represent a valuable source of bioactive compounds with multi-target therapeutic potential [6]. Owing to their complex chemical composition, essential oils can simultaneously modulate oxidative stress, inflammatory pathways [7], and enzyme activities involved in ECM degradation and skin dysfunction [8]. Species of the genus *Thymus* (Lamiaceae), especially *Thymus vulgaris* (TV) and *Thymus capitatus* (TC), are widely recognized in traditional medicine and cosmeceutical applications for their rich content of volatile monoterpenes and phenolic compounds such as thymol and carvacrol [9,10]. These constituents have been associated with antioxidant, anti-inflammatory [11], and enzyme-inhibitory properties, suggesting their potential role in the prevention and management of skin-related disorders [8,12]. However, comprehensive evaluations linking their chemical composition to biological activity and underlying molecular mechanisms remain limited.

In recent years, network pharmacology has emerged as an effective systems-level approach to elucidate the multi-target mechanisms of natural products by integrating chemical, biological, and disease-related data. This strategy enables the identification of key molecular targets and signalling pathways involved in complex diseases, including dermatological conditions. When combined with molecular docking, it provides further insight into possible compound–target interactions at the molecular level and helps prioritise targets for experimental study [13,14].

Therefore, the present study was designed to: (i) characterize the chemical composition of essential oils from *Thymus vulgaris* and *Thymus capitatus* using gas chromatography–mass spectrometry (GC–MS) and establish a comprehensive chemometric comparison—utilizing principal component analysis (PCA) and hierarchical clustering—between the newly analyzed volatile profiles and previously published data of the same species growing under diverse environmental conditions across different countries; (ii) investigate their underlying molecular mechanisms through network pharmacology by integrating compound–target prediction, protein–protein interaction (PPI) analysis, hub gene identification, and pathway enrichment analysis, followed by molecular docking analysis; (iii) evaluate their inhibitory activity against key skin-related enzymes, including elastase, hyaluronidase, collagenase, tyrosinase, and 5-lipoxygenase (5-LOX), as well as their antioxidant activities; and (iv) explore the effects of TC oil on scratch-wound closure and on *COL1A1*, *MMP1*, *ITGA11*, and *HAS2* transcript levels in human skin fibroblasts. By combining experimental and computational approaches, this study provides a comprehensive framework to elucidate the multi-target therapeutic potential of these essential oils in the prevention and management of skin-related disorders.

## 2. Results

### 2.1. Chemical Profiles of the EOs

The qualitative analyses and relative-abundance (peak-area %) data of the main constituents of the EOs extracted from *Thymus vulgaris* and *Thymus capitatus* are presented in Table 1. GC–MS analysis identified 39 constituents (Figures S1 and S2), accounting for 98.95% and 99.23% of the *T. vulgaris* (TV) and *T. capitatus* (TC) oils, respectively. Oxygenated monoterpenes dominated both oils (78.20% in TV; 64.87% in TC), followed by monoterpene hydrocarbons (9.74%; 17.28%). Oxygenated sesquiterpenes were higher in TC (8.99% vs. 2.32%), whereas sesquiterpene hydrocarbons were comparable (8.69% vs. 8.09%). The essential oil yield was 2.70% (*v*/*w*) for TV and 1.22% (*v*/*w*) for TC.

TV oil was carvacrol-rich (41.38 ± 0.30%), followed by thymol (20.91 ± 0.20%), *trans*-caryophyllene (7.15 ± 0.03%), and borneol (7.02 ± 0.04%). TC oil was thymol-rich (41.06 ± 0.30%), followed by carvacrol (13.02 ± 0.10%), γ-terpinene (8.30 ± 0.05%), *p*-cymene (5.21 ± 0.04%), *trans*-caryophyllene (5.10 ± 0.04%), *p*-cumic aldehyde (4.31 ± 0.03%), and caryophyllene oxide (4.10 ± 0.03%). Thus, carvacrol was ~3.2-fold higher in TV and thymol ~2-fold higher in TC, while borneol was markedly higher in TV (7.02% vs. 0.62%) and caryophyllene oxide in TC (4.10% vs. 1.63%).

To place these chemotypes in a regional context, the oil compositions were compared with published data for 28 *T. capitatus* and 28 *T. vulgaris* populations from the Mediterranean region and neighbouring areas using principal component analysis (PCA) and hierarchical clustering, as described in Section 3.1.

### 2.2. Network Pharmacology and Molecular Docking Analysis

#### 2.2.1. Compound–Target Prediction and Core Targets

**Figure 1:**
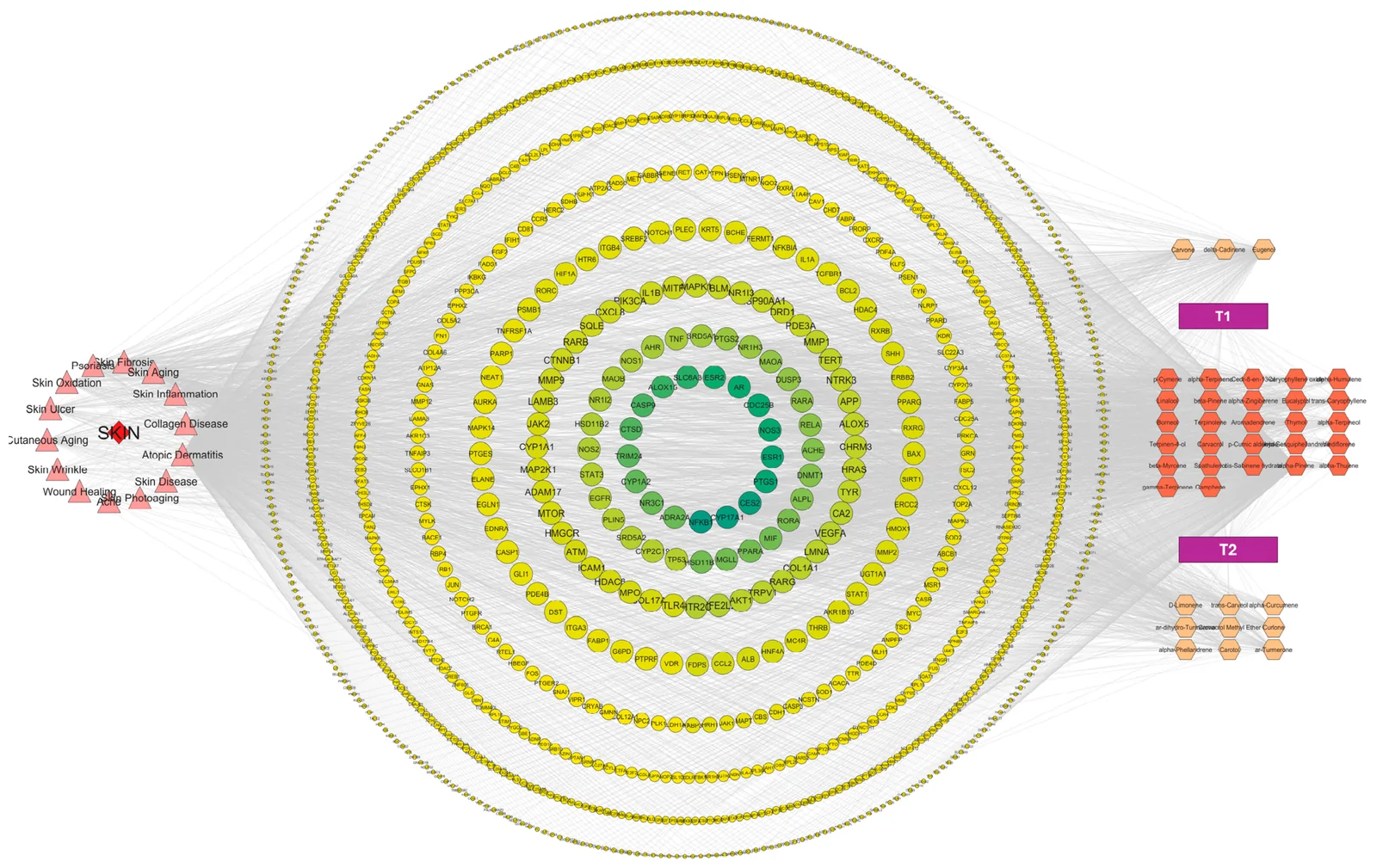
Multi-layer network pharmacology map of *Thymus* essential oils and skin diseases. (**Left**): SKIN hub (red diamond) linked to the 14 disease terms (triangles). (**Centre**): the 1074 core targets (circles), i.e., genes predicted for at least one constituent and associated with at least one skin term after score filtering. (**Right**): the 39 compounds (dark orange = present in both oils, light orange = present in one) and the 2 extracts (purple rectangles; T1 = TV, *T. vulgaris*; T2 = TC, *T. capitatus*).

Target prediction across six platforms (CTD, TargetNet, SwissTargetPrediction, SuperPred, BindingDB, and SEA) yielded 2445 predicted genes for the 39 constituents (25 shared, 3 unique to TV, and 11 unique to TC). Querying seven disease databases (GeneCards, DisGeNET, OpenTargets, OMIM, MalaCards, MyGene.info, and DISEASES) with 14 skin-related terms, followed by score-based filtering, retained 4060 disease-associated genes. The intersection of the 2445 compound-predicted genes with the 4060 disease-associated genes yielded 1074 core targets (Figure S3), which were assembled into an integrative network of 1130 nodes and 4202 edges (including 2439 compound–target and 1685 disease–gene edges) linking extracts, compounds, targets, and disease terms (Figure 1).

#### 2.2.2. PPI Network, Hub Genes, and Compound–Target Sub-Network

**Figure 2:**
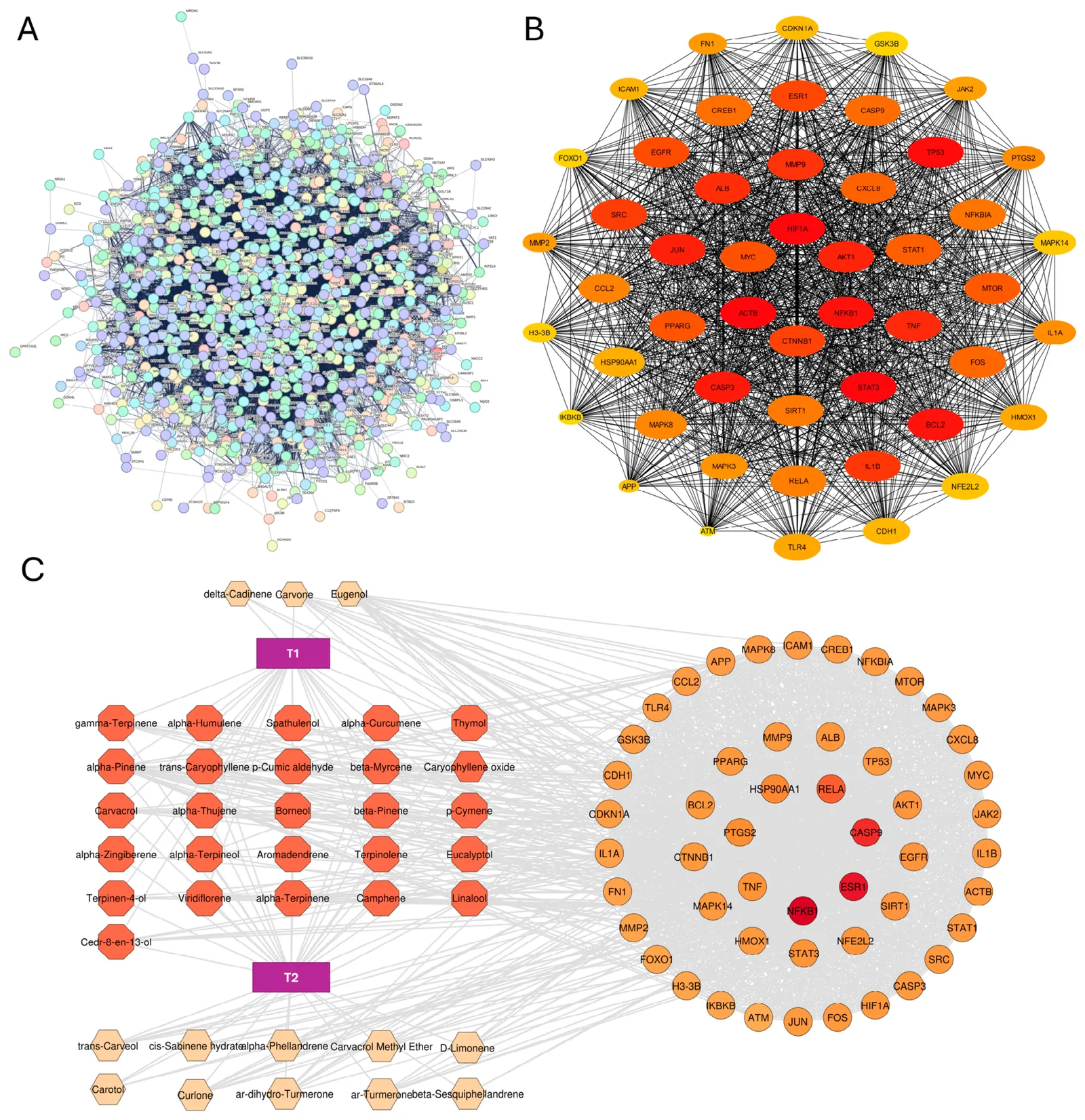
Protein–protein interaction (PPI) and hub network analysis. (**A**) PPI network of the core targets (1068 nodes, 20,418 edges; STRING v12.0, *Homo sapiens*, confidence ≥ 0.400). (**B**) The 50 hub genes identified by Maximal Clique Centrality (MCC) in cytoHubba; node colour runs from red (rank 1) to yellow (rank 50) in the cytoHubba ranking. (**C**) Extract → Compound → Hub Target sub-network (91 nodes, 1485 edges). Purple rectangles: extracts (T1 = TV, *T. vulgaris*; T2 = TC, *T. capitatus*); compounds: dark orange = present in both oils, light orange = present in one; circles: hub targets, coloured by their number of connections in this sub-network (darker = more).

The 1074 core targets formed a STRING PPI network of 1068 nodes and 20,418 edges (Figure 2A). CytoHubba (MCC) prioritised 50 hub genes (Figure 2B; Table S2), the top ten being *AKT1*, *TNF*, *TP53*, *STAT3*, *JUN*, *EGFR*, *MAPK3*, *NFKB1*, *SRC*, and *CASP3*. The extract–compound–hub sub-network (91 nodes, 1485 edges; Figure 2C) showed that eugenol (24 hub genes), α-pinene (22), and linalool (12) had the broadest coverage, while carvacrol and thymol interacted with 7 and 8 hub genes, respectively, including *RELA*, *ESR1*, *JAK2*, *EGFR*, and *PTGS2*.

#### 2.2.3. Functional Enrichment and Pharmacological Flow

**Figure 3:**
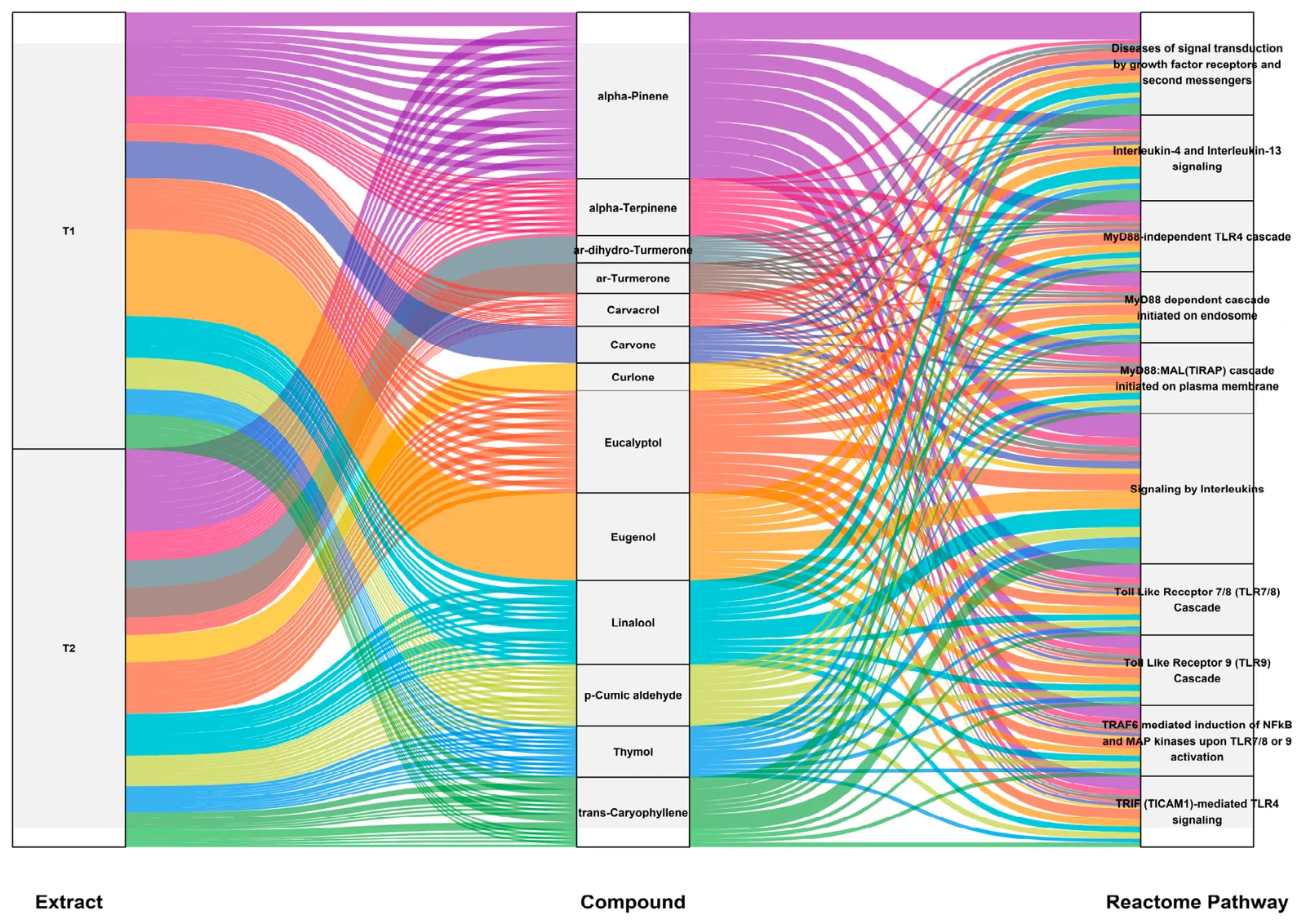
Sankey diagram of pharmacological flow from extracts (T1 = TV, *T. vulgaris*; T2 = TC, *T. capitatus*) through bioactive compounds to enriched Reactome pathways of the 50 hub genes (ReactomePA; Benjamini–Hochberg-adjusted *p* < 0.05). Ribbon width = number of bridging hub genes. Compounds targeting ≥7 hub genes and the 10 most significant pathways are shown.

GO enrichment of the hub genes highlighted regulation of apoptotic signalling, response to oxidative stress, and response to lipopolysaccharide, with localisation to membrane rafts and focal adhesions (Figure S4B). Reactome analysis ranked Signalling by Interleukins first (gene ratio ≈ 0.56; −log10 *p* > 32), followed by IL-4/IL-13 signalling, TRAF6-mediated NF-κB/MAPK induction, and multiple Toll-like receptor and MyD88-dependent cascades (Figure S4A). Apoptotic (*CASP3*, *CASP9*, *BCL2*, *TP53*), inflammatory (*TNF*, *IL1B*, *NFKB1*, *RELA*, *TLR4*), and oxidative-stress (*NFE2L2*, *HMOX1*, *SIRT1*) genes were co-enriched. In the Sankey flow (Figure 3), both extracts reached all major pathways through shared constituents, with eugenol and α-pinene connecting to all top ten pathways.

#### 2.2.4. Molecular Docking Analysis

**Figure 4:**
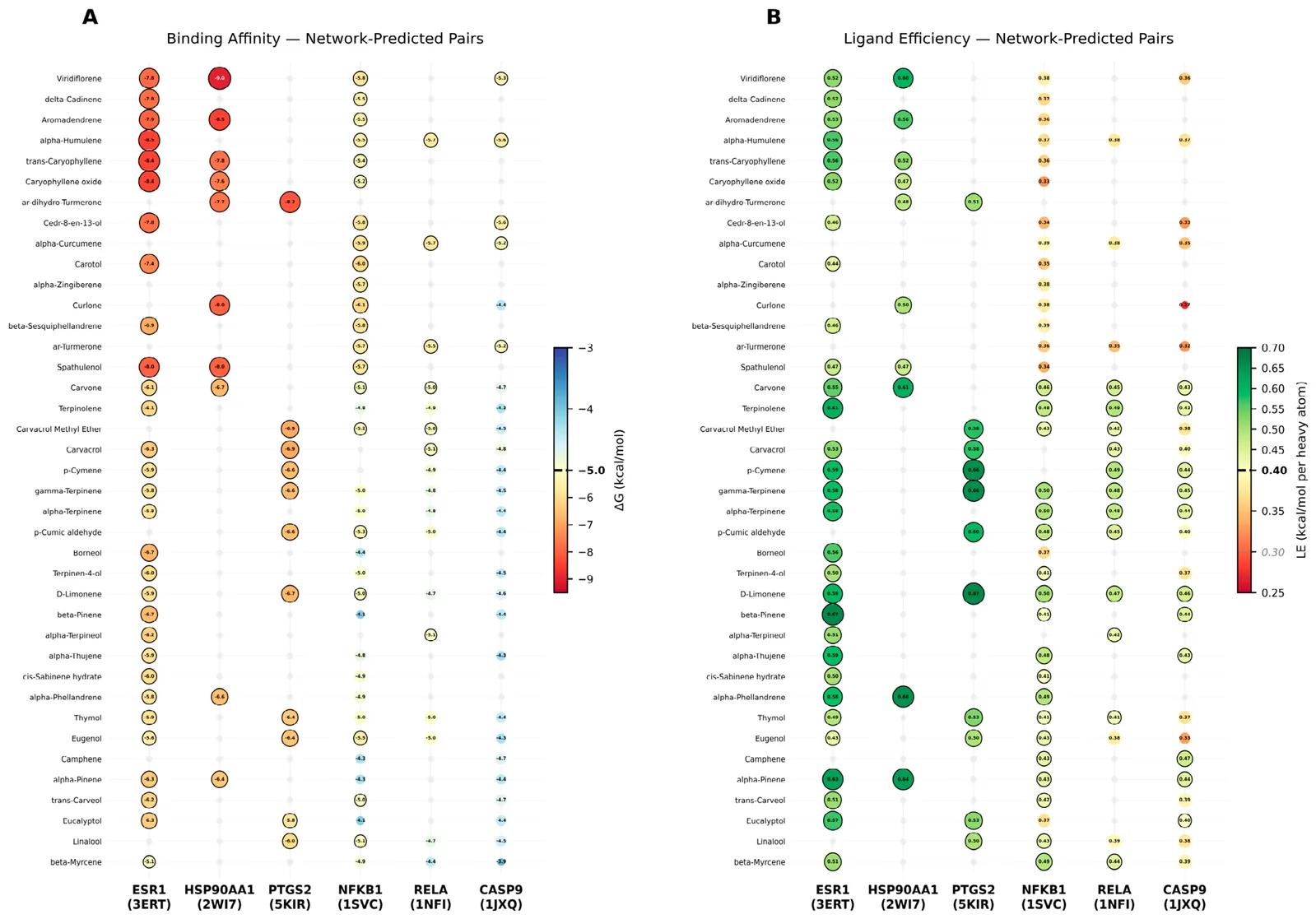
Dual-panel bubble heatmap of molecular docking results for the 129 network-predicted compound–target pairs across the 6 docking targets (hub genes with ≥10 predicted compounds; PDB IDs in parentheses). (**A**) Binding affinity (ΔG, kcal/mol); circle size proportional to |ΔG|; RdYlBu palette centred at −5.0 kcal/mol; black inner rings = ΔG ≤ −5.0 (conventional cut-off). (**B**) Ligand efficiency (LE = −ΔG/N_heavy_); circle size proportional to LE; RdYlGn palette centred at 0.40; black inner rings = LE ≥ 0.40. Grey dots indicate compound–target combinations not predicted by the network. Docking: AutoDock Vina 1.2.7, exhaustiveness 128, seed 42.

Molecular docking was performed for the 129 compound–target pairs predicted by the network pharmacology pipeline across the six compound-convergence hub targets. All runs completed successfully. Binding affinities are summarised in Table S3 and visualised in Figure 4.

Of the 129 network-predicted compound–target pairs, 84 (65%) reached the conventional −5.0 kcal/mol cut-off (Figure 4A), 93 (72%) achieved LE ≥0.4 (excellent), and 128 (99%) met the LE ≥0.3 (good) threshold (Figure 4B). The per-target results are presented below.

*ESR1 (oestrogen receptor α)*

All 30 predicted compounds docked into the ESR1 ligand-binding domain (PDB 3ERT; grid centred on co-crystallised OHT) scored ≤−5.0 kcal/mol (100%), and all achieved LE ≥ 0.4 (mean LE 0.54). α-Humulene (−8.5 kcal/mol, LE 0.56), *trans*-caryophyllene (−8.4, LE 0.56), caryophyllene oxide (−8.4, LE 0.52), and spathulenol (−8.0, LE 0.50) scored highest. The full hit rate likely reflects the broad hydrophobic cavity of the LBD, which readily accommodates the compact terpene scaffolds.

*HSP90AA1 (heat shock protein 90 α)*

All 10 predicted compounds docked at the HSP90AA1 ATP-binding pocket (PDB 2WI7; grid centred on co-crystallised thienopyrimidine inhibitor 2KL) scored ≤−5.0 kcal/mol (100%) and achieved LE ≥0.4 (mean LE 0.55). Viridiflorene scored −9.0 kcal/mol (LE 0.60), followed by aromadendrene (−8.5, LE 0.56), spathulenol (−8.0, LE 0.50), and curlone (−8.0, LE 0.50). The enclosed geometry of the N-terminal ATP pocket likely contributes to both the high raw affinities and the strong ligand efficiencies observed here.

*PTGS2 (COX-2)*

All 11 predicted compounds docked into the COX-2 active channel (PDB 5KIR; grid centred on co-crystallised RCX) scored ≤−5.0 kcal/mol (100%), and all achieved LE ≥0.4 (mean LE 0.57). ar-Dihydro-turmerone had the best docking score (−8.2 kcal/mol, LE 0.51), followed by carvacrol methyl ether (−6.9, LE 0.58) and carvacrol (−6.9, LE 0.63). Among the smaller monoterpenes, D-limonene (−6.7, LE 0.67) and *p*-cymene (−6.6, LE 0.66) achieved the highest ligand efficiencies, reflecting efficient per-atom engagement despite lower absolute affinities.

*NFKB1 (NF-κB p50)*

NFKB1 was docked using a grid centred on the DNA-contact surface (34 residues within 4.5 Å of DNA in 1SVC; box 38 × 37 × 40 Å). Of the 35 predicted compounds, 21 (60%) reached −5.0 kcal/mol, with curlone (−6.1, LE 0.38), carotol (−6.0, LE 0.35), α-curcumene (−5.9, LE 0.39), and β-sesquiphellandrene (−5.8, LE 0.39) scoring highest (Figure 4A). By LE, 19/35 (54%) exceeded 0.4 (mean LE 0.41; Figure 4B), suggesting that the moderate raw scores largely reflect the flat, solvent-exposed binding surface rather than poor compound quality.

*RELA (NF-κB p65)*

RELA lacks a conventional small-molecule pocket; the grid was focused on the DNA-binding surface of the Rel homology domain (PDB 1NFI; 23 DNA-contact residues mapped from 1VKX; box 40 × 45 × 75 Å). Of the 17 predicted compounds, 7 (41%) reached −5.0 kcal/mol: α-curcumene (−5.7, LE 0.38), α-humulene (−5.7, LE 0.38), and ar-turmerone (−5.5, LE 0.35) scored highest. By LE, 12/17 (71%) achieved ≥0.4 (mean LE 0.43), suggesting that per-atom binding at this flat surface is comparable to that at the enclosed-pocket targets. The lower absolute ΔG values are therefore likely to reflect the binding-site geometry as well as weaker interactions.

*CASP9 (caspase-9)*

Of the 26 predicted compounds docked at the CASP9 catalytic site (PDB 1JXQ; grid centred on co-crystallised PHQ), 5 (19%) scored ≤−5.0 kcal/mol: α-humulene (−5.6, LE 0.37), cedr-8-en-13-ol (−5.6, LE 0.35), viridiflorene (−5.3, LE 0.36), α-curcumene (−5.2, LE 0.35), and ar-turmerone (−5.2, LE 0.32). By LE, 11/26 (42%) achieved ≥0.4 (mean LE 0.39). The low raw ΔG scores are expected for the shallow peptide-substrate groove of caspase-9; the moderate LE values suggest that per-atom binding efficiency remains reasonable.

#### 2.2.5. Binding Mode Visualization of Top Compound–Target Complexes

**Figure 5:**
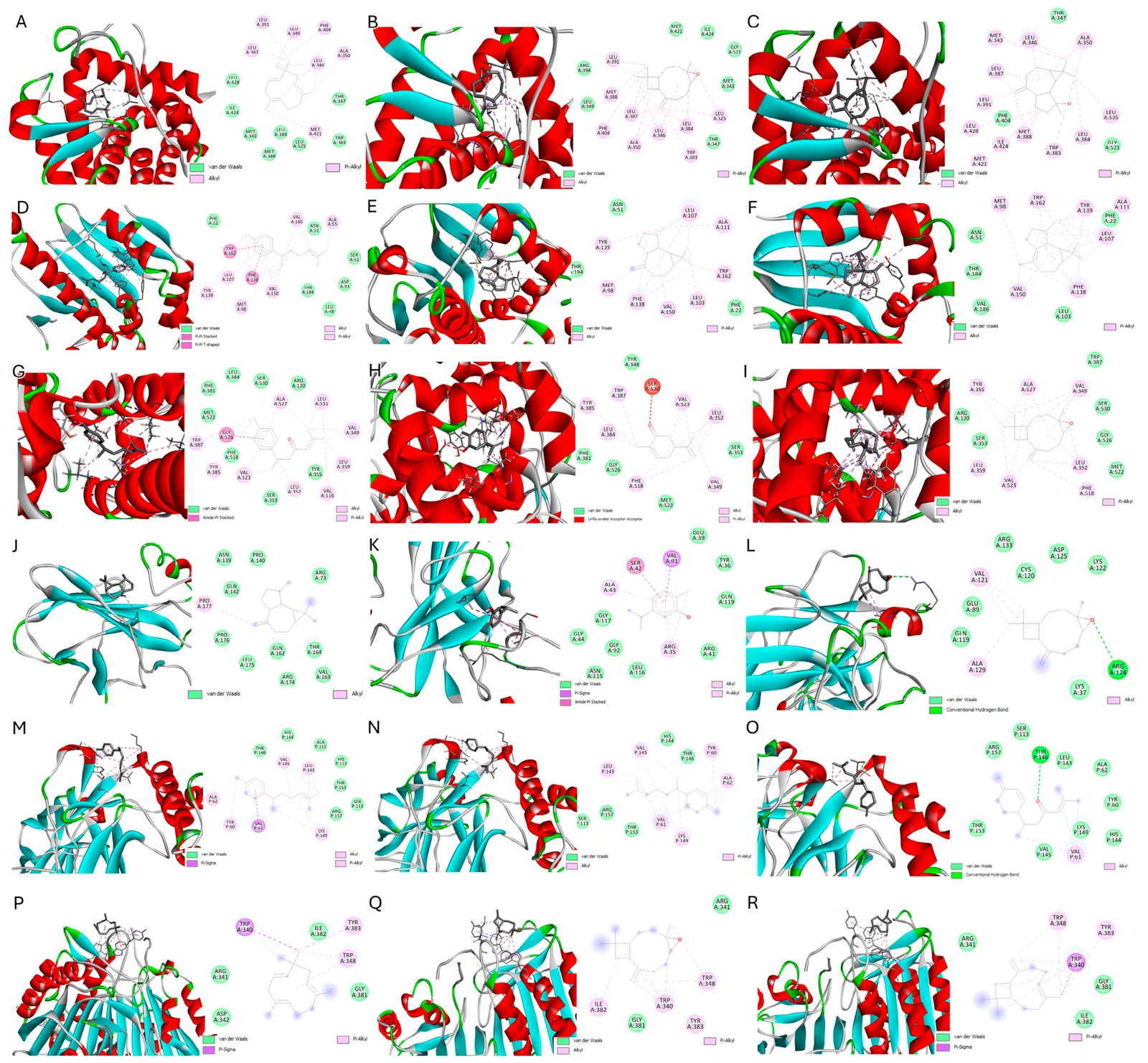
Three-dimensional docking poses (**left**) and two-dimensional interaction diagrams (**right**) for representative compound–target complexes at each hub target. (**A**–**C**) ESR1: α-humulene, caryophyllene oxide, spathulenol. (**D**–**F**) HSP90AA1: α-curcumene, aromadendrene, viridiflorene. (**G**–**I**) PTGS2: ar-dihydro-turmerone, carvone, caryophyllene oxide. (**J**–**L**) NFKB1: α-curcumene, β-sesquiphellandrene, curlone. (**M**–**O**) RELA: aromadendrene, carvacrol, caryophyllene oxide. (**P**–**R**) CASP9: α-humulene, caryophyllene oxide, trans-caryophyllene. Interaction types: green = van der Waals; pink = alkyl/Pi-alkyl; purple = Pi-sigma; dark green dashed = conventional hydrogen bond; red = unfavourable acceptor–acceptor. Visualized with BIOVIA Discovery Studio Visualizer 2021 (v21.1.0; Dassault Systèmes, San Diego, CA, USA).

To examine the binding modes of the top-scoring hits, three-dimensional poses and two-dimensional interaction diagrams were generated for each hub target using BIOVIA Discovery Studio Visualizer (Figure 5; Table S3). Three compounds per target were selected on the basis of binding affinity and scaffold diversity, giving 18 panels (A-R). The 3D views (left) show the ligand inside the binding site in ribbon representation.

At ESR1 (panels A–C), α-humulene (−8.5 kcal/mol), caryophyllene oxide (−8.4), and spathulenol (−8.0) occupied the ligand-binding domain with extensive hydrophobic contacts to LEU346, LEU387, MET388, LEU391, LEU404, and MET421, consistent with the apolar character of the tamoxifen-binding pocket. At HSP90AA1 (panels D–F), α-curcumene (−8.1 kcal/mol), aromadendrene (−8.5), and viridiflorene (−9.0) filled the ADP/inhibitor pocket through deep van der Waals engagement of ASN51, ASP93, LEU107, PHE138, and VAL186. At PTGS2 (panels G–I), ar-dihydro-turmerone (−8.2), carvone (−7.2), and caryophyllene oxide (−7.4) docked into the cyclooxygenase channel, forming alkyl and Pi-alkyl contacts with TRP387, TYR385, ALA527, VAL349, LEU352, and SER353. At NFKB1 (panels J–L), α-curcumene (−5.9), β-sesquiphellandrene (−5.8), and curlone (−6.1) engaged the solvent-exposed surface of the p50 subunit through van der Waals interactions with PRO177, GLN177, LEU162, and ARG71, alongside conventional hydrogen bonds at GLU89 and GLN119 for α-curcumene. At RELA (panels M–O), aromadendrene (−5.9 kcal/mol), carvacrol (−5.1), and caryophyllene oxide (−5.8) contacted the p65 subunit surface via van der Waals and Pi-sigma interactions at TYR46, HIS144, THR145, and ALA146. At CASP9 (panels P–R), α-humulene (−5.6), caryophyllene oxide (−5.5), and *trans*-caryophyllene (−5.6) docked near the catalytic groove with Pi-sigma and van der Waals contacts to TRP348, ARG341, ILE382, TYR383, and GLY381.

### 2.3. In Vitro Enzyme Inhibition Assays

The inhibitory potential of *Thymus vulgaris* (TV) and *Thymus capitatus* (TC) essential oils (EOs) was evaluated against a panel of enzymes involved in skin aging and inflammation, including collagenase, elastase, tyrosinase, hyaluronidase, 5-lipoxygenase (5-LOX), and cyclooxygenase-2 (COX2). The results, expressed as IC50 values (µg/mL), are summarized in Table 2.

#### 2.3.1. Inhibition of Skin-Aging Related Enzymes

Regarding anti-elastase activity, TC EO demonstrated superior potency (42.96 ± 1.14) compared to both TV EO (76.23 ± 2.03) and the reference standard, Daidzein (52.44 ± 1.4). Similarly, TC EO exhibited a high inhibitory effect on tyrosinase (15.4 ± 0.43), which was notably more potent than the standard Kojic Acid (18.65 ± 0.52). In the anti-hyaluronidase assay, TC EO (20.82 ± 0.64) showed approximately 2.5 times higher activity than TV EO (53.05 ± 1.63), approaching the efficacy of Luteolin (17.81 ± 0.55). Conversely, both oils showed comparable and moderate anti-collagenase activities (70.53 ± 2.22), (71.79 ± 2.29), slightly lower than the reference Piroxicam (63.06 ± 2.01).

#### 2.3.2. Anti-Inflammatory Enzyme Inhibition

The EOs displayed in vitro anti-inflammatory activity through inhibition of 5-LOX and COX2. TC EO was particularly effective against 5-LOX (0.986 ± 0.03), showing higher potency than TV EO (1.733 ± 0.05), although both were less potent than the standard Zileuton (0.62 ± 0.02). Interestingly, TV EO emerged as a highly potent inhibitor of COX2 (2.934 ± 0.085), outperforming both TC EO (9.397 ± 0.271) and the reference drug Meloxicam (6.038 ± 0.174), suggesting potential for modulating inflammatory pathways.

### 2.4. Antioxidant Activities

The antioxidant capacity of *Thymus vulgaris* (TV) and *Thymus capitatus* (TC) essential oils was evaluated using two complementary assays: the DPPH radical scavenging assay and the Ferric Reducing Antioxidant Power (FRAP) assay. The results are presented in Table 3.

#### 2.4.1. DPPH Radical Scavenging Activity

The free radical scavenging potential was determined by the concentration required to inhibit 50% of DPPH radicals (IC_50_). Both essential oils exhibited notable scavenging activity; however, TV EO proved to be more potent with an IC_50_ value of (29.71 ± 0.935), compared to (36.99 ± 1.165) for TC EO. While both oils showed significant antioxidant effects, their potency remained lower than that of the reference standard, Ascorbic acid (18.75 ± 0.59).

#### 2.4.2. Ferric Reducing Antioxidant Power (FRAP)

The results of the FRAP assay, which measures the ability of the oils to reduce ferric ions (Fe^3+^) to ferrous ions (Fe^2+^), were consistent with the DPPH findings. TV EO displayed a higher reducing capacity (0.029 ± 0.001) than TC EO (0.021 ± 0.0007). These data suggest that the essential oil of *Thymus vulgaris* possesses a superior overall antioxidant profile compared to *Thymus capitatus* in both scavenging and reducing mechanisms.

*T. capitatus* was prioritised for functional and transcriptional evaluation of regenerative activity based on converging evidence: (i) in this study, TC oil showed superior inhibition of ECM-remodelling and skin-ageing-related enzymes (elastase, tyrosinase, hyaluronidase, 5-LOX) relative to TV oil (Table 2); (ii) *T. capitatus* infusion preparation has shown wound-healing activity in earlier studies, including accelerated fibroblast migration in a scratch-wound assay [15]; and (iii) the thymol-dominant chemotype of TC oil (41.06%) aligns with a broader wound-healing literature indicating that thymol and carvacrol act across all three phases of tissue repair, including fibroblast proliferation and collagen deposition [16].

### 2.5. Cytotoxicity, Wound Healing, and Gene Expression Effects of Thymus capitatus Essential Oil

#### 2.5.1. Optimization of *Thymus capitatus* (TC) Essential Oil Concentration

TC essential oil exhibited a significant, dose-dependent inhibitory effect on cell viability. The IC_50_ value for TC essential oil was determined to be 7.97 µg/mL. This low IC_50_ value indicates potent cytotoxic activity against the HSF cell line under the tested condition. As summarized in Table S4 and illustrated in the dose-response curve (Figure S5), cell viability remained high at low concentrations, recorded at 89.00% and 79.06% for 0.1 and 1 µg/mL, respectively. A significant decline in viability (53.68%) was observed at a concentration of 10 µg/mL, while concentrations of 100 µg/mL and above resulted in near-complete cellular inhibition (viability < 0.3%).

To limit the influence of cytotoxicity on the wound-closure readout, a low working concentration was selected. Based on preliminary toxicity assessments, a concentration of 1.99 µg/mL was selected for the wound healing assay. This concentration corresponds to 1/4 of the IC_50_ value, representing a low-toxicity dose that allows evaluation of the oil’s effect on wound closure in human skin fibroblast (HSF) cells.

#### 2.5.2. Effect of TC Essential Oil on HSF Wound Closure

**Figure 6:**
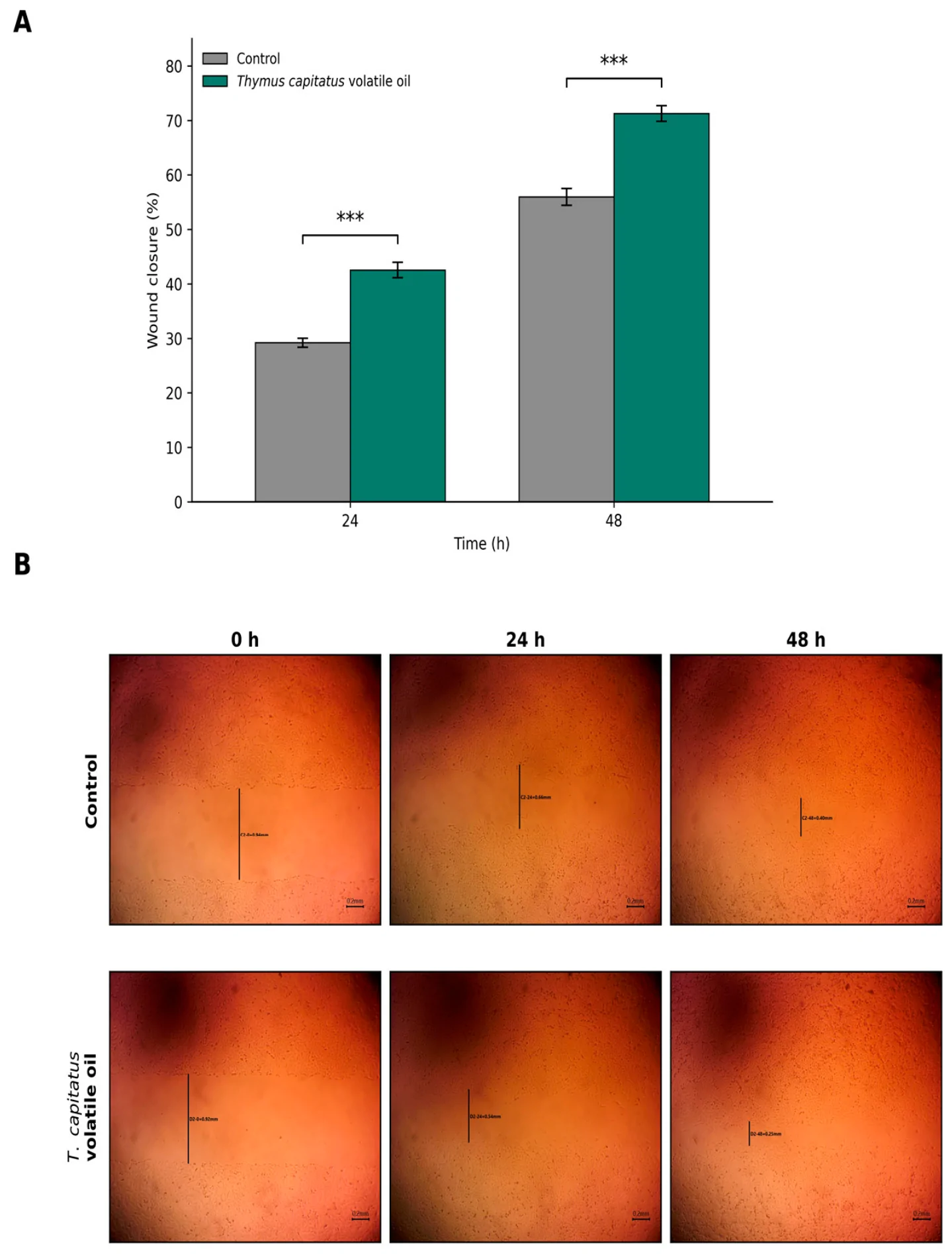
Effect of *Thymus capitatus* volatile oil on wound closure in a scratch assay. (**A**) Wound closure (%) of control and treated cells at 24 and 48 h, calculated as (W_0_ − W_t_)/W_0_ × 100. Data are mean ± SD (*n* = 3); *** *p* < 0.001 versus control (unpaired Student’s *t*-test). (**B**) Representative microscopy images of control and treated cells at 0, 24 and 48 h. Black lines mark wound width (mm); scale bar = 0.2 mm.

The regenerative capacity of the *T. capitatus* (TC) essential oil was evaluated using a standardized scratch-wound healing assay. The results showed that treatment with TC essential oil significantly accelerated wound closure compared to the untreated control group over the 48-h observation period, as visualized in the representative micrographs (Figure 6). The initial wound widths for both the control and treated groups were comparable (0.94 mm vs. 0.91 mm), establishing a consistent baseline. However, by the 24-h mark, the group treated with the TC essential oil exhibited a more pronounced reduction in wound width, reaching 0.53 ± 0.01 mm, whereas the control group remained at 0.67 ± 0.02 mm. This trend of accelerated wound closure continued at 48 h, with the treated wound narrowing to 0.26 ± 0.01 mm, compared to 0.42 ± 0.02 mm in the control group. Expressed as percentage closure (Figure 6A) (calculated from the mean wound widths), this corresponded to approximately 42% vs. 29% at 24 h and 71% vs. 55% at 48 h for treated vs. control cells. These findings suggest that TC essential oil may enhance fibroblast-mediated wound closure in vitro; however, further studies using proliferation-controlled migration assays or chemotaxis models are required to determine whether the effect is mediated by directional fibroblast recruitment.

#### 2.5.3. Impact of TC Essential Oil on the Gene Expression Profile of Human Skin Fibroblasts (HSFs)

To evaluate the potential of *T. capitatus* essential oil in modulating skin health and anti-aging markers, the mRNA expression levels of key genes (*COL1A1*, *MMP1*, *ITGA11*, and *HAS2*) were quantified in Human Skin Fibroblasts (HSF) using RT-qPCR. Gene expression was normalized to *GAPDH* as an internal control, and relative fold changes were calculated using the 2^−ΔΔ*Ct*^ method with the results summarized in Table 4.

Treatment with TC essential oil induced significant transcriptional modifications across all studied markers compared to the untreated control group. COL1A1 mRNA expression showed a 10.14-fold increase, suggesting increased COL1A1 transcription. In parallel, TC essential oil showed marked downregulation of MMP1 mRNA (matrix metalloproteinase-1), with a downregulation to 0.34-fold relative to the control. This concurrent increase in collagen expression and decrease in its primary degrading enzyme is consistent with a transcriptional profile favouring extracellular matrix (ECM) preservation, although protein-level and functional confirmation are required.

Furthermore, TC essential oil treatment influenced cellular adhesion and hydration pathways. The expression of *ITGA11* (Integrin alpha 11) was upregulated by 2.94-fold, potentially reflecting enhanced fibroblast-matrix interactions. Notably, a substantial elevation in *HAS2* (Hyaluronan synthase 2) expression was recorded (7.55-fold), suggesting potentially increased hyaluronan synthesis, which is essential for skin hydration and structural integrity. These data are limited to mRNA expression; mRNA changes do not necessarily translate into protein abundance or functional ECM remodelling [17].

## 3. Discussion

This study linked the chemotype of two eastern Libyan *Thymus* oils to their predicted and measured skin-related activities. The oils were chemically distinct (a carvacrol-rich *T. vulgaris* (TV) and an uncommon thymol-rich *T. capitatus* (TC)), and their activities were complementary: TV was the stronger COX-2 inhibitor and antioxidant, whereas TC was the stronger inhibitor of elastase, tyrosinase, hyaluronidase, and 5-LOX and, at a low-toxicity concentration, accelerated fibroblast wound closure and shifted ECM-related transcripts. We first place the two chemotypes in a regional context, then relate them to the network-pharmacology and in vitro findings, and finally consider safety, limitations, and next steps.

### 3.1. Chemotypes of the Libyan Oils in a Regional Context

GC–MS profiling revealed two phenolic chemotypes: a carvacrol-rich TV oil (carvacrol 41.38%, thymol 20.91%) and a thymol-rich TC oil (thymol 41.06%, carvacrol 13.02%), with oxygenated monoterpenes predominating in both (78.20% and 64.87%), as is typical of thyme oils [9,10]. TC was further distinguished by higher γ-terpinene, p-cymene and total monoterpene hydrocarbons (17.28% vs. 9.74%) and by caryophyllene oxide (4.10% vs. 1.63%), whereas TV was enriched in borneol (7.02% vs. 0.62%).

The TC oil is unusual when compared with published data (Table S8a). Most Mediterranean *T. capitatus* (syn. *Thymbra capitata*) oils are carvacrol-rich: 14 of 28 compiled oils contained 56–82% carvacrol, including samples from Tunisia [18], Morocco, Italy, Spain, Turkey, and north-western Libya [19,20], and earlier eastern Libyan samples were also carvacrol-dominated (thymol ≤ 1.8%) [10,21]. Thymol-dominated oils are less common (7 of 28, including ours) and have been reported mainly from Tunisia (70–89% thymol) [22,23], Algeria [24], Palestine [25], and Lemnos [26]. Mixed carvacrol/thymol profiles have also been reported, for example, in Egypt [27]. Our oil resembles the Algerian, Hebron, and Greek oils and is the only eastern Libyan sample in the dataset with thymol as the main constituent. Its lower total phenol content (54.1%) reflects the still abundant precursors γ-terpinene and p-cymene (13.5% combined).

*T. vulgaris* is similarly polymorphic. Thymol-dominated oils were the most frequent in the dataset (15 of 28), including Italian samples [28], whereas carvacrol-dominated oils (7 of 28, including ours) came from Tunisia [29], Greece [30], Turkey [31], and Morocco [32], and non-phenolic chemotypes also occur [33,34] (Table S8b). The Libyan TV oil is most similar to the Moroccan Taounate and Tiznit oils [35,36], although its γ-terpinene content is much lower (3.50% vs. 19–24%), and its borneol content (7.02%) exceeds that of every other thymol- or carvacrol-type oil in the dataset (≤3.8%).

**Figure 7:**
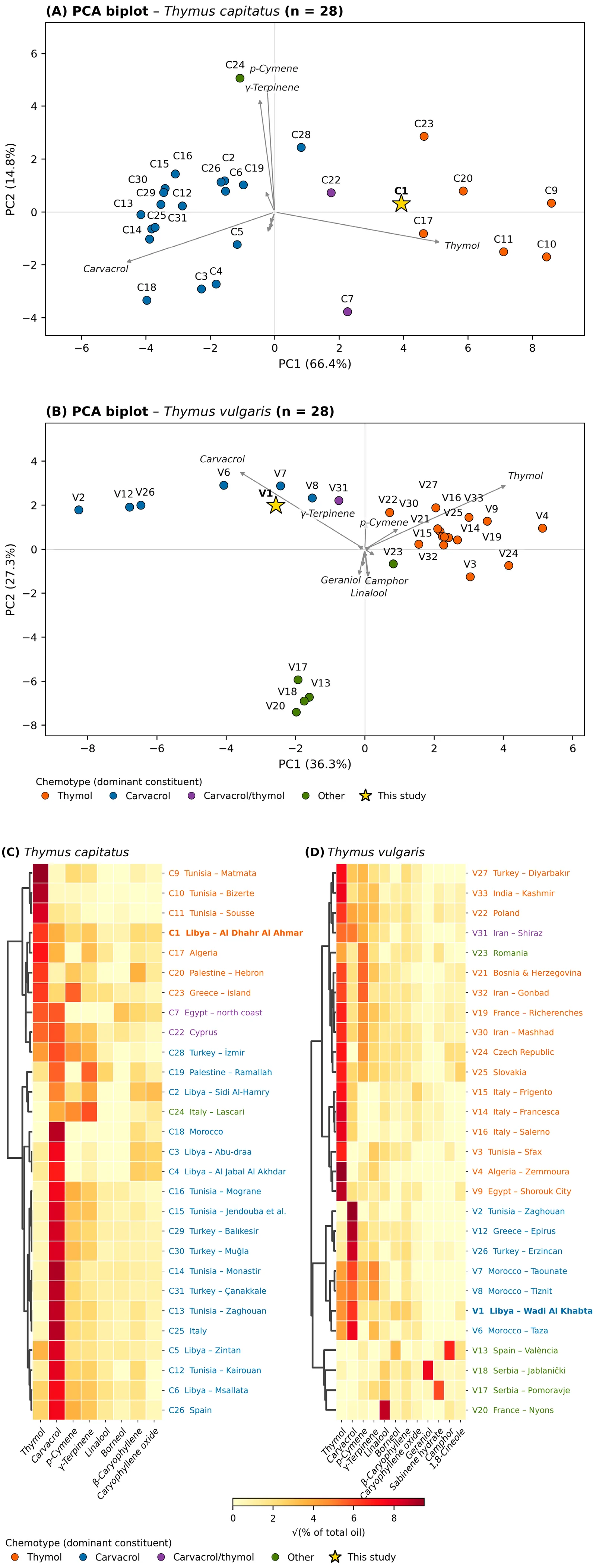
Chemometric comparison of the essential oils from this study with published oils (28 populations per species; square-root-transformed percentages). (**A**,**B**) Principal component analysis (PCA; mean-centred data) of (**A**) *T. capitatus* and (**B**) *T. vulgaris*; arrows show variable loadings. (**C**,**D**) Heatmaps of the same data for (**C**) *T. capitatus* and (**D**) *T. vulgaris*, with sources ordered by Ward hierarchical clustering. Points and labels are coloured by chemotype (dominant constituent; carvacrol/thymol = both phenols ≥ 25% and within 1.5-fold); the star and bold labels mark this study. Codes are as in Tables S8a,b, S9 and S10. (C = *T. capitatus*, V = *T. vulgaris*).

PCA and Ward clustering (Figure 7) supported these assignments. For *T. capitatus*, PC1 (66.4% of the variance) separated thymol- from carvacrol-dominated oils; the eastern Libyan oil (C1) grouped with the Algerian, Hebron, Cypriot, and Greek oils, apart from the near-pure thymol Tunisian oils (C9–C11), while all earlier Libyan samples (C2–C6) lay on the carvacrol side. For *T. vulgaris*, the Libyan oil (V1) clustered with the carvacrol-rich Moroccan oils (V6–V8) and stood out within that group by its elevated borneol and β-caryophyllene. The Libyan TC is thus a thymol type and the Libyan TV a carvacrol type, the reverse of the pattern most often reported for each species (Tables S9 and S10).

The dominant phenol appears to be genetically fixed, whereas environmental factors probably modulate the precursor and sesquiterpene fractions. Thymol- and carvacrol-type oils were reported from similar seasons and regions, for example in Tunisia [18,22,23] and Palestine [25], and altitude [10], harvest time [37] and wild-population chemodiversity [34] all influence oil composition. The higher levels of γ-terpinene and *p*-cymene in TC may reflect differences in the activity of the enzymes that convert these precursors to thymol and carvacrol through cytochrome P450-dependent hydroxylation [38]. Because our TC and TV samples each came from one site and one season (Section 4.1), the results describe these specimens and not the species as a whole.

### 3.2. Linking Chemistry to Predicted Targets and In Vitro Activity

Thymol and carvacrol are positional isomers with overlapping antimicrobial, antioxidant, anti-inflammatory, and wound-healing properties [11,16], and borneol is also anti-inflammatory [39]; direct comparisons are limited, so minor fractions may account for part of the difference between the oils. The chemotypic contrast therefore anticipates the complementary activity profiles observed here. The predicted hub targets map onto established axes of skin pathology and were partly supported by the in vitro results, which are discussed below along an inflammatory/oxidative axis (mainly TV) and an ECM-remodelling/regeneration axis (mainly TC). The assays measured whole-oil activity rather than direct target engagement, although several assayed endpoints (*PTGS2*, *ALOX5*, *MMP1*, and *COL1A1*) are among the predicted core targets.

*Inflammatory and oxidative axis.* TNF and the NF-κB subunits *NFKB1/RELA* (core hub genes and docking targets) drive the inflammatory loop underlying psoriasis [40] and its downstream mediators *IL1B*, *CXCL8*, and *MMP9* [41], while the top-ranked Reactome term IL-4/IL-13 signalling reflects barrier dysfunction in atopic dermatitis [42] and the enriched Toll-like receptor/MyD88 cascades implicate innate immune recognition [43]. Accordingly, both oils strongly inhibited 5-LOX (*ALOX5*, a predicted core target; TC 0.986 vs. TV 1.733 µg/mL; zileuton 0.62 µg/mL), consistent with reports on thymol- and carvacrol-rich *Thymus* oils [12]. *PTGS2* (COX-2), which is required for wound repair but detrimental when chronically elevated [44], had the highest mean ligand efficiency (0.57), and TV, whose dominant constituent carvacrol docked efficiently at this target (ΔG −6.9 kcal/mol, LE 0.63), was the more potent COX-2 inhibitor (IC_50_ 2.934 vs. 9.397 µg/mL for TC; meloxicam 6.038 µg/mL). The same oxidative-stress axis (*NFE2L2*, *HMOX1*) [44,45] tracked with TV’s superior DPPH/FRAP performance (IC_50_ 29.71 vs. 36.99 µg/mL; FRAP 0.029 vs. 0.021 mmol AAE/g), in keeping with the known radical-scavenging and anti-inflammatory activity of carvacrol [11].

*ECM-remodelling and regeneration axis.* The thymol-dominant TC oil was the stronger inhibitor of elastase (IC_50_ 42.96 vs. 76.23 µg/mL), tyrosinase, and hyaluronidase (20.82 vs. 53.05 µg/mL), consistent with earlier reports on *T. capitatus* oil [46]. Its predicted attenuation of NF-κB/AP-1(JUN)-linked ECM remodelling was consistent with the downregulation of MMP1 mRNA (0.34-fold), and ESR1, to which all 30 associated constituents docked with ΔG ≤−5.0 kcal/mol and which offers a receptor-level route to collagen homeostasis [47], was consistent with the upregulation of COL1A1 mRNA (10.14-fold), ITGA11 (2.94-fold; the principal collagen-binding integrin of dermal fibroblasts [48]) and HAS2 (7.55-fold). At a sub-cytotoxic concentration (¼ × IC_50_, 1.99 µg/mL), TC oil also accelerated fibroblast wound closure (wound width 0.53 vs. 0.67 mm at 24 h; 0.26 vs. 0.42 mm at 48 h), in line with the predicted proliferation/migration hubs *AKT1*, *EGFR*, MAPK3, STAT3, and SRC and with regenerative effects reported for other essential-oil phytocomplexes [49]. HSP90AA1, which stabilises NF-κB and JAK–STAT client proteins [50], and the eugenol/α-pinene-mediated inhibition of NF-κB p50–p65 activation [51,52] further support a multi-target mechanism consistent with the traditional dermatological use of *Thymus* oils.

*Chemotype and activity.* The complementary profiles of TV (COX-2 inhibition, antioxidant capacity) and TC (elastase and hyaluronidase inhibition, regenerative activity) parallel their chemistry: TC contained more γ-terpinene, p-cymene, and caryophyllene oxide, and TV contained more carvacrol and borneol (Table 1). A possible, untested interpretation is that carvacrol-rich oils favour control of early inflammation, whereas thymol-type oils with more sesquiterpene oxides favour proliferative and remodelling events, and blends of the two oils could be explored. This cannot be extrapolated to other chemotypes, because wound closure was assessed only for TC, which on the chemometric map, lies with the moderately phenolic thymol oils (Algeria, Hebron, Lemnos) and well away from the near-pure thymol Tunisian oils (C9–C11).

### 3.3. Safety, Limitations and Perspectives

The safety of *Thymus* essential oils depends on their chemical composition and concentration [6,49]. Phenol-rich chemotypes may offer stronger antimicrobial activity [46] but also pose greater risks of irritation and cytotoxicity [16,19], whereas linalool- and geraniol-rich oils may present sensitisation risks associated with oxidation products [53]. In our study, *T. capitatus* (TC) oil exhibited an IC_50_ of 7.97 µg/mL in fibroblasts, with viability decreasing to 79% at 1 µg/mL (Table S4), highlighting the need to establish a clear safety margin for topical application. Both oils also contained minor amounts of recognised fragrance allergens [53]. Therefore, chemotype standardisation and further irritation, sensitisation, and cytotoxicity testing are essential before topical use. Additionally, compound identification relied on NIST/Wiley library matching and Kovats retention indices without authentic standards; future studies should confirm major constituents using reference standards and validated analytical methods.

Molecular docking provides preliminary predictions rather than evidence of direct target engagement. Although the constituents showed favourable scores against ESR1, HSP90AA1, and PTGS2, these results do not establish target specificity or biological inhibition; predictions involving the NFKB1 and RELA protein–DNA interfaces require particular caution. Moreover, the best-scoring minor constituents (<1% of the oils) were estimated to occur at concentrations below 0.1 µM in the fibroblast assays, compared with approximately 5.4 µM for thymol, limiting conclusions about their biological contributions. Protein-level validation, target-engagement assays, and molecular dynamics studies are therefore warranted. Finally, the volatility, hydrophobicity, and oxidation susceptibility of essential oils may compromise stability and skin delivery. Nano-based formulations and cyclodextrin complexes could improve stability and controlled release, but require validation through formulation, skin-permeation, safety, and preclinical efficacy studies before clinical translation [54].

## 4. Materials and Methods

### 4.1. Plant Material

The aerial parts of *T. capitatus* and *T. vulgaris* were collected at the flowering stage during March 2025 from Al Dhahr Al Ahmar and Wadi Al Khabta, eastern Libya, and authenticated at the Botany Department, Faculty of Science, Derna University, according to the Libyan Flora.

### 4.2. Isolation of the Essential Oil

Plants collected were air-dried in the shade at room temperature then slightly ground before extraction. Essential oils were extracted using the traditional water distillation method. A total of 211 g of *T. vulgaris* and 327 g of *T. capitatus* were subjected to hydrodistillation for 3 h using a Clevenger-type apparatus, according to the method described by Clevenger [55]. The obtained essential oils were dehydrated using anhydrous sodium sulfate and stored in sealed dark vials, at low temperature. The extraction procedure was carried out in triplicate to ensure reproducibility [56,57].

### 4.3. GC–MS Analysis

Analysis of EOs was carried out at the Department of Medicinal and Aromatic Plants Research, National Research Center, Egypt, according to the reported protocols [58], under the same techniques and conditions previously reported [59,60]. A TRACE GC Ultra Gas Chromatograph coupled with a Thermo ISQ single quadrupole mass spectrometer detector (Thermo Scientific Corporate, Waltham, MA, USA) was used. The GC/MS system was equipped with a TG-5 MS column (30 m × 0.25 mm inner diameter, 0.25 μm film thickness; Thermo Scientific Corp.). Helium was used as the carrier gas at a flow rate of 1.0 mL/min with a split ratio of 1:10. The column temperature was initially held at 60 °C, then increased at a rate of 4.0 °C/min to 240 °C and held for 1 min. The temperatures of the injector and detector were maintained at 210 °C. Each sample was injected into the injector in a small volume (1 μL in n-hexane) at a concentration of 1:10 (*v*/*v*). To ensure the reproducibility and reliability of the obtained results, each sample was injected twice under the same operating conditions. The mass spectral data were collected at 70 eV by electron ionization with a scan range of *m*/*z* 40–450.

The retention indices were determined for n-alkanes (C8–C22) at the same conditions. Additional identification was provided by comparison to the mass spectral data of the authentic components through the AMDIS (Automated Mass Spectral Deconvolution and Identification), which was also used for accessing the Wiley Spectral Library collection and the NIST Library database (Gaithersburg, MD, USA; Wiley, Hoboken, NJ, USA). The EO samples were kept in amber glass vials for the biological analysis [61]. Compound identification was based on mass-spectral library matching (NIST and Wiley), experimentally determined Kováts retention indices, and comparison with literature retention indices.

### 4.4. In Silico Analysis of Target Prediction and Network Analysis

#### 4.4.1. Structural Preparation of Bioactive Compounds

The bioactive constituents of two *Thymus* essential oils *T. vulgaris* (TV) and *T. capitatus* (TC)—were identified by gas chromatography–mass spectrometry (GC-MS) as described previously. Canonical SMILES strings and three-dimensional structural data files (SDFs) for each compound were retrieved from the PubChem database (https://pubchem.ncbi.nlm.nih.gov, accessed on 2 March 2026) [62]. PubChem Compound Identifiers (CIDs) were cross-checked to confirm structural identity. In total, 39 compounds were resolved with verified structural representations. Compounds with zero relative abundance in a given essential oil were not considered constituents of that oil in the extract-specific network analysis; compounds detected in at least one of the two oils were retained in the integrated compound–target network.

#### 4.4.2. In Silico Compound–Target Prediction

Potential protein targets for each of the 39 compounds were predicted using six databases: SwissTargetPrediction (http://www.swisstargetprediction.ch, accessed on 28 February 2026; probability > 0) [63]; the Comparative Toxicogenomics Database (CTD; http://ctdbase.org, accessed on 28 February 2026; *Homo sapiens* associations) [64]; the Similarity Ensemble Approach (SEA; https://sea.bkslab.org, accessed on 28 February 2026; human-annotated entries) [65]; TargetNet (http://targetnet.scbdd.com, accessed on 28 February 2026; probability ≥ 0.5) [66]; SuperPred (https://prediction.charite.de, accessed on 28 February 2026) [67]; and BindingDB (https://www.bindingdb.org, accessed on 28 February 2026; IC_50_/K_d_/K_i_ ≤ 10,000 nM; *Homo sapiens* only) [68]. All target identifiers were converted to HGNC gene symbols for cross-database comparison, and duplicates were removed to produce a final compound–target edge list.

#### 4.4.3. Disease-Associated Gene Retrieval

Disease-associated genes were collected for 14 skin-related conditions (skin aging, photoaging, skin wrinkle, wound healing, psoriasis, dermatitis/atopic dermatitis, collagen disease, systemic scleroderma, acne, skin fibrosis, skin melanoma, keratosis, and related dermatological disorders) using Open Targets (https://platform.opentargets.org, accessed on 25 February 2026; association score > 0) [69], GeneCards (https://www.genecards.org, accessed on 25 February 2026; relevance score > 0) [70], MyGene.info (https://mygene.info, accessed on 25 February 2026; curated annotations only) [71], OMIM (https://omim.org, accessed on 25 February 2026; Mendelian disease entries) [72], DisGeNET (https://www.disgenet.org, accessed on 25 February 2026; score > 0) [73], MalaCards (https://www.malacards.org, accessed on 25 February 2026) [74], and DISEASES (https://diseases.jensenlab.org, accessed on 25 February 2026; JensenLab confidence > 0.5) [75]. All outputs were merged and deduplicated.

#### 4.4.4. Identification of Core Therapeutic Targets

Compound-predicted targets and disease-associated genes were intersected to identify genes present in both datasets. The resulting overlap was visualized as a Venn diagram.

#### 4.4.5. PPI Network Construction and Hub Gene Identification

The core therapeutic targets were submitted to STRING (version 12.0; https://string-db.org, accessed on 2 March 2026; confidence ≥ 0.400) [76] to construct a protein–protein interaction (PPI) network. The network was imported into Cytoscape (version 3.10) [77], and the top 50 hub genes were identified using the CytoHubba plugin with the Maximal Clique Centrality (MCC) algorithm [78].

#### 4.4.6. Gene Ontology and Reactome Pathway Enrichment Analysis

Gene Ontology (GO) enrichment analysis—Biological Process (BP), Molecular Function (MF), and Cellular Component (CC)—was carried out on the 50 hub genes using clusterProfiler (version 4.12) [79] with the org.Hs.eg.db annotation database. Reactome pathway enrichment was performed using ReactomePA (version 1.52.0) [80]. Statistical significance was set at adjusted *p* < 0.05 (Benjamini–Hochberg correction).

#### 4.4.7. Extract–Compound–Hub Target Sub-Network Construction

A tripartite sub-network was built to trace the relationship from the two plant extracts through their constituent compounds to the hub targets. Edges linked each extract (TV, TC) only to the compounds detected in it (GC–MS abundance > 0%; 28 compounds for TV and 36 for TC, of which 25 are shared), and from those compounds to the corresponding hub genes. Edges were not weighted by relative abundance. The network was laid out in Cytoscape with a force-directed algorithm. A Sankey diagram was also generated to visualize the pharmacological flow from extract to compound to pathway.

### 4.5. Molecular Docking

#### 4.5.1. Target Selection and Receptor Preparation

Rather than docking against all 50 Maximal Clique Centrality (MCC) hub genes (33 of which are predicted to interact with only 1–3 compounds), docking targets were selected based on compound-convergence degree, i.e., the number of unique *Thymus* compounds predicted to interact with each hub gene. The six targets with ≥10 predicted compound interactions were selected: NFKB1 (35 compounds), ESR1 (30), CASP9 (26), RELA (17), PTGS2 (11), and HSP90AA1 (10). Together, they account for 129 of the 235 compound–hub interactions (55%) and cover the inflammatory (NFKB1, RELA, PTGS2), oestrogen-receptor/ECM (ESR1) and stress/apoptosis (HSP90AA1, CASP9) axes. Crystal structures were downloaded from the Protein Data Bank (PDB; https://www.rcsb.org, accessed on 4 March 2026). PDB entries and native co-crystallized ligands are listed in Table S1.

Water molecules, ions, and co-crystallised buffer components were removed from each structure. Native ligands were extracted for grid-box definition where available. Receptors were prepared using AutoDockTools (MGLTools version 1.5.7; Scripps Research, La Jolla, CA, USA) [81] and converted to PDBQT format using Open Babel (version 3.1) [82].

#### 4.5.2. Ligand Selection and Preparation

For each of the six hub targets, only the compounds predicted by the network pharmacology pipeline to interact with that specific target were selected, yielding 129 compound–target docking pairs in total (NFKB1: 35; ESR1: 30; CASP9: 26; RELA: 17; PTGS2: 11; HSP90AA1: 10). 3D structures were downloaded from PubChem [62] (SDF format, record_type = 3d) where available; compounds lacking 3D PubChem records (trans-carveol, carotol, δ-cadinene, cis-sabinene hydrate, and ar-dihydro-turmerone) were generated from SMILES using RDKit (version 2024.03) with MMFF94 force-field optimisation. All structures were protonated at pH 7.4, at which all 39 constituents are neutral, and converted to PDBQT format using Open Babel (version 3.1) [82].

#### 4.5.3. Grid Box Definition and Docking Protocol

For targets with co-crystallised ligands (ESR1/OHT, CASP9/PHQ, PTGS2/RCX, and HSP90AA1/2KL), the binding site was defined as the pocket occupied by the native ligand, and the grid box was centred on the centre of mass of the native ligand in the relevant chain (chain A for ESR1, PTGS2, and HSP90AA1; chain E for CASP9). Box dimensions were set at 20 × 20 × 20 Å for ESR1 and HSP90AA1, and 22 × 22 × 22 Å for CASP9 and PTGS2. Grid centres for all six targets are listed in Table S1.

NFKB1 (1SVC) and RELA (1NFI) lack small-molecule co-crystals. For each, the grid was centred on the centre of mass of the protein residues that directly contact the co-crystallised DNA duplex (defined as any protein heavy atom within 4.5 Å of a DNA atom in the parent co-crystal structure). For NFKB1, 34 DNA-contact residues were identified from 1SVC (chain P vs. chain D), yielding a grid centred at (36.6, 24.4, 33.4 Å) with dimensions of 38 × 37 × 40 Å to fully envelop the DNA-binding groove. For RELA, 23 DNA-contact residues were mapped from PDB 1VKX (the NF-κB p50/p65 heterodimer–DNA co-crystal, chain B) onto 1NFI using residue-number offset alignment (offset = 319), yielding a grid centred at (2.2, 42.5, 20.6 Å) with dimensions of 40 × 45 × 75 Å to cover the extended Rel homology domain DNA-binding surface. The larger box dimensions for NFKB1 and RELA reflect the elongated, solvent-exposed geometry of protein–DNA interfaces compared to the compact ligand-binding pockets of the other four targets.

Docking was carried out with AutoDock Vina (version 1.2.7) [83] at an exhaustiveness of 128, generating 10 binding modes per run. A fixed random seed (42) was set for reproducibility. The docking protocol was validated by redocking the co-crystallised ligands of ESR1, PTGS2, and HSP90AA1, giving centre-of-mass RMSD values of 0.31, 0.29, and 1.39 Å, respectively (all <2.0 Å). A docking score of ≤−5.0 kcal/mol was used as a computational prioritisation threshold, not as a measure of statistical significance. Counts use unrounded scores. Since the essential oil constituents are predominantly monoterpenes (MW 136–154, 10–11 heavy atoms) and sesquiterpenes (MW 200–236, 14–16 heavy atoms), which are considerably smaller than typical drug molecules (MW 300–500), ligand efficiency (LE = −ΔG /N~heavy atoms~) was calculated for every pair to normalise binding energy by molecular size [84]. An LE ≥0.4 was considered excellent, and an LE ≥0.3 was considered a good hit, following the thresholds of Hopkins et al. [84].

### 4.6. Biological Activities of Essential Oils

#### 4.6.1. Collagenase Inhibitory Activity Assay

Collagenase inhibitory activity was assessed using the Collagenase Activity Assay Kit (ab196999, Abcam, Cambridge, UK) as previously described [49], based on hydrolysis of the synthetic collagen-mimetic substrate FALGPA (N-[3-(2-Furyl)acryloyl]-Leu-Gly-Pro-Ala). Extracts (4–1000 μg/mL) were incubated with collagenase and substrate, and absorbance was recorded kinetically at 345 nm (37 °C, 30 min); PBS-treated wells served as the enzyme (positive) control.

#### 4.6.2. Elastase Inhibitory Activity Assay

Elastase inhibitory activity was determined with the EnzChek^®^ Elastase Assay Kit (E-12056, Molecular Probes, Eugene, OR, USA) [85], based on digestion of quenched fluorescent DQ™-elastin by porcine pancreatic elastase (Ex/Em 485/530 nm). Diluted extracts were pre-incubated with elastase (15 min) before substrate addition, and IC_50_ values were derived from percentage inhibition relative to the enzyme control, with Daidzein as the reference standard.

#### 4.6.3. Tyrosinase Inhibitory Activity Assay

Tyrosinase inhibitory activity was determined using the Tyrosinase Inhibitor Screening Kit (K575-100, BioVision Inc., Milpitas, CA, USA) [86], which monitors mushroom-tyrosinase-catalysed chromophore formation at 510 nm. Samples were pre-incubated with the enzyme (10 min, 25 °C) before substrate addition, and absorbance was recorded kinetically; percentage inhibition was calculated from reaction-rate slopes relative to the enzyme control, using kojic acid as the positive control. All assays were performed in triplicate and expressed as mean ± SD.

#### 4.6.4. Hyaluronidase Inhibitory Activity Assay

Hyaluronidase inhibitory activity was evaluated turbidimetrically using the QuantiChrom™ Hyaluronidase Inhibitor Screening Assay Kit (DIHY-100, BioAssay Systems, Hayward, CA, USA) [87,88]. Samples were pre-incubated with hyaluronidase (15 min) before substrate addition, and residual (undegraded) hyaluronic acid was quantified at 600 nm after acid-mediated termination of the reaction, against no-enzyme and no-inhibitor controls.

#### 4.6.5. 5-Lipoxygenase (5-LOX) Inhibitory Activity Assay

5-Lipoxygenase (5-LOX) inhibitory activity was measured fluorometrically using the 5-Lipoxygenase Inhibitor Screening Kit (K980-100, BioVision Inc., Milpitas, CA, USA) [89], (Ex/Em 500/536 nm), with zileuton as the positive control. Percentage inhibition was calculated from fluorescence-rate slopes relative to the enzyme control.

#### 4.6.6. COX-2 Inhibitory Activity Assay

COX-2 inhibitory activity was evaluated using a fluorometric COX-2 Inhibitor Screening Kit (K547-100, BioVision Inc., Milpitas, CA, USA) [90], which detects COX-2-generated PGG_2_ via a fluorogenic probe (Ex/Em 535/587 nm). Serially diluted samples (0.01–100 μg/mL) were assayed against meloxicam as the positive control, and IC_50_ values were derived from concentration–response curves (triplicate, mean ± SD).

#### 4.6.7. DPPH Radical Scavenging Activity Assay

DPPH radical-scavenging activity was evaluated using a DPPH Antioxidant Assay Kit (K2078-100, BioVision Inc., Milpitas, CA, USA), based on reduction of the stable DPPH radical by antioxidant compounds [91]. Serial dilutions of the samples were incubated with a 600 μM DPPH working solution (10 min, room temperature, dark), and absorbance was measured at 517 nm. Percentage radical-scavenging activity, and IC_50_ values were calculated relative to ascorbic acid as the positive control [92].

#### 4.6.8. Ferric Reducing Antioxidant Power (FRAP) Assay

Ferric reducing antioxidant power was assessed using the FRAP Assay Kit (MAK369, Sigma-Aldrich, St. Louis, MO, USA), based on conversion of the ferric–tripyridyltriazine (Fe^3+^–TPTZ) complex to its ferrous form under acidic conditions [93,94]. Samples in DMSO were mixed with the FRAP reaction mixture (37 °C, 10 min, dark), and absorbance was read at 593 nm. Values were interpolated from an ascorbic acid calibration curve (0–0.5 mM) and expressed as mmol ascorbic acid equivalents (AAE)/g extract; all analyses were performed in triplicate (mean ± SD).

#### 4.6.9. Cell Culture and Cytotoxicity Assay

Human Skin Fibroblast (HSF) was obtained from Nawah Scientific Inc. (Mokatam, Cairo, Egypt). Cells were maintained in DMEM media supplemented with 100 μg /mL of streptomycin, 100 units/mL of penicillin and 10% of heat-inactivated foetal bovine serum in humidified, 5% (*v*/*v*) CO_2_ atmosphere at 37 °C.

Cell viability was assessed by the sulforhodamine B (SRB) assay [95,96]. Briefly, HSF cells (5 × 10^3^/well) were treated for 24 h with serial concentrations of TC oil, fixed with 10% TCA, stained with SRB (0.4% *w*/*v*), and solubilised with 10 mM Tris; absorbance was read at 540 nm (Infinite F50, TECAN, Männedorf, Switzerland), and IC_50_ was derived from the dose–response curve.

#### 4.6.10. Wound Healing (Scratch) Assay

The in vitro scratch-wound assay was performed in HSF cells with minor modifications to established protocols [97,98]. Confluent monolayers in collagen-coated 12-well plates were scratched with a sterile pipette tip, washed with PBS, and treated with TC oil (1.99 μg/mL) or medium alone (control). Wound width was measured at 0, 24, and 48 h using MII ImageView software (v3.7), and percentage closure was calculated as (A_t0h_ − A_t_) /A_t0h_ × 100, where A_t0h_ is the wound area at time zero and A_t_ is the area at time t (triplicate, mean ± SD).

#### 4.6.11. Quantitative Real-Time PCR (RT-qPCR) Analysis of Gene Expression

Total RNA was extracted from treated and untreated HSF cells using the RNeasy Mini Kit (Qiagen, Hilden, Germany) with on-column DNase I treatment. One-step RT-qPCR was performed using the iScript One-Step RT-PCR Kit with SYBR Green (Bio-Rad, Hercules, CA, USA) according to the manufacturer’s instructions, with the following modifications: annealing was performed at 58 °C, the extension step was at 72 °C for 30 s with fluorescence acquisition, and 40 cycles were used. Melt curve analysis confirmed a single product peak for each primer pair. Ct values were determined in the exponential phase and were below cycle 35. Primer efficiencies were 95.8–99.1% (Table S7), with R^2^ ≥ 0.994. *COL1A1*, *MMP1*, *ITGA11*, and *HAS2* were quantified using gene-specific primers (Table S6), with *GAPDH* as the endogenous reference. Relative expression was calculated by the efficiency-corrected E^−ΔΔCt^ method [99] from three independent biological replicates.

### 4.7. Chemometric Comparison with Literature Data

Essential-oil compositions of *T. capitatus* (31 datasets, including this study) and *T. vulgaris* (34 datasets, including this study) from the Mediterranean region and neighbouring areas were compiled from the literature (Table S8a,b). Eight major constituents (thymol, carvacrol, *p*-cymene, γ-terpinene, linalool, borneol, β-caryophyllene, and caryophyllene oxide) were used for *T. capitatus*, and four further chemotype markers (geraniol, sabinene hydrate, camphor, and 1,8-cineole) for *T. vulgaris* (Tables S9 and S10). Constituents not reported in a study were set to 0%. Datasets with incomplete or ambiguous compound identification (†) and commercial oils of unknown origin (‡) were excluded, leaving 28 populations per species. Percentages were square-root-transformed and mean-centred, and all chemometric analyses were performed in Python 3.12 using pandas 3.0 and NumPy 2.4 for data handling and scikit-learn 1.8 [100] for PCA. Hierarchical clustering used Ward’s method [101] with Euclidean distances on the same matrix (SciPy 1.17 [102]) and is displayed as dendrograms alongside heatmaps of the square-root-transformed percentages (Figure 7C,D). Figures were produced with Matplotlib 3.10. Chemotype was assigned by the dominant constituent; oils with thymol and carvacrol both ≥25% and within 1.5-fold of each other were classed as carvacrol/thymol. The chemotype counts in Section 3.1 were derived from the same 28 populations per species.

### 4.8. Statistical Analysis

Data are expressed as mean ± SD; GC–MS composition (Table 1) is given as mean ± SD of three replicate analyses. For the enzyme-inhibition and antioxidant assays (Table 2 and Table 3) and for RT-qPCR (Table 4), differences between groups were analysed by one-way ANOVA followed by Dunnett’s post hoc test (each oil versus its reference standard), with *T. capitatus* compared with *T. vulgaris* by Welch’s unpaired two-sided *t*-test; RT-qPCR comparisons (Table 4) were made on ΔCt values by Welch’s two-sided *t*-test. Scratch-wound width and percentage closure were compared between control and treated wells at each time point by Welch’s *t*-test. Statistical analysis was performed using GraphPad Prism version 8.0.2. (GraphPad Software, San Diego, CA, USA).

## 5. Conclusions

This study provides an integrated chemical, computational, and experimental characterisation of *Thymus vulgaris* and *Thymus capitatus* essential oils as multi-target dermatological agents. GC–MS identified 39 shared and species-specific volatile constituents dominated by thymol and carvacrol. Chemometric comparison with published oils classified the TC oil as a thymol chemotype, less common among regional populations (7 of 28 oils; Table S10), and the TV oil as a carvacrol-rich type, so chemotype should be specified when the oils are developed for topical use. Network pharmacology and molecular docking converged on NF-κB (NFKB1/RELA), ESR1, HSP90AA1, and PTGS2 as priority hub targets linking these constituents to skin-ageing and inflammatory pathways, and this was supported by the in vitro results: TC oil was the more potent inhibitor of elastase, tyrosinase, hyaluronidase, and 5-LOX, whereas TV oil was the stronger COX-2 inhibitor and antioxidant. At a low-toxicity concentration, TC oil was associated with accelerated fibroblast wound closure and with changes in COL1A1, MMP1, ITGA11, and HAS2 mRNA expression, suggestive of an extracellular-matrix-preserving, pro-regenerative profile that requires protein-level confirmation. Collectively, these findings support further investigation of combined *Thymus* species essential oils and suggest candidate molecular targets for their future development as cosmeceutical or adjunct dermatological agents. Further work should test these hub targets in cell-based reporter or knockdown systems, evaluate oil safety, stability, and nano-carrier delivery (e.g., liposomes, nanoemulsions) in topical formulations and assess efficacy in in vivo or ex vivo skin models.

## Figures and Tables

**Table 1:** Phytochemical composition of essential oils extracted from aerial parts of *T. capitatus* and *T. vulgaris* using gas chromatography/mass spectrometry (GC/MS) analysis (*n* = 3).

No.	Component Name	Chemical Structure	KI_Lit_	KI_Exp_	RT	*T. vulgaris*	*T. capitatus*	Molecular Formula
						Peak-Area %	Peak-Area %	
1	α-Thujene	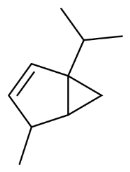	930	932	5.68	0.42 ± 0.01	0.32 ± 0.01	C_10_H_16_
2	α-Pinene	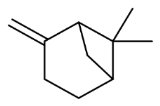	939	940	5.86	0.61 ± 0.02	0.38 ± 0.01	C_10_H_16_
3	Camphene	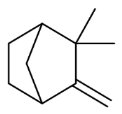	954	952	6.22	0.47 ± 0.01	0.31 ± 0.02	C_10_H_16_
4	(-)-β-Pinene	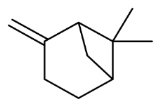	979	982	6.91	0.31 ± 0.01	0.3 ± 0.01	C_10_H_16_
5	β-Myrcene	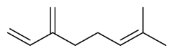	990	991	7.23	0.65 ± 0.01	0.39 ± 0.01	C_10_H_16_
6	α-Phellandrene	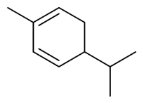	1002	1002	7.6	0.00	0.36 ± 0.01	C_10_H_16_
7	α-Terpinene	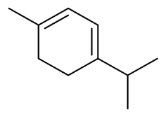	1017	1019	7.93	1.01 ± 0.02	1.08 ± 0.02	C_10_H_16_
8	P-Cymene	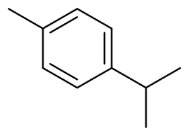	1024	1026	8.13	2.57 ± 0.03	5.21 ± 0.04	C_10_H_14_
9	D-Limonene	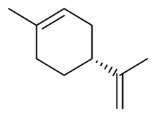	1029	1028	8.26	0.00	0.34 ± 0.01	C_10_H_16_
10	Eucalyptol	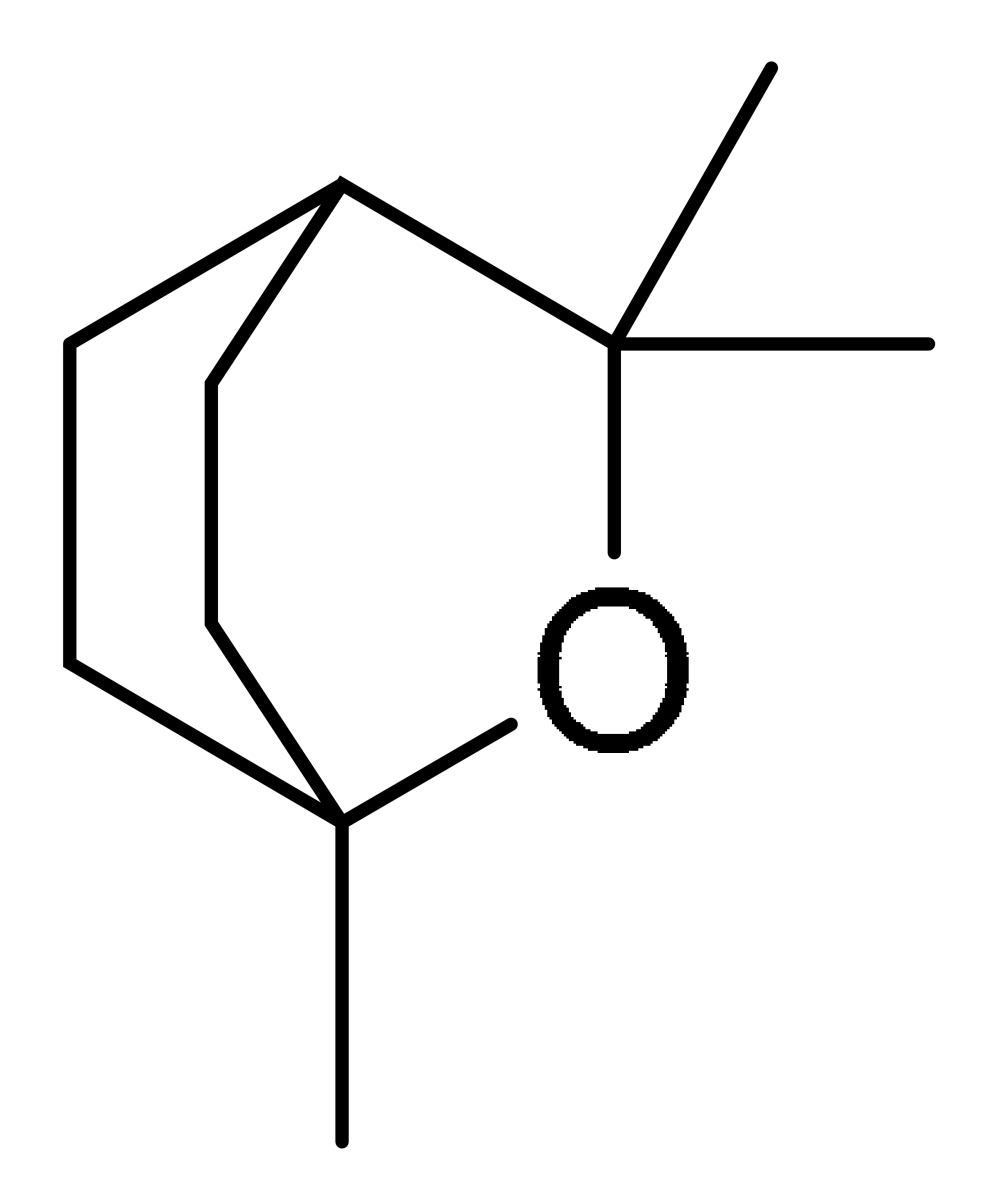	1031	1033	8.32	1.39 ± 0.02	0.99 ± 0.02	C_10_H_18_O
11	γ-Terpinene	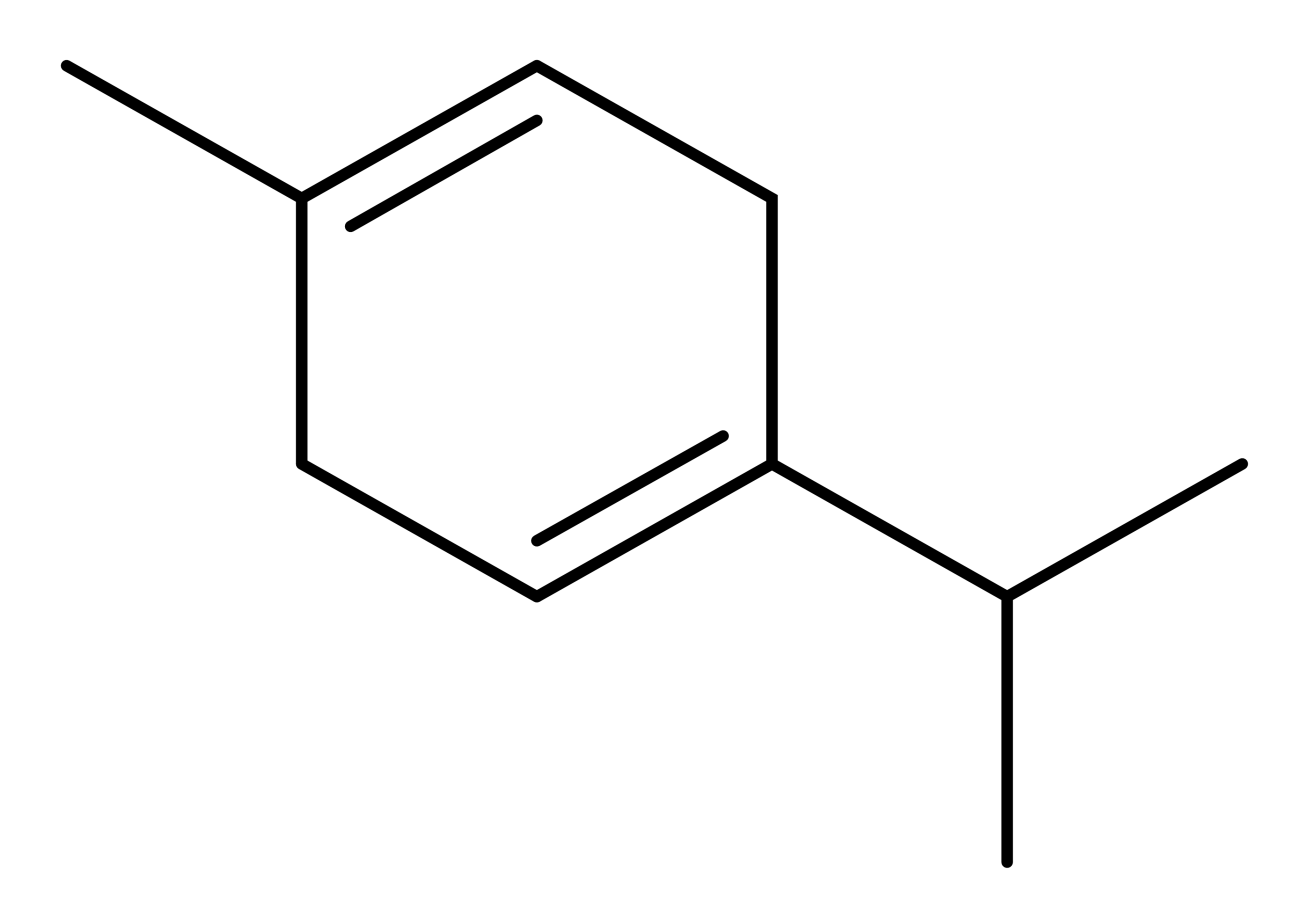	1059	1060	9.07	3.5 ± 0.03	8.3 ± 0.05	C_10_H_16_
12	cis-Sabinene hydrate	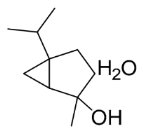	1070	1070	9.32	0.00	0.33 ± 0.01	C_10_H_18_O
13	Terpinolene	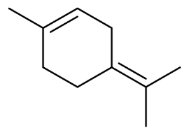	1088	1089	9.35	0.2 ± 0.01	0.29 ± 0.01	C_10_H_16_
14	Linalool	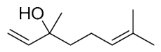	1096	1098	10.19	1.73 ± 0.02	0.61 ± 0.03	C_10_H_18_O
15	Borneol	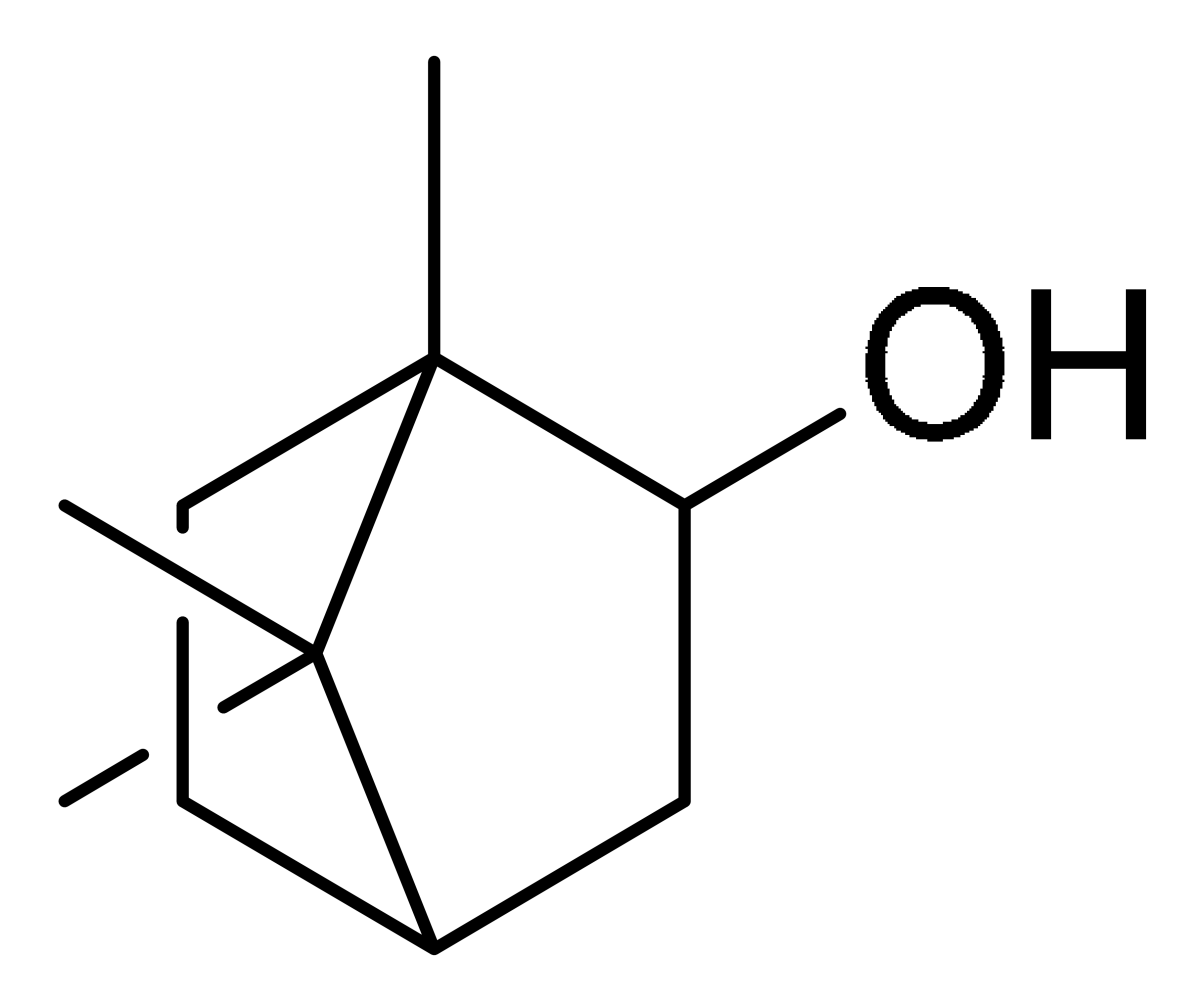	1169	1170	12.11	7.02 ± 0.04	0.62 ± 0.02	C_10_H_18_O
16	Terpinen-4-ol	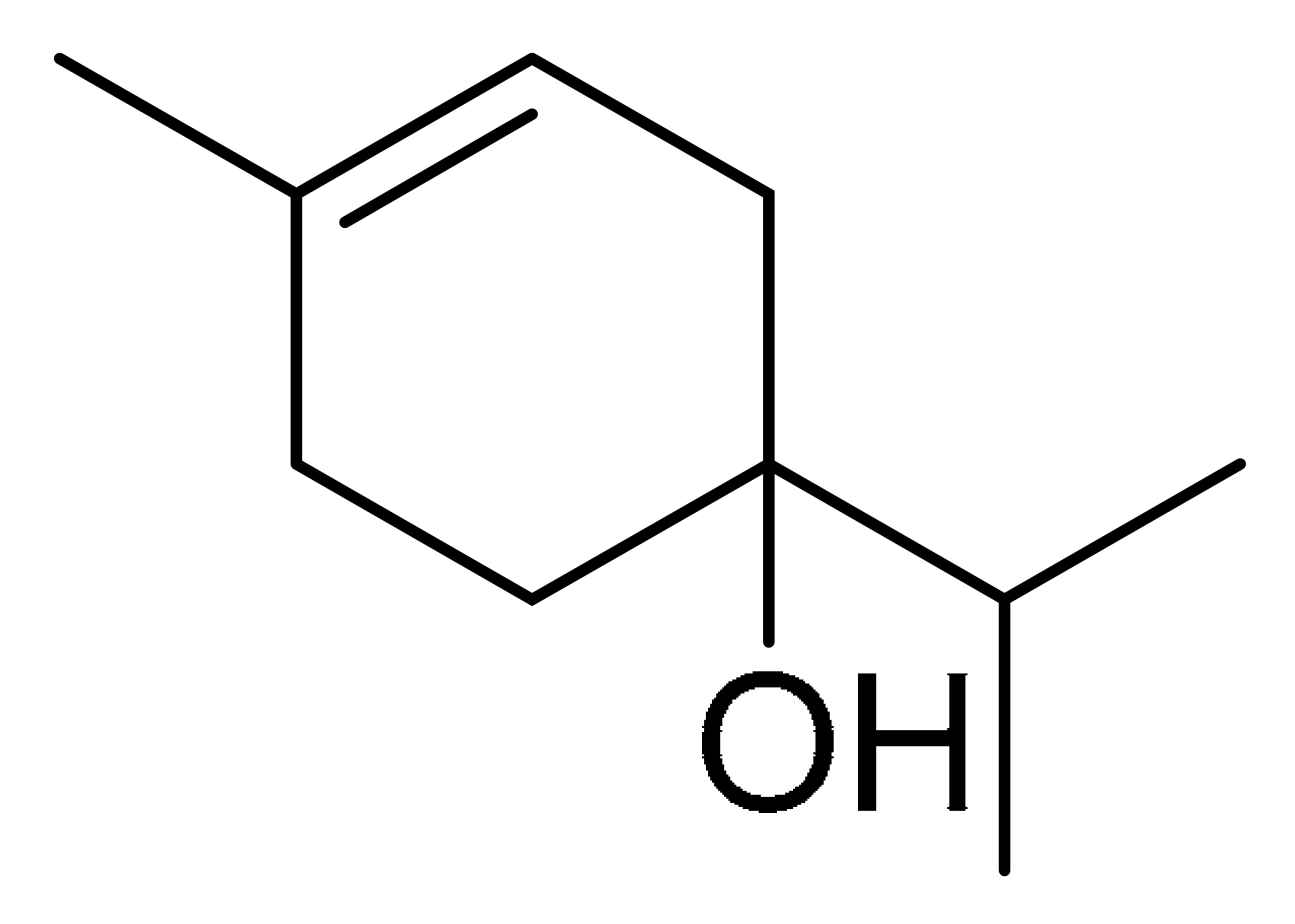	1177	1178	12.43	2.01 ± 0.03	1.08 ± 0.02	C_10_H_18_O
17	α-Terpineol	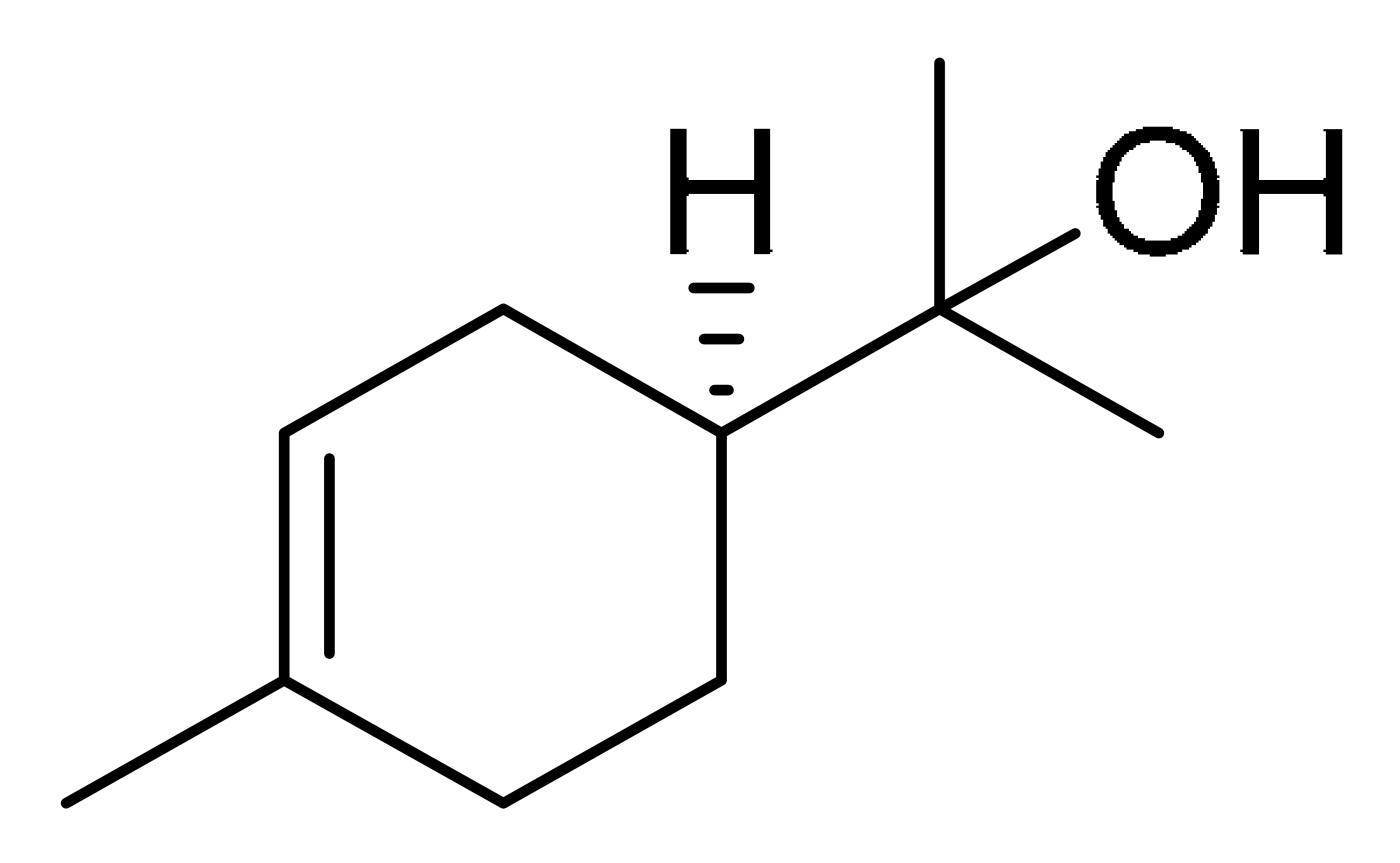	1188	1187	12.81	0.47 ± 0.01	0.27 ± 0.01	C_10_H_18_O
18	trans -Carveol	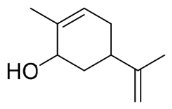	1216	1215	13.68	0.00	0.26 ± 0.02	C_10_H_16_O
19	p-Cumic aldehyde	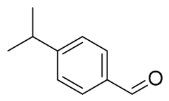	1238	1239	14.17	2.80 ± 0.02	4.31 ± 0.03	C_10_H_12_O
20	(-)-Carvone	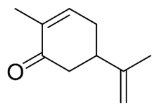	1243	1244	14.20	0.23 ± 0.01	0.00	C_10_H_14_O
21	Carvacrol Methyl Ether	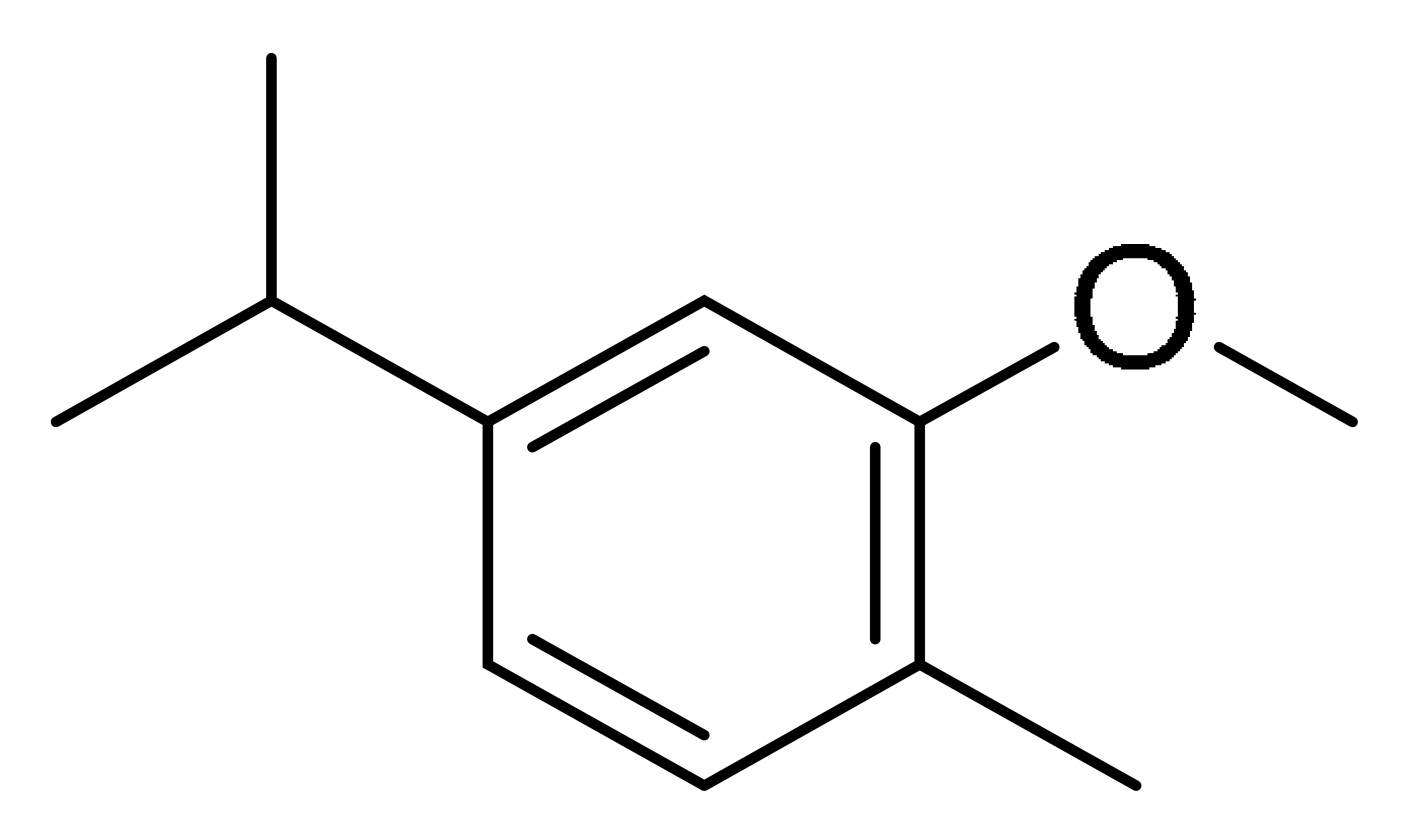	1244	1245	14.25	0.00	2.32 ± 0.03	C_11_H_16_O
22	Thymol	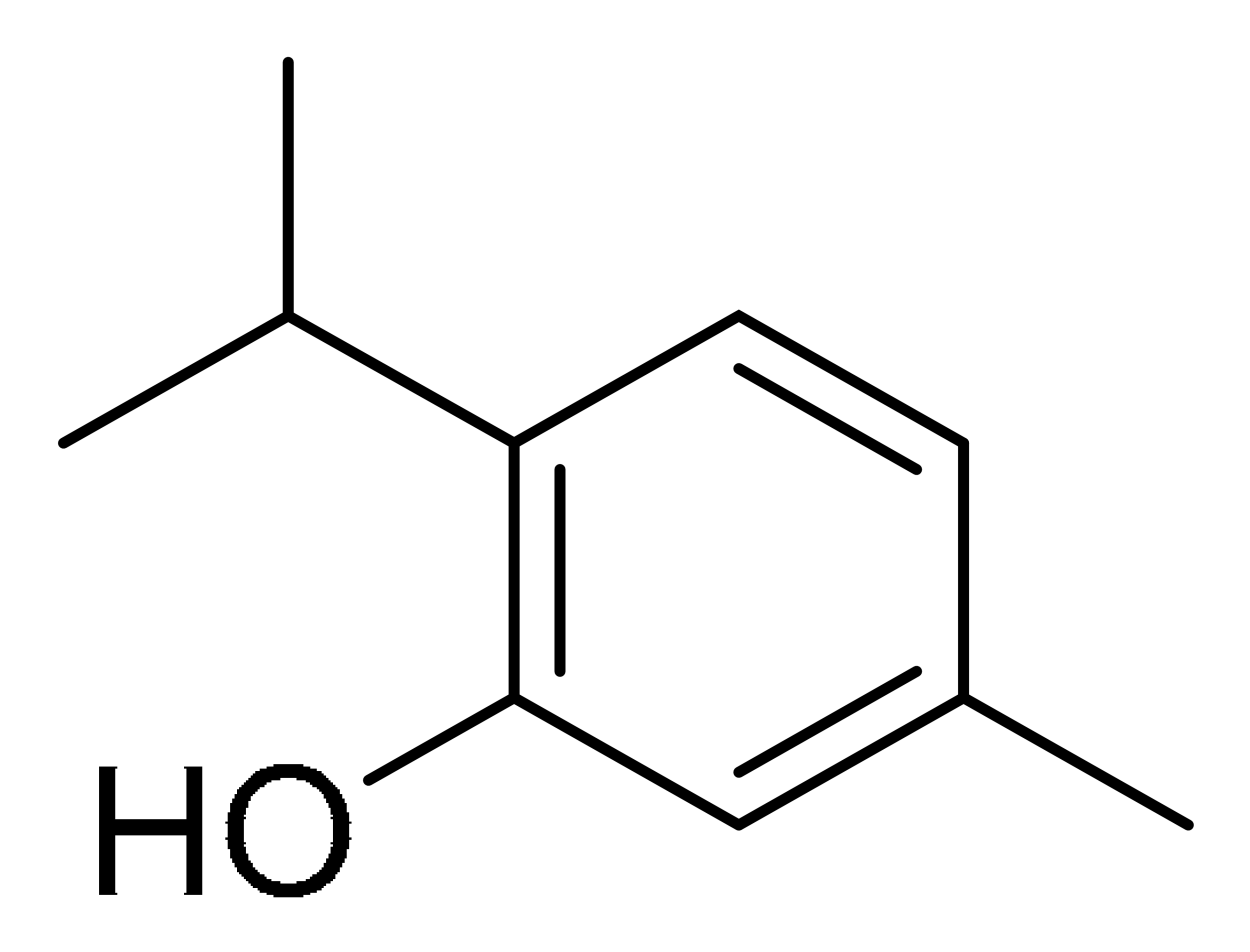	1290	1292	15.58	20.91 ± 0.2	41.06 ± 0.3	C_10_H_14_O
23	Carvacrol	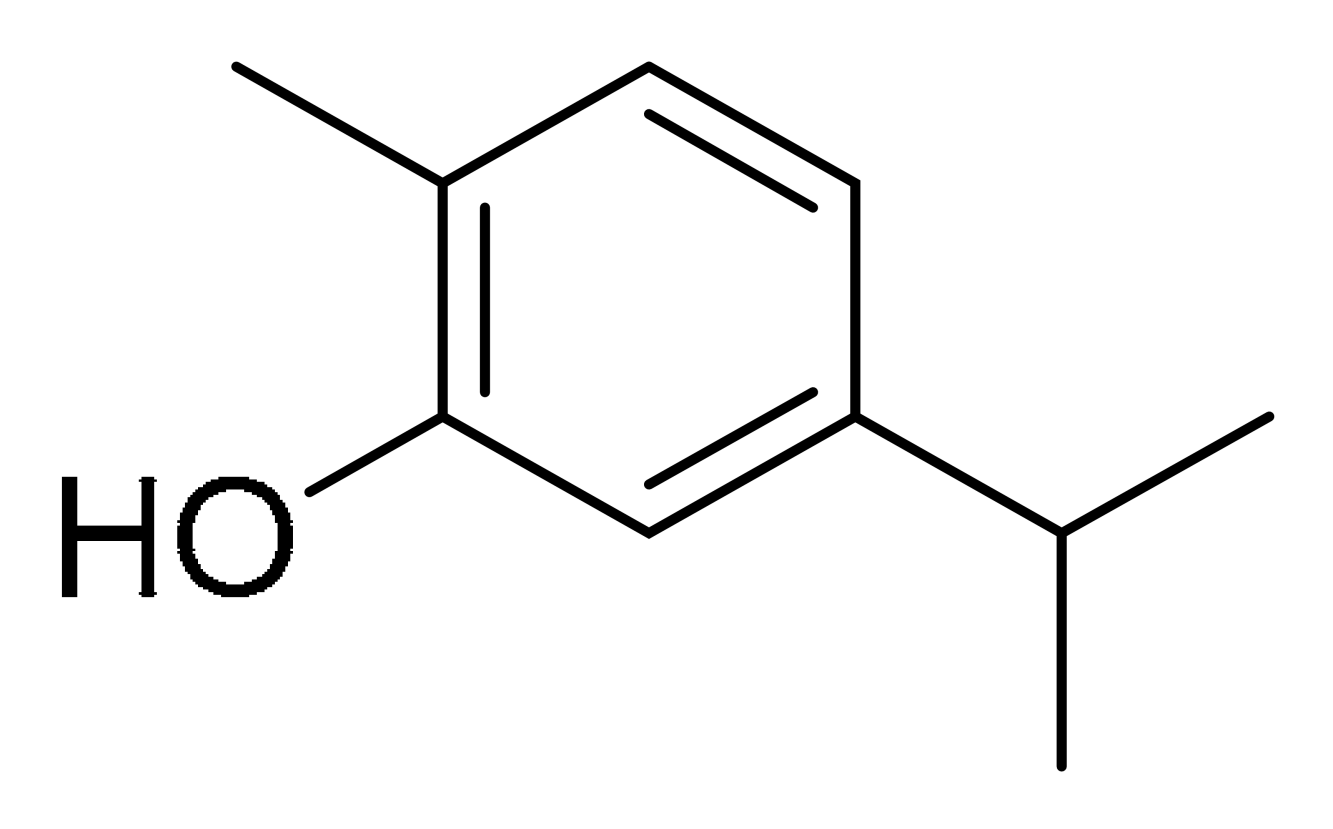	1299	1298	15.83	41.38 ± 0.3	13.02 ± 0.1	C_10_H_14_O
24	Eugenol	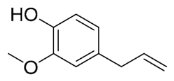	1359	1362	16.81	0.26 ± 0.01	0.00	C_10_H_12_O_2_
25	trans-Caryophyllene	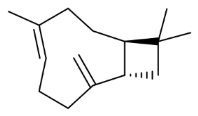	1408	1410	19.02	7.15 ± 0.03	5.1 ± 0.04	C_15_H_24_
26	Aromadendrene	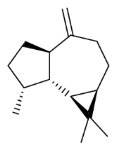	1441	1442	19.51	0.24 ± 0.01	0.45 ± 0.02	C_15_H_24_
27	α-Humulene	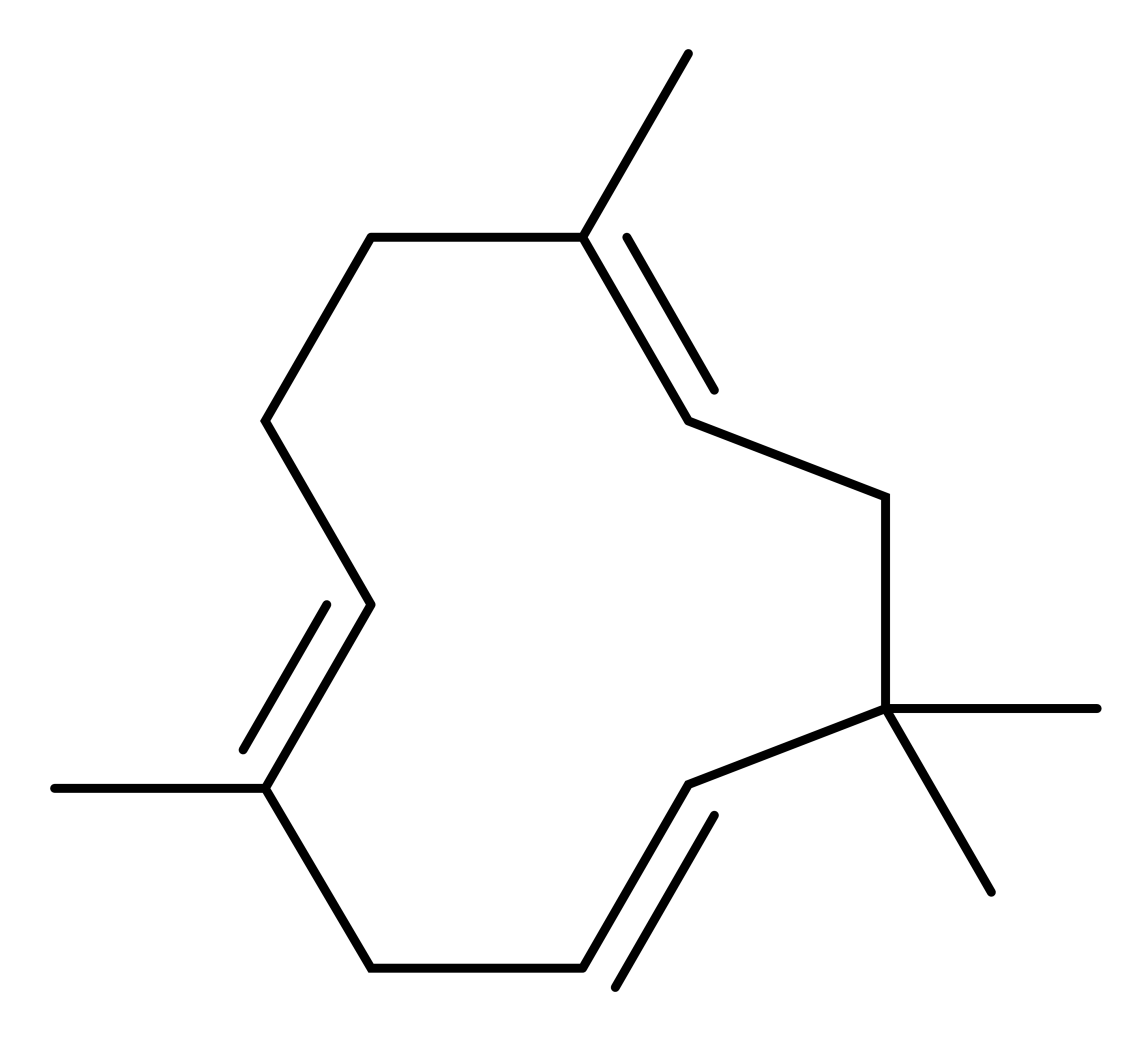	1454	1455	19.87	0.42 ± 0.01	0.48 ± 0.02	C_15_H_24_
28	α-Curcumene	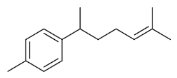	1480	1481	20.53	0.00	0.46 ± 0.02	C_15_H_22_
29	α-Zingiberene	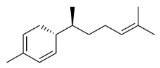	1493	1493	20.83	0.24 ± 0.01	0.39 ± 0.02	C_15_H_24_
30	Viridiflorene	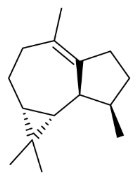	1496	1498	20.9	0.32 ± 0.01	0.7 ± 0.02	C_15_H_24_
31	β-Sesquiphellandrene	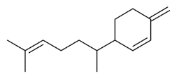	1522	1520	21.53	0.00	0.51 ± 0.02	C_15_H_24_
32	δ-Cadinene	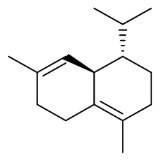	1523	1524	21.56	0.32 ± 0.01	0.00	C_15_H_24_
33	(-)-Spathulenol	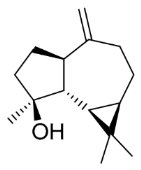	1578	1577	22.87	0.35 ± 0.01	0.61 ± 0.02	C_15_H_24_O
34	(-)-Caryophyllene oxide	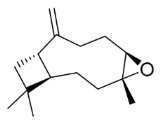	1583	1584	23.02	1.63 ± 0.01	4.1 ± 0.03	C_15_H_24_O
35	Carotol	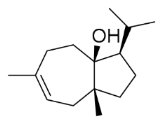	1594	1596	23.33	0.00	0.34 ± 0.01	C_15_H_26_O
36	Turmerone <ar-dihydro- >	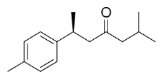	1595	1596	24.82	0.00	0.75 ± 0.02	C_15_H_22_O
37	Turmerone <ar->	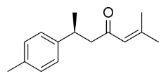	1669	1672	24.89	0.00	2.15 ± 0.02	C_15_H_20_O
38	Cedr-8-en-13-ol	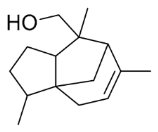	1689	1692	24.98	0.34 ± 0.01	0.33 ± 0.01	C_15_H_24_O
39	Curlone	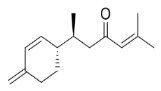	1997	1995	25.63	0.00	0.71 ± 0.01	C_15_H_22_O
	Monoterpene hydrocarbons (MH)					9.74	17.28	
	Oxygenated Monoterpenes (OM)					78.2	64.87	
	Sesquiterpene Hydrocarbons (SH)					8.69	8.09	
	Oxygenated Sesquiterpenes (OS)					2.32	8.99	
	Total					98.95	99.23	

KI_Lit_.: Kovats index reported in literature; KI_Exp_.: experimentally calculated Kovats index. Identification was performed via the comparison of the component mass spectral data and retention indices with those in (i) the NIST Mass Spectral Library, (ii) the Wiley Registry of Mass Spectral Data (8th edition, 2), and (iii) the literature.

**Table 2:** Enzyme inhibitory activities of essential oils IC_50_ (µg/mL).

Plant	Anti-Collagenase	Anti-Elastase	Tyrosinase	Anti-Hyaluronidase	5-LOX	COX-2
TV	70.53 ± 2.22	76.23 ± 2.03 #	20.53 ± 0.57	53.05 ± 1.63 §	1.733 ± 0.05 †	2.934 ± 0.085 ‡
TC	71.79 ± 2.29	42.96 ± 1.14 #,‖	15.4 ± 0.43 *,‖	20.82 ± 0.64 ‖	0.986 ± 0.03 †,‖	9.397 ± 0.271 ‡,‖
Piroxicam	63.06 ± 2.01	—	—	—	—	—
Daidzein	—	52.44 ± 1.4	—	—	—	—
Kojic acid	—	—	18.65 ± 0.52	—	—	—
Luteolin	—	—	—	17.81 ± 0.55	—	—
Zileuton	—	—	—	—	0.62 ± 0.02	—
Meloxicam	—	—	—	—	—	6.038 ± 0.174

Data are mean ± SD (*n* = 3). Each oil was compared with its respective reference standard using Dunnett’s test; *T. capitatus* versus *T. vulgaris* was compared using Welch’s unpaired two-sided *t*-test. Significance (*p* < 0.05) relative to the respective reference standard is indicated as * (kojic acid), # (daidzein), § (luteolin), † (zileuton), and ‡ (meloxicam); ‖ indicates a significant difference between *T. capitatus* and *T. vulgaris* (Welch’s *t*-test, *p* < 0.05). No symbol = not significant.

**Table 3:** Antioxidant activities (DPPH and FRAP assays) of essential oils.

Plant	DPPH IC_50_ (µg/mL)	FRAP (mmol AAE/g)
TV	29.71 ± 0.935 ***	0.029 ± 0.001
TC	36.99 ± 1.165 *** ‖	0.021 ± 0.0007 ‖
Ascorbic acid	18.75 ± 0.59	----

Data are mean ± SD (n = 3). One-way ANOVA followed by Dunnett’s test: *** *p* < 0.001 relative to ascorbic acid. *T. capitatus* versus *T. vulgaris* was compared using Welch’s unpaired two-sided *t*-test; ‖ indicates a significant difference between *T. capitatus* and *T. vulgaris* (*p* < 0.05). FRAP values are expressed as mmol ascorbic acid equivalents (AAE) per gram of essential oil. No symbol = not significant.

**Table 4:** Relative fold change in gene expression of HSFs treated with TC essential oil.

Gene Symbol	Fold Change (vs. Control), Mean ± SD
*COL1A1*	10.14 ± 0.15 ***
*MMP1*	0.34 ± 0.003 ***
*ITGA11*	2.94 ± 0.02 ***
*HAS2*	7.55 ± 0.33 ***

Data are mean ± SD, normalised to *GAPDH* (control = 1.000). ΔCt values were compared between treated and control cells using Welch’s two-sided *t*-test (*n* = 3); *** *p* < 0.001 versus the untreated control. Fold changes were calculated using the efficiency-corrected 2^−ΔΔCt^ method; primer amplification efficiencies are reported in Table S7 and Figure S6.

## Data Availability

The data presented in this study are available in the article and its Supplementary Materials.

## Supplementary Materials

The following supporting information can be downloaded at: https://www.mdpi.com/article/10.3390/plants15203238/s1. References [103,104,105,106,107,108,109,110,111,112,113,114,115,116,117,118,119,120,121,122,123,124,125,126,127,128,129,130,131,132] are cited in the supplementary materials.
