# Supplementary material for: Multi-Target Anti-Aging and Wound-Healing Potential of Libyan Thymus vulgaris and Thymus capitatus Essential Oils: Integrated GC–MS, Network Pharmacology, Molecular Docking, and In Vitro Evaluation
Source: Plants (Basel). 2026 Oct 21;15(20):3238. doi: 10.3390/plants15203238 (PMC13709056; doi:10.3390/plants15203238)
Supplement: Supplementary file 1 [file plants-15-03238-s001.zip › plants-4619784-supplementary.pdf]

Multi-Target Anti-Aging and Wound-Healing Potential of Libyan *Thymus vulgaris* and *Thymus capitatus* Essential Oils: Integrated GC–MS, Network Pharmacology, Molecular Docking, and In Vitro Evaluation

Sara Nasser Suliman[1,2], Anwagy Al-Masawri[3], Madiha A. Hassan[1], Sara Abouzeid[1*]

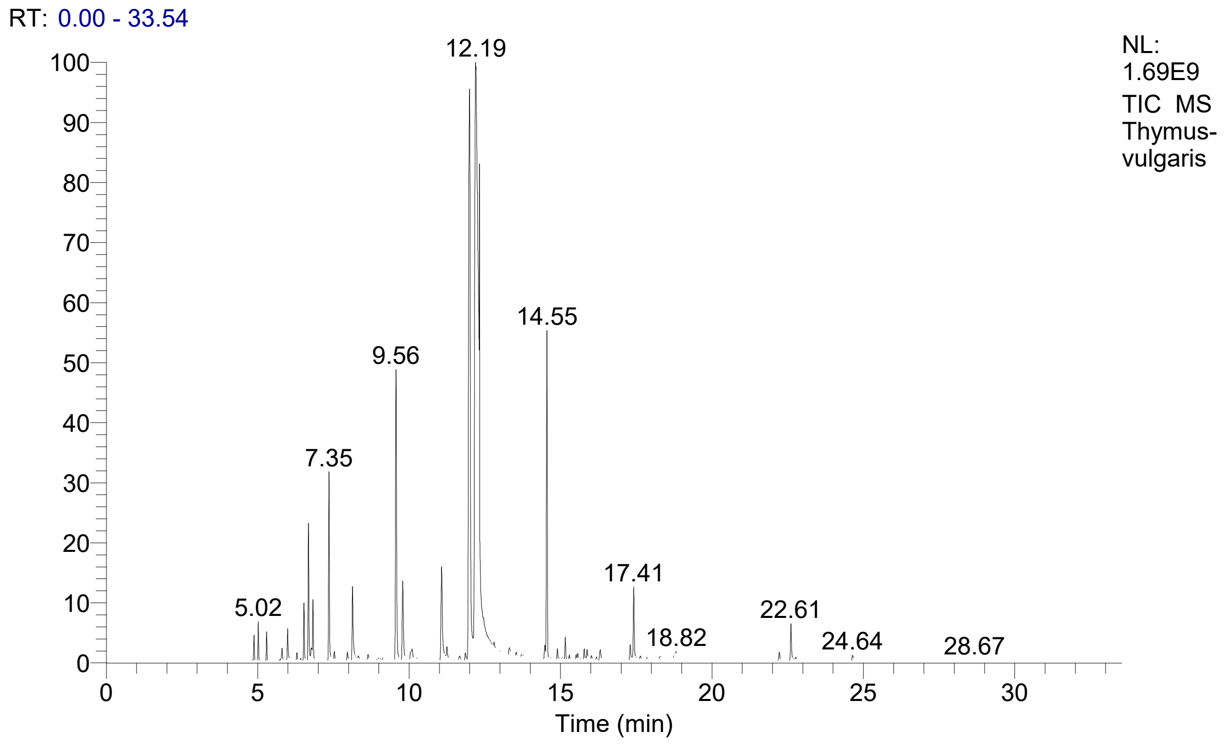

Figure S1. GC–MS total ion chromatogram of *Thymus vulgaris* essential oil.

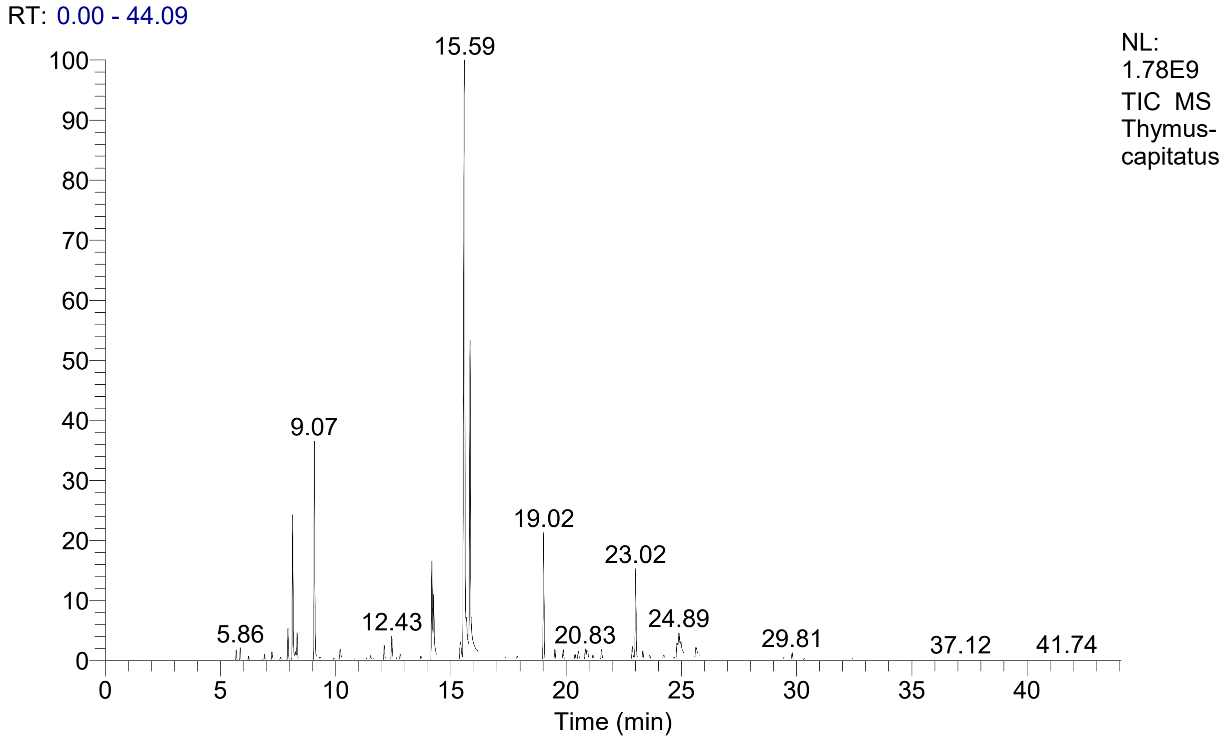

Figure S2. GC–MS total ion chromatogram of *Thymus capitatus* essential oil.

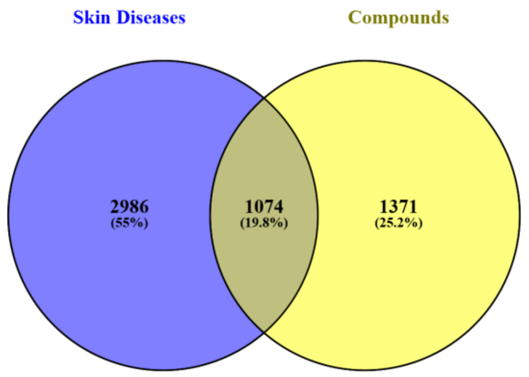

Figure S3. Venn diagram of compound-predicted targets (yellow; 2,445) and skin disease-associated genes (blue; 4,060), with 1,074 common targets (19.8%).

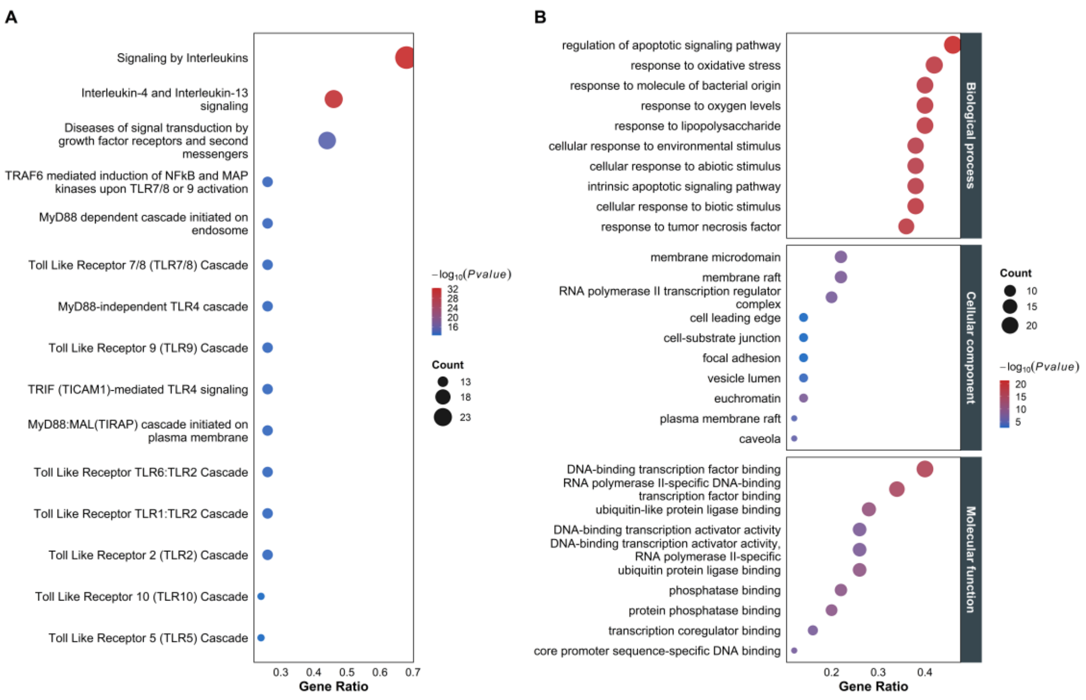

Figure S4. Enrichment analysis of the 50 hub genes (clusterProfiler/Reactome PA; Benjamini–Hochberg adjusted $P < 0.05$). **(A)** Top 15 Reactome pathways (dot size = gene count; colour = $-\log_{10}$ adjusted P). **(B)** Gene Ontology (GO) dot plots: Biological Process (top), Cellular Component (middle), Molecular Function (bottom); top 10 terms per ontology. Gene ratio = hub genes annotated to the term / hub genes with any annotation.

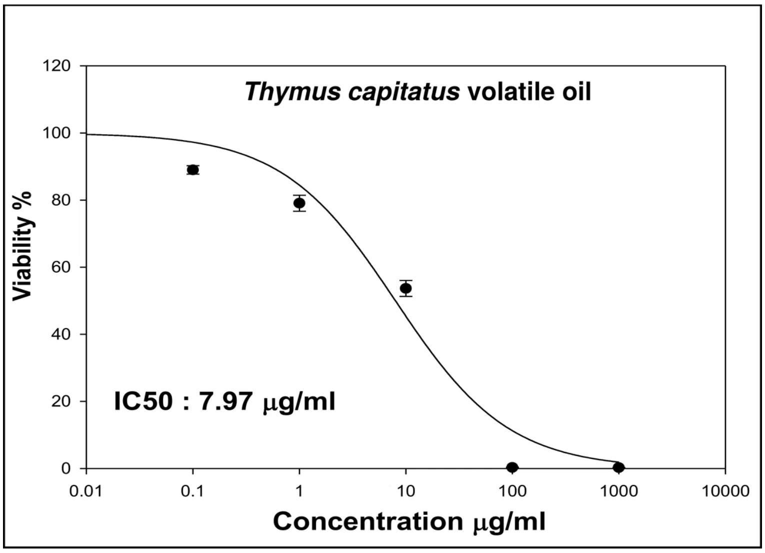

Figure S5. Dose-response curve representing the cytotoxic effect of *Thymus capitatus* (TC) volatile oil against (HSF) cells using the SRB assay. The determined IC50 value is 7.97 µg/ml.

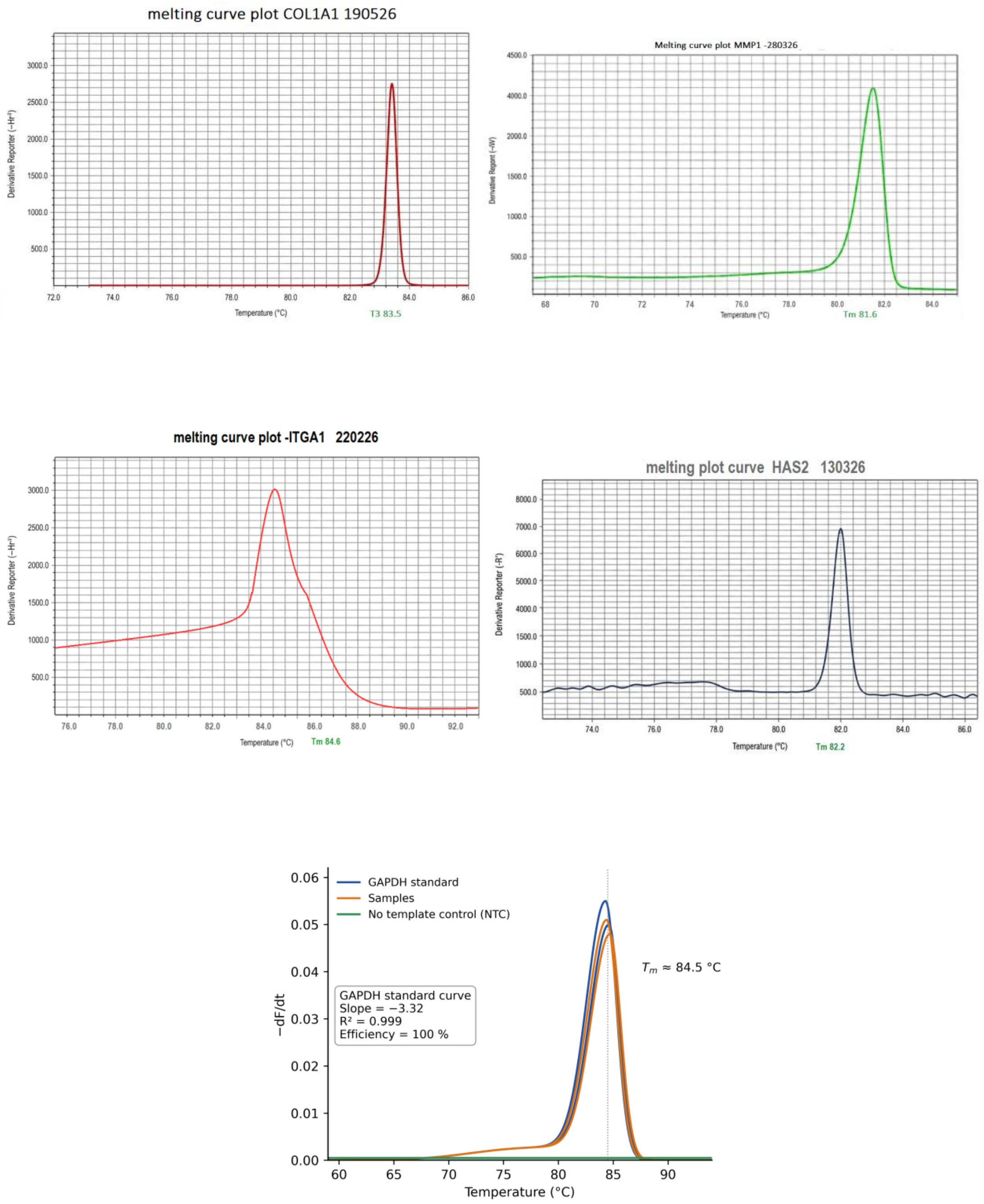

Figure S6. Melting-curve plots of the RT-qPCR primer pairs for COL1A1, MMP1, ITGA11, HAS2 and GAPDH (top to bottom); each shows a single product peak. Melting temperatures are given in Table S7.

Table S1. Target protein structures used for molecular docking. *Targets without a co-crystallized small-molecule ligand; grid centred on DNA-contact residue centre-of-mass. Redock COM RMSD, distance between the mass-weighted heavy-atom centres of the top-ranked redocked pose and the crystallographic ligand; n/a, redocking not applicable (no small-molecule co-crystal, or covalent peptide ligand).

Target	PDB ID	Chain(s)	Native Ligand	Grid Box (Å)	Grid centre x, y, z (Å)	Redock COM RMSD (Å)
NFKB1	1SVC	P	None*	38×37×40	36.550, 24.362, 33.375	n/a
ESR1	3ERT	A	OHT	20×20×20	31.575, −1.590, 25.599	0.31
CASP9	1JXQ	A, B	PHQ	22×22×22	23.965, 32.444, −18.518	n/a
RELA	1NFI	A	None*	40×45×75	2.206, 42.455, 20.646	n/a
PTGS2	5KIR	A	RCX	22×22×22	23.206, 1.318, 34.259	0.29
HSP90AA1	2WI7	A	2KL	20×20×20	2.172, 8.672, 25.731	1.39

Table S2. The top 50 hub genes (CytoHubba MCC) sorted by Compound Degree (number of Thymus compounds targeting each gene). Topological metrics (PPI Degree, Betweenness Centrality, Closeness Centrality, and Clustering Coefficient) are derived directly from the full 1,068-node STRING protein–protein interaction network.

Gene	Compound Degree	PPI Degree	Betweenness Centrality	Closeness Centrality	Clustering Coefficient	Functional Class
NFKB1	35	201	0.01428	0.56587	0.3237	Transcription factor
ESR1	30	222	0.02800	0.57837	0.2659	Nuclear receptor
CASP9	26	111	0.00340	0.51479	0.5207	Protease (apoptosis)
RELA	17	153	0.01162	0.54057	0.3483	Transcription factor
PTGS2	11	161	0.01399	0.54299	0.3355	Cyclooxygenase
HSP90AA1	10	246	0.04437	0.59328	0.2184	Chaperone

Gene	Compound Degree	PPI Degree	Betweenness Centrality	Closeness Centrality	Clustering Coefficient	Functional Class
APP	8	152	0.01437	0.54033	0.2887	Amyloid precursor
NFE2L2	7	125	0.00627	0.52276	0.4063	Transcription factor
TNF	5	310	0.06455	0.62218	0.1850	Cytokine
STAT3	5	245	0.02479	0.58711	0.2637	Transcription factor
MAPK14	5	114	0.00551	0.51854	0.4544	Serine/threonine kinase
AKT1	4	350	0.08315	0.64413	0.1678	Serine/threonine kinase
EGFR	4	284	0.05323	0.61109	0.2023	Receptor tyrosine kinase
SIRT1	4	171	0.01250	0.55103	0.3306	Deacetylase
ICAM1	4	134	0.00430	0.52526	0.4337	Cell adhesion
JAK2	4	131	0.00420	0.52729	0.4418	Non-receptor tyrosine kinase
HMOX1	4	107	0.00563	0.51143	0.4507	Oxidoreductase
TP53	3	381	0.13000	0.65943	0.1495	Tumour suppressor
ALB	3	258	0.04594	0.59719	0.2296	Transport protein
MAPK3	3	211	0.01675	0.57181	0.2858	Serine/threonine kinase
PPARG	3	199	0.02063	0.56439	0.2730	Nuclear receptor
MMP9	3	185	0.01087	0.55330	0.3409	Metalloproteinase
CTNNB1	2	278	0.04433	0.60687	0.2107	Signalling / adhesion
SRC	2	251	0.05595	0.59289	0.2141	Non-receptor tyrosine kinase
JUN	2	246	0.02626	0.58993	0.2494	Transcription factor
HIF1A	2	239	0.02257	0.58704	0.2591	Transcription factor
BCL2	2	234	0.01884	0.58508	0.2747	Anti-apoptotic

Gene	Compound Degree	PPI Degree	Betweenness Centrality	Closeness Centrality	Clustering Coefficient	Functional Class
CASP3	2	229	0.02179	0.58211	0.2840	Protease (apoptosis)
GSK3B	2	184	0.02005	0.55970	0.3052	Serine/threonine kinase
FOS	2	182	0.01741	0.55283	0.2953	Transcription factor
CDKN1A	2	140	0.00570	0.53330	0.4030	Cell cycle regulator
ACTB	1	360	0.09768	0.64967	0.1643	Cytoskeletal protein
MYC	1	319	0.08086	0.62937	0.1792	Transcription factor
IL1B	1	235	0.02252	0.58227	0.2510	Cytokine
FN1	1	208	0.03708	0.56790	0.2576	ECM glycoprotein
CDH1	1	188	0.01473	0.55712	0.3137	Cell adhesion
H3-3B	1	176	0.02047	0.55275	0.2847	Histone
ATM	1	173	0.01482	0.54681	0.2996	Serine/threonine kinase
MTOR	1	167	0.00945	0.54970	0.3710	Serine/threonine kinase
TLR4	1	162	0.01232	0.54260	0.3629	Toll-like receptor
CREB1	1	161	0.01430	0.54408	0.3220	Transcription factor
CCL2	1	156	0.00661	0.53565	0.3487	Cytokine/chemokine
CXCL8	1	154	0.00883	0.53627	0.3510	Cytokine/chemokine
STAT1	1	153	0.00839	0.53908	0.3770	Transcription factor
NFKBIA	1	149	0.00744	0.53822	0.3781	NF-κB inhibitor
FOXO1	1	134	0.00549	0.52752	0.3994	Transcription factor
MMP2	1	126	0.00400	0.52049	0.4527	Metalloproteinase
MAPK8	1	126	0.00684	0.52619	0.4310	Serine/threonine kinase

Gene	Compound Degree	PPI Degree	Betweenness Centrality	Closeness Centrality	Clustering Coefficient	Functional Class
IL1A	1	125	0.00267	0.51690	0.4400	Cytokine
IKBKB	1	99	0.00476	0.50909	0.4762	Serine/threonine kinase

Table S3. Binding affinities (kcal/mol) of the top-performing Thymus compounds against the six compound-convergence hub targets. Docking: AutoDock Vina 1.2.7, exhaustiveness = 128, seed = 42.

Compound	HSP90AA1	ESR1	PTGS2	RELA	NFKB1	CASP9
Viridiflorene	-9.0	-7.8	-7.5	-6.1	-5.8	-5.3
delta-Cadinene	-8.7	-7.8	-8.2	-5.5	-5.5	-5.3
Aromadendrene	-8.5	-7.9	-7.3	-5.9	-5.5	-5.4
alpha-Humulene	-7.4	-8.5	-6.5	-5.7	-5.5	-5.6
trans-Caryophyllene	-7.8	-8.4	-6.9	-5.8	-5.4	-5.6
Caryophyllene oxide	-7.6	-8.4	-7.4	-5.8	-5.2	-5.5
ar-dihydro-Turmerone	-7.7	-7.3	-8.2	-5.6	-5.8	-4.9
Cedr-8-en-13-ol	-8.2	-7.8	-7.3	-5.8	-5.8	-5.6
alpha-Curcumene	-8.1	-7.3	-7.6	-5.7	-5.9	-5.2
Carotol	-6.9	-7.4	-8.1	-5.9	-6.0	-5.2
alpha-Zingiberene	-8.1	-7.0	-7.9	-4.7	-5.7	-4.5
Curlone	-8.0	-7.1	-8.0	-5.4	-6.1	-4.4

Table S4: Effect of *Thymus capitatus* volatile oil on HSF cell viability**.**

**Concentration (µg/ml)**	**Cell Viability (% ± SD)**
0 (Control)	100.00 ± 0.00
0.1	89.00 ± 1.25
1	79.06 ± 2.38
10	53.68 ± 2.40
100	0.28 ± 0.09

1000	0.25 ± 0.02

Data are presented as mean ± standard deviation (SD)

Table S5. Effect of *Thymus capitatus* volatile oil (1.99 µg/mL, 1/4 × IC_{50}) on HSF scratch-wound width over time, compared to untreated control (n = 3 wells per group).

Time (h)	TC-treated (mean width ± SD, mm)	Control (mean width ± SD, mm)
0	0.91 ± 0.02	0.94 ± 0.02
24	0.53 ± 0.0141	0.67 ± 0.0173
48	0.26 ± 0.0141	0.41667 ± 0.0153

Table S6. Characteristics and sequences of primers used for RT-qPCR analysis of target and reference genes.

Gene	Accession No.	Primer Sequence 5′→3′	Product Size	Note
**GAPDH**	NM_002046.7	F: 5'-GTCTCCTCTGACTTCAACAGCG-3'<br>R: 5'-ACCACCCTGTTGCTGTAGCCAA-3'	197 bp	Housekeeping
**COL1A1**	NM_000088.3	F: 5'- GATTCCCTGGACCTAAAGGTGC -3'<br>R: 5'- AGCCTCTCCATCTTTGCCAGCA-3'	112 bp	Collagen I α1 chain
**MMP1**	NM_002421.4	F: 5'- ATGAAGCAGCCCAGATGTGGAG -3'<br>R: 5'- TGGTCCACATCTGCTCTTGGCA-3'	96 bp	Matrix metalloproteinase-1
**ITGA11**	NM_001004439.2	F: 5'- GCCTCCAGTATTTTGGCTGCAG -3'<br>R: 5'- GCTCAAAGTGGAGGCTGGCATT -3'	101 bp	Integrin α11
**HAS2**	NM_005328.3	F: 5'- GTCATGTACACAGCCTTCAGAGC -3'<br>R: 5'- ACAGATGAGGCTGGGTCAAGCA -3'	118 bp	Hyaluronan synthase 2

Table S7. Primer validation parameters: melting temperature (Tm), amplification efficiency (E), standard-curve slope and R^2.

Gene Target	Full Name	Tm (°C)	Efficiency (E)	Slope	R^2
**COL1A1**	Collagen Type I Alpha 1	~83.5	98.2%	-3.366	0.998
**MMP1**	Matrix Metalloproteinase-1	~81.0	96.5%	-3.409	0.995

**ITGA11**	Integrin Subunit Alpha 11	~84.8	99.1%	-3.343	0.999
**HAS2**	Hyaluronan Synthase 2	~82.2	95.8%	-3.427	0.994
**GAPDH**	Glyceraldehyde 3-phosphate dehydrogenase	~84.5	100%	-3.32	0.999

Chemical Composition of Essential Oils from *Thymus capitatus* and *Thymus vulgaris* Across Different Geographical Locations

Table S8a. Chemical composition (%) of ***Thymus capitatus*** essential oils from different countries and plant parts

Compound	Libya (our study)	Tunisia Kairouan [S9]	Morocco [S15]	Portugal [S22]	Libya Msallata [S4]	Italy [S20]	Libya Zintan [S3]	Algeria [S14]	Palestine Ramallah [S16]	Palestine Jenin [S16]	Palestine Hebron [S16]
**Monoterpene hydrocarbons**											
α-Thujene	**0.32±0.01**	0.54	-	0.4	-	0.7	0.67	0.38	0.65	0.31	-
α-Pinene	**0.38±0.01**	0.38	1.75	0.3	0.99	0.6	0.49	0.91	0.70	0.49	2.52
Camphene	**0.31±0.02**	-	0.38	-	-	0.2	0.33	0.2	0.15	0.90	5.56
β-Pinene	**0.3±0.01**	-	-	0.3	-	0.4	0.33	-	-	0.49	0.23
β-Myrcene	**0.39±0.01**	0.87	-	1.1	1.94	1.7	0.91	1.49	0.73	0.33	0.17
α-Phellandrene	**0.36±0.01**	-	-	0.1	-	0.2	0.12	-	0.08	-	-
α-Terpinene	**1.08±0.02**	1.11	1.50	0.9	2.37	1.2	0.75	1.78	2.25	1.41	0.35
p-Cymene	**5.21±0.04**	6.25	-	4.8	9.51	4.4	3.24	-	-	1.41	8.41
o-Cymene	**-**	-	5.12	-	-	-	-	-	16.84	-	-
Limonene	**0.34±0.01**	-	-	0.1	-	0.4	-	-	-	-	0.47
γ-Terpinene	**8.3±0.05**	6.75	-	-	8.39	3.7	3.09	10.30	30.94	67	3.61
Terpinolene	**0.29±0.01**	0.26	4.33	-	-	0.2	0.10	-	-	-	-
**Oxygenated monoterpenes**											
1,8-Cineole	**0.99±0.02**	-	-	-	-	-	0.08	-	-	-	-
cis-Sabinene hydrate	**0.33±0.01**	-	-	-	-	-	0.29	-	-	-	-
Linalool	**0.61±0.03**	1.51	-	-	3.43	0.9	1.21	2.29	6.19	-	-
Borneol	**0.62±0.02**	-	-	-	1.27	0.4	1.58	-	-	-	-
Terpinen-4-ol	**1.08±0.02**	1.40	-	-	1.49	0.8	0.65	-	-	-	0.02
α-Terpineol	**0.27±0.01**	-	-	-	-	0.1	-	0.37	-	-	0.23
cis-β-Terpineol	**-**	-	-	-	-	-	-	-	0.02	12.91	-
trans-Carveol	**0.26±0.02**	-	-	-	-	-	-	-	-	-	-
p-Cumic aldehyde	**4.31±0.03**	-	-	-	-	-	-	-	-	-	-
Thymol	**41.06±0.3**	1.35	-	-	6.54	0.3	12.27	51.22	3	5.51	40.35
Carvacrol	**13.02±0.1**	65.38	79.22	-	56.27	79.9	68.07	12.59	31.25	6.44	0.58
Thymol methyl ether	**-**	-	-	-	-	-	-	-	0.24	-	-
Carvacrol methyl ether	**2.32±0.03**	-	-	-	-	-	-	-	-	0.07	10.70
Carvacryl acetate	**-**	0.45	-	-	1.81	-	-	-	-	-	-
**Sesquiterpenes**											
β-Caryophyllene	**5.1±0.04**	4.94	4.31	-	3.57	2.3	1.01	2.01	4.61	1.65	13.23
α-Humulene	**0.48±0.02**	0.10	-	-	-	0.1	0.05	-	-	-	-
Aromadendrene	**0.45±0.02**	-	-	-	-	0.1	-	-	-	-	-
Viridiflorene	**0.7±0.02**	-	-	-	-	-	-	0.34	0.27	-	-
Spathulenol	**0.61±0.02**	-	-	-	-	-	-	-	-	-	-
Caryophyllene oxide	**4.1±0.03**	-	0.81	-	-	0.2	0.51	1.21	0.77	0.11	1.80
α-Bisabolol	**-**	0.35	-	-	-	0.2	-	-	-	-	-
α-Curcumene	**0.46±0.02**	-	-	-	-	-	-	-	-	-	-
α-Zingiberene	**0.39±0.02**	-	-	-	-	-	-	-	-	-	-
β-Sesquiphellandrene	**0.51±0.02**	-	-	-	-	-	-	-	-	-	-
Carotol	**0.34±0.01**	-	-	-	-	-	-	-	-	-	-
ar-Turmerone	**2.15±0.02**	-	-	-	-	-	-	-	-	-	-
ar-Dihydroturmerone	**0.75±0.02**	-	-	-	-	-	-	-	-	-	-
Cedr-8-en-13-ol	**0.33±0.01**	-	-	-	-	-	-	-	-	-	-
Curlone	**0.71±0.01**	-	-	-	-	-	-	-	-	-	-
**Others**											
Eugenol	**-**	0.21	-	-	-	-	-	-	-	-	-
Durenol (2,3,5,6-tetramethylphenol)	**-**	-	-	-	-	-	-	-	-	-	-

Table S8 (continue)

Compound	Libya (our study)	East Libya Sidi-Alhamry [S1]	East Libya Abu-draa [S1]	Egypt North coast [S5]	Tunisia Matmata [S7]	Libya Al Jabal Alkhadr [S2]	Greece [S18]	Tunisia Jendouba/Haouaria [S12]	Italy Lascari [S19]	Tunisia Zaghouan [S10]	Cyprus [S17]
**Monoterpene hydrocarbons**											
α-Thujene	**0.32±0.01**	7.93	1.64	-	-	1.64	0.2	1.3	2.4	0.5	1.45
α-Pinene	**0.38±0.01**	-	-	-	-	-	2.8	0.8	2.6	-	1.22
Camphene	**0.31±0.02**	-	-	-	-	-	1.6	0.3	0.2	0.1	1.05
β-Pinene	**0.3±0.01**	-	-	-	-	-	0.3	0.1	0.5	-	-
β-Myrcene	**0.39±0.01**	-	-	-	-	-	1.0	2.2	2.1	0.7	1.89
α-Phellandrene	**0.36±0.01**	-	-	-	-	-	0.2	0.3	0.3	-	0.44
α-Terpinene	**1.08±0.02**	-	-	-	0.72	-	0.4	1.9	0.2	1.0	1.81
p-Cymene	**5.21±0.04**	3.71	-	-	5.04	-	31.0	9.2	22.8	5.3	9.94
o-Cymene	**-**	-	-	-	-	-	-	-	-	-	-
Limonene	**0.34±0.01**	-	-	-	-	-	3.9	0.4	-	-	1.06
γ-Terpinene	**8.3±0.05**	16.18	0.76	-	3.19	0.76	4.3	9.2	34.4	5.7	8.47
Terpinolene	**0.29±0.01**	0.5	0.25	-	-	-	-	0.2	-	-	0.50
**Oxygenated monoterpenes**											
1,8-Cineole	**0.99±0.02**	-	-	-	-	-	-	-	-	-	0.01
cis-Sabinene hydrate	**0.33±0.01**	-	-	-	-	-	-	0.1	tr	-	0.39
Linalool	**0.61±0.03**	-	-	-	-	-	3.8	0.8	-	1.2	0.75
Borneol	**0.62±0.02**	-	-	9.15	-	-	0.9	0.4	-	0.5	2.61
Terpinen-4-ol	**1.08±0.02**	-	-	-	0.05	-	0.3	0.5	0.1	0.3	0.86
α-Terpineol	**0.27±0.01**	-	-	-	0.08	-	0.2	-	-	-	0.18
cis-β-Terpineol	**-**	-	-	-	-	-	-	-	-	-	-
trans-Carveol	**0.26±0.02**	-	-	-	-	-	-	-	-	-	-
p-Cumic aldehyde	**4.31±0.03**	-	-	-	-	-	-	-	-	-	-
Thymol	**41.06±0.3**	0.07	1.77	32.82	89.06	1.77	39.8	0.4	-	-	29.86
Carvacrol	**13.02±0.1**	24.28	58.56	32.98	0.09	48.56	5.7	68.3	14.2	76.5	34.56
Thymol methyl ether	**-**	-	-	-	-	-	-	-	-	-	0.26
Carvacrol methyl ether	**2.32±0.03**	-	-	-	-	-	-	-	1.9	-	0.08
Carvacryl acetate	**-**	-	-	1.8	0.22	-	-	tr	1.5	1.3	-
**Sesquiterpenes**											
β-Caryophyllene	**5.1±0.04**	8.59	7.41	6.15	0.68	7.41	1.1	1.6	-	2.8	1.00
α-Humulene	**0.48±0.02**	1.82	5.20	0.3	0.40	5.20	-	0.1	-	-	-
Aromadendrene	**0.45±0.02**	0.78	-	0.43	-	-	-	0.2	-	-	-
Viridiflorene	**0.7±0.02**	1.63	6.57	-	-	6.57	-	tr	0.2	-	-
Spathulenol	**0.61±0.02**	1.03	-	1.2	-	-	-	tr	-	-	-
Caryophyllene oxide	**4.1±0.03**	10.43	6.26	3.45	0.10	6.26	0.2	0.2	2.2	0.5	0.12
α-Bisabolol	**-**	-	-	0.64	-	-	-	-	-	-	-
α-Curcumene	**0.46±0.02**	-	-	-	-	-	-	-	-	-	-
α-Zingiberene	**0.39±0.02**	-	-	-	-	-	-	-	-	-	-
β-Sesquiphellandrene	**0.51±0.02**	-	-	-	-	-	-	-	-	-	-
Carotol	**0.34±0.01**	-	-	-	-	-	-	-	-	-	-
ar-Turmerone	**2.15±0.02**	-	-	-	-	-	-	-	-	-	-
ar-Dihydroturmerone	**0.75±0.02**	-	-	-	-	-	-	-	-	-	-
Cedr-8-en-13-ol	**0.33±0.01**	-	-	-	-	-	-	-	-	-	-
Curlone	**0.71±0.01**	-	-	-	-	-	-	-	-	-	-
**Others**											
Eugenol	**-**	-	-	-	-	-	-	tr	0.8	-	-
Durenol (2,3,5,6-tetramethylphenol)	**-**	5.54	2.18	-	-	12.18	-	-	-	-	-

Table S8 (continue)

Compound	Libya (our study)	Tunisia Monastir [S11]	Tunisia Bizerte [S8]	Tunisia Sousse [S8]	Tunisia Mograne [S13]	Spain [S21]	Turkey Izmir [S23]	Turkey Balikesir [S24]	Turkey Mugla [S24]	Turkey Canakkale [S24]	Egypt Mediterranean coast [S6]
**Monoterpene hydrocarbons**											
α-Thujene	**0.32±0.01**	0.2	0.01	0.02	1.33	0.1	1.3	-	-	-	-
α-Pinene	**0.38±0.01**	0.4	0.16	0.92	0.91	1.5	1.0	1.71	1.17	0.42	-
Camphene	**0.31±0.02**	0.2	0.09	0.47	tr	-	0.4	0.28	0.23	0.13	-
β-Pinene	**0.3±0.01**	0.3	0.01	2.50	2.01	0.3	-	0.14	0.13	0.06	-
β-Myrcene	**0.39±0.01**	0.5	0.59	2.03	tr	2.0	3.0	2.01	1.62	1.20	-
α-Phellandrene	**0.36±0.01**	-	-	-	1.55	0.2	0.4	tr	tr	tr	-
α-Terpinene	**1.08±0.02**	0.6	3.83	2.25	-	1.5	3.2	1.51	1.55	0.82	-
p-Cymene	**5.21±0.04**	5.3	0.13	1.25	13.17	13.2	21.0	7.71	10.48	6.10	2.97
o-Cymene	**-**	-	-	-	-	-	-	-	-	-	-
Limonene	**0.34±0.01**	0.8	-	-	0.43	0.4	0.5	0.31	0.30	0.26	-
γ-Terpinene	**8.3±0.05**	1.3	0.10	0.03	7.81	8.7	12.3	5.65	6.83	2.37	2.13
Terpinolene	**0.29±0.01**	0.1	0.09	1.30	-	0.2	tr	0.14	0.14	0.1 4	-
**Oxygenated monoterpenes**											
1,8-Cineole	**0.99±0.02**	-	-	-	-	0.4	-	-	-	-	-
cis-Sabinene hydrate	**0.33±0.01**	-	-	-	-	-	tr	0.15	0.10	0.18	-
Linalool	**0.61±0.03**	1.4	-	-	1.29	-	0.5	0.94	0.34	0.97	3.7
Borneol	**0.62±0.02**	1.6	0.01	0.10	1.22	0.3	-	0.90	0.84	1.01	-
Terpinen-4-ol	**1.08±0.02**	1.1	0.01	tr	tr	0.6	0.5	2.30	2.22	2.42	0.8
α-Terpineol	**0.27±0.01**	0.2	0.03	0.11	-	0.1	tr	0.04	0.03	0.04	-
cis-β-Terpineol	**-**	-	-	-	-	-	-	-	-	-	-
trans-Carveol	**0.26±0.02**	-	-	-	-	-	-	-	-	-	-
p-Cumic aldehyde	**4.31±0.03**	-	-	-	-	-	-	-	-	-	-
Thymol	**41.06±0.3**	0.3	81.49	69.95	0.26	6.4	18.6	0.29	0.30	0.33	62.9
Carvacrol	**13.02±0.1**	82.4	0.13	2.56	58.66	59.3	35.6	71.16	68.72	77.93	-
Thymol methyl ether	**-**	-	-	-	-	-	-	-	-	-	-
Carvacrol methyl ether	**2.32±0.03**	-	-	-	0.10	-	-	0.05	0.02	0.37	-
Carvacryl acetate	**-**	-	-	-	-	-	-	-	-	-	-
**Sesquiterpenes**											
β-Caryophyllene	**5.1±0.04**	1.1	-	-	0.29	2.2	0.2	0.70	0.67	0.79	-
α-Humulene	**0.48±0.02**	-	-	-	tr	-	-	0.09	0.09	0.12	-
Aromadendrene	**0.45±0.02**	-	-	-	-	-	-	-	-	-	-
Viridiflorene	**0.7±0.02**	-	-	-	-	-	-	-	-	-	-
Spathulenol	**0.61±0.02**	-	-	-	-	-	tr	-	-	-	-
Caryophyllene oxide	**4.1±0.03**	0.8	0.01	tr	0.07	-	tr	0.45	0.61	0.81	-
α-Bisabolol	**-**	-	-	-	-	-	-	-	-	-	-
α-Curcumene	**0.46±0.02**	-	-	-	-	-	-	-	-	-	-
α-Zingiberene	**0.39±0.02**	-	-	-	-	-	-	-	-	-	-
β-Sesquiphellandrene	**0.51±0.02**	-	-	-	-	-	-	-	-	-	-
Carotol	**0.34±0.01**	-	-	-	-	-	-	-	-	-	-
ar-Turmerone	**2.15±0.02**	-	-	-	-	-	-	-	-	-	-
ar-Dihydroturmerone	**0.75±0.02**	-	-	-	-	-	-	-	-	-	-
Cedr-8-en-13-ol	**0.33±0.01**	-	-	-	-	-	-	-	-	-	-
Curlone	**0.71±0.01**	-	-	-	-	-	-	-	-	-	-
**Others**											
Eugenol	**-**	0.1	-	-	-	-	-	0.03	0.03	0.10	-
Durenol (2,3,5,6-tetramethylphenol)	**-**	-	-	-	-	-	-	-	-	-	0.38

Table S8b. Chemical composition (%) of ***Thymus vulgaris*** essential oils from different countries and plant parts

Compound	Libya Wadi Al Khabta (our study)	Tunisia Zaghouan [S25]	Tunisia Sfax [S26]	Iran Mashhad [S48]	Iran Shiraz [S49]	Oman [S46]	Algeria Annaba [S28]	Saudi Arabia Riyadh [S47]	Bosnia and Herzegovina [S39]	Poland [S40]	Kashmir [S51]	Serbia Pomoravje [S38]
**Monoterpene hydrocarbons**												
α-Thujene	**0.42±0.01**	-	1.61	1.7±0.7	0.24	-	0.60	-	1.00	0.28	0.86±0.19	0.71
α-Pinene	**0.61±0.02**	-	1.74	1.2±0.4	4.54	2.6	0.60	0.18	1.50	0.27	0.59±0.14	1.75
Camphene	**0.47±0.01**	-	1.60	1.1±0.3	0.27	-	-	0.13	1.00	0.28	0.53±0.13	0.25
β-Pinene	**0.31±0.01**	-	0.29	-	0.90	0.9	0.10	-	0.70	0.14	-	-
β-Myrcene	**0.65±0.01**	0.43±0.01	2.36	1.2±1.2	1.04	-	1.39	0.15	1.50	1.14	1.23±0.03	4.09
α-Phellandrene	**-**	0.43±0.00	0.34	-	-	-	0.20	-	-	0.20	0.26±0.13	tr
α-Terpinene	**1.01±0.02**	4.37±0.03	-	1.4±0.9	1.27	-	2.09	-	1.90	1.02	1.25±0.12	2.65
p-Cymene	**2.57±0.03**	-	0.09	21.1±5.4	13.25	8.6	-	2.77	30.00	12.12	8.55±0.84	1.09
o-Cymene	**-**	-	16.55	-	-	-	20.32	0.39	-	-	-	-
Limonene	**-**	-	1.61	0.5±0.0	0.42	-	0.28	-	0.70	0.51	0.09±0.03	2.85
γ-Terpinene	**3.5±0.03**	2.08±0.02	7.80	6.6±0.0	5.09	5.2	18.75	1.18	9.00	10.20	9.48±1.84	4.58
Terpinolene	**0.2±0.01**	-	0.10	-	-	-	-	-	-	0.39	3.14±0.59	0.97
**Oxygenated monoterpenes**												
1,8-Cineole	**1.39±0.02**	-	-	0.4±0.4	0.44	-	-	-	1.40	0.88	0.69±0.16	0.30
cis-Sabinene hydrate	**-**	-	-	0.9±2.0	-	-	-	-	-	2.19	0.85±0.25	30.77
Linalool	**1.73±0.02**	-	4.41	2.4±0.1	2.14	0.6	-	0.38	2.40	2.44	0.10±0.05	7.89
Camphor	**-**	-	1.15	0.4±0.0	-	-	-	-	0.70	0.57	0.13±0.05	0.10
Borneol	**7.02±0.04**	-	2.02	2.1±0.4	-	-	-	0.90	1.70	1.56	1.26±0.33	0.28
Terpinen-4-ol	**2.01±0.03**	-	0.92	0.6±0.3	0.42	0.8	0.31	0.31	0.90	0.64	0.23±0.09	9.50
α-Terpineol	**0.47±0.01**	-	0.10	-	-	0.7	0.30	0.28	0.40	0.08	-	2.69
p-Cumic aldehyde	**2.80±0.02**	-	-	-	-	-	-	-	-	-	-	-
Carvone	**0.23±0.01**	-	-	-	-	-	-	-	-	-	-	-
Thymol	**20.91±0.2**	-	47.44	43.8±4.0	28.92	15.0	27.28	38.71	36.70	41.34	60.55±2.27	tr
Carvacrol	**41.38±0.3**	89.24±0.03	-	5.7±1.0	26.18	-	21.81	0.34	3.60	15.97	3.36±0.76	-
Thymol methyl ether	**-**	-	0.43	0.8±0.0	-	0.8	0.24	-	0.30	1.00	0.65±0.15	-
Carvacrol methyl ether	**-**	-	-	0.9±0.7	-	-	-	-	0.40	0.63	0.40±0.13	-
Thymol acetate	**-**	1.02±0.01	-	-	1.27	0.9	-	-	-	-	-	-
Carvacryl acetate	**-**	-	-	-	1.18	-	-	-	-	-	-	-
Geraniol	**-**	-	-	-	-	-	-	-	-	-	-	0.24
Geranyl acetate	**-**	-	-	-	-	-	-	-	-	-	-	0.48
Linalyl acetate	**-**	-	-	-	-	-	-	-	-	-	-	3.40
**Sesquiterpenes**												
β-Caryophyllene	**7.15±0.03**	1.83±0.01	2.09	3.6±0.2	2.49	2.1	0.86	0.91	0.40	3.64	1.75±0.24	2.03
α-Humulene	**0.42±0.01**	-	0.03	-	-	-	-	0.22	-	0.13	0.15±0.09	0.06
Aromadendrene	**0.24±0.01**	-	-	-	0.43	-	-	-	-	-	0.09±0.03	-
Viridiflorene	**0.32±0.01**	-	-	-	-	0.3	-	-	-	-	-	-
Spathulenol	**0.35±0.01**	-	-	-	1.18	0.5	-	-	-	-	0.13±0.05	-
Caryophyllene oxide	**1.63±0.01**	-	-	1.4±0.0	0.73	0.5	-	0.51	-	0.43	-	0.10
Germacrene D	**-**	-	-	-	-	-	-	-	-	-	0.14±0.05	0.58
δ-Cadinene	**0.32±0.01**	-	-	-	-	-	-	-	-	0.31	0.24±0.12	-
α-Zingiberene	**0.24±0.01**	-	3.40	-	-	-	-	-	-	-	-	-
Cedr-8-en-13-ol	**0.34±0.01**	-	-	-	-	-	-	-	-	-	-	-
**Others**												
Eugenol	**0.26±0.01**	-	-	-	-	0.9	-	-	-	-	0.05±0.03	-
Durenol (2,3,5,6-tetramethylphenol)	**-**	-	-	-	-	56.2	-	-	-	-	-	-

Table S8 (continue)

Compound	Libya Wadi Al Khabta (our study)	Serbia Jablanicki [S38]	France Richerenches [S38]	France Nyons [S38]	Romania [S41]	Italy Francesca [S37]	Italy Frigento [S37]	Italy Salerno [S37]	Turkey Erzincan [S44]	Czech Republic [S42]	Slovakia [S43]	Turkey Diyarbakır [S45]
**Monoterpene hydrocarbons**												
α-Thujene	**0.42±0.01**	tr	0.43	tr	0.89	-	-	-	-	0.26	0.5	0.5±0.07
α-Pinene	**0.61±0.02**	0.21	1.32	0.47	0.68	-	-	-	0.29	0.54	2.1	0.4±0.01
Camphene	**0.47±0.01**	0.38	1.19	1.17	0.69	-	-	-	tr	0.87	0.7	0.4±0.02
β-Pinene	**0.31±0.01**	-	-	-	0.31	-	tr	-	0.69	-	0.9	-
β-Myrcene	**0.65±0.01**	0.44	1.59	0.09	-	-	-	-	0.53	0.68	1.0	0.7±0.16
α-Phellandrene	**-**	-	0.11	-	-	-	-	-	tr	-	0.1	-
α-Terpinene	**1.01±0.02**	tr	1.30	-	-	-	0.1	-	0.58	-	0.8	1.0±0.23
p-Cymene	**2.57±0.03**	0.18	20.07	0.09	26.4	tr	tr	-	2.87	20.40	11.7	11.2±1.89
o-Cymene	**-**	-	0.06	-	-	-	-	-	-	-	-	-
Limonene	**-**	0.39	0.39	0.05	0.33	-	-	-	0.31	-	1.3	0.4±0.11
γ-Terpinene	**3.5±0.03**	0.09	9.03	-	3.6	tr	tr	tr	4.61	5.55	6.1	3.4a±0.37
Terpinolene	**0.2±0.01**	-	0.07	-	0.05	-	-	-	-	-	0.3	-
**Oxygenated monoterpenes**												
1,8-Cineole	**1.39±0.02**	0.31	0.72	0.37	-	tr	tr	-	tr	3.74	6.7	-
cis-Sabinene hydrate	**-**	0.31	0.17	-	2.3	0.5	0.2	0.1	0.22	-	-	-
Linalool	**1.73±0.02**	7.15	5.00	76.15	-	2.7	1.8	0.5	-	2.82	4.4	1.7a±0.07
Camphor	**-**	0.11	1.42	1.79	0.04	-	-	-	0.18	4.21	1.3	-
Borneol	**7.02±0.04**	1.00	1.50	0.40	1.85	0.2	0.5	-	2.14	3.78	2.2	-
Terpinen-4-ol	**2.01±0.03**	0.17	1.25	0.06	0.17	0.4	0.4	tr	0.14	-	1.9	1.1±0.30
α-Terpineol	**0.47±0.01**	0.09	0.16	0.11	0.1	-	-	-	0.44	-	0.5	0.1±0.01
p-Cumic aldehyde	**2.80±0.02**	-	-	-	-	-	-	-	-	-	-	-
Carvone	**0.23±0.01**	tr	0.09	-	-	-	-	-	0.17	-	-	-
Thymol	**20.91±0.2**	0.42	47.06	-	15.52	67.5	52.4	63.0	1.47	50.65	48.1	55.3±1.2
Carvacrol	**41.38±0.3**	-	3.24	0.05	1.85	5.7	7.1	6.1	72.47	-	5.5	8.7±3.03
Thymol methyl ether	**-**	-	0.19	-	10.78	-	-	-	-	-	-	-
Carvacrol methyl ether	**-**	-	0.32	-	6.26	-	tr	-	-	-	0.3	-
Thymol acetate	**-**	-	-	-	0.05	0.1	-	0.1	-	-	-	-
Carvacryl acetate	**-**	-	-	-	-	tr	0.1	-	-	-	-	-
Geraniol	**-**	59.75	0.05	-	-	0.2	0.7	0.2	-	-	tr	-
Geranyl acetate	**-**	16.72	-	0.06	-	-	0.1	-	-	-	-	-
Linalyl acetate	**-**	-	-	14.26	-	-	-	-	-	-	-	-
**Sesquiterpenes**												
β-Caryophyllene	**7.15±0.03**	3.67	1.79	2.27	3.14	0.8	0.6	0.2	4.21	2.13	2.3	4.2±0.18
α-Humulene	**0.42±0.01**	0.12	0.05	0.06	0.13	-	-	-	-	-	-	0.1±0.01
Aromadendrene	**0.24±0.01**	-	tr	-	4.4	-	-	-	-	-	-	0.3±0.01
Viridiflorene	**0.32±0.01**	-	tr	-	-	-	-	-	-	-	-	0.2±0.01
Spathulenol	**0.35±0.01**	tr	-	-	0.29	0.1	0.2	0.1	-	-	-	0.7±0.06
Caryophyllene oxide	**1.63±0.01**	0.50	0.13	0.41	-	1.7	6.5	2.2	0.12	1.71	0.3	0.9±0.08
Germacrene D	**-**	0.05	-	0.26	1.03	-	-	-	-	0.12	-	-
δ-Cadinene	**0.32±0.01**	-	0.16	tr	-	0.4	0.4	0.4	-	-	0.1	0.2±0.01
α-Zingiberene	**0.24±0.01**	-	-	-	-	-	-	-	-	-	-	-
Cedr-8-en-13-ol	**0.34±0.01**	-	-	-	-	-	-	-	-	-	-	-
**Others**												
Eugenol	**0.26±0.01**	-	0.08	-	-	tr	tr	tr	-	-	-	-
Durenol (2,3,5,6-tetramethylphenol)	**-**	-	-	-	-	-	-	-	-	-	-	-

Table S8 (continue)

Compound	Libya Wadi Al Khabta (our study)	Iran Gonbad [S50]	Morocco Taza [S29]	Morocco Taounate [S30]	Morocco Tiznit [S31]	Algeria Zemmoura [S27]	India [S52]	Greece Epirus [S35]	Spain València [S36]	Egypt Cairo (Aljabeili) [S33]	Egypt Shorouk City [S32]	Egypt Cairo (Saied) [S34]
**Monoterpene hydrocarbons**												
α-Thujene	**0.42±0.01**	0.33	-	0.73±0.08	0.97	-	-	-	0.3	-	-	-
α-Pinene	**0.61±0.02**	1.20	-	0.72±0.07	0.99	0.17	0.68	0.99	4.9	-	-	-
Camphene	**0.47±0.01**	0.78	-	-	-	0.03	-	0.21	17.0	-	-	-
β-Pinene	**0.31±0.01**	0.25	-	-	-	-	-	-	1.2	-	-	-
β-Myrcene	**0.65±0.01**	1.25	-	1.57±0.18	2.35	0.25	-	0.98	1.2	0.04	-	3.35
α-Phellandrene	**-**	0.10	-	-	0.34	-	-	0.11	tr	-	-	-
α-Terpinene	**1.01±0.02**	1.18	-	2.81±0.35	-	-	-	0.66	0.2	9.22	-	-
p-Cymene	**2.57±0.03**	30.90	-	10.56±1.30	9.12	1.89	-	5.00	1.1	10.50	2.27	13.50
o-Cymene	**-**	-	7.96	-	-	-	-	-	-	-	-	-
Limonene	**-**	-	-	-	0.93	0.09	0.97	0.08	1.9	-	-	-
γ-Terpinene	**3.5±0.03**	2.10	11.11	24.13±2.89	19.17	6.46	-	2.03	0.5	12.06	1.06	17.50
Terpinolene	**0.2±0.01**	0.14	-	-	-	-	-	-	0.1	0.25	0.49	-
**Oxygenated monoterpenes**												
1,8-Cineole	**1.39±0.02**	0.40	-	-	-	-	-	-	3.4	14.26	1.53	24.30
cis-Sabinene hydrate	**-**	-	-	-	-	-	-	-	0.5	-	-	-
Linalool	**1.73±0.02**	2.68	1.41	1.36±0.27	1.98	0.69	-	1.42	0.3	2.80	2.5	4.59
Camphor	**-**	0.53	-	-	-	-	-	-	46.4	-	0.84	-
Borneol	**7.02±0.04**	1.66	-	-	-	-	-	1.81	11.5	-	2.25	-
Terpinen-4-ol	**2.01±0.03**	0.96	-	0.64±0.09	0.91	-	-	0.72	2.3	0.65	0.85	2.20
α-Terpineol	**0.47±0.01**	0.13	-	-	0.39	-	-	-	0.5	1.10	0.23	-
p-Cumic aldehyde	**2.80±0.02**	-	-	-	-	-	-	-	-	-	-	-
Carvone	**0.23±0.01**	0.59	-	-	-	-	-	-	0.1	-	-	-
Thymol	**20.91±0.2**	36.81	16.50	17.35±2.65	21.51	89.58	40.14	0.98	-	41.04	78.21	48.00
Carvacrol	**41.38±0.3**	3.16	59.70	37.63±8.42	27.31	-	-	80.68	-	2.77	3.69	11.00
Thymol methyl ether	**-**	1.70	-	-	-	-	-	-	-	-	0.34	-
Carvacrol methyl ether	**-**	0.89	-	-	-	-	-	-	-	-	-	6.15
Thymol acetate	**-**	0.12	-	-	-	-	-	-	-	-	-	0.45
Carvacryl acetate	**-**	-	-	-	-	-	-	-	-	-	-	-
Geraniol	**-**	-	-	-	-	-	-	-	-	-	-	-
Geranyl acetate	**-**	-	-	-	-	-	-	-	-	-	-	-
Linalyl acetate	**-**	-	-	-	-	-	-	-	-	-	-	-
**Sesquiterpenes**												
β-Caryophyllene	**7.15±0.03**	2.80	1.44	2.51±0.57	2.40	0.08	-	2.27	-	0.43	1.62	2.45
α-Humulene	**0.42±0.01**	0.21	-	-	-	-	-	-	-	-	-	-
Aromadendrene	**0.24±0.01**	-	-	-	-	-	-	-	-	-	-	-
Viridiflorene	**0.32±0.01**	-	-	-	-	-	-	-	-	-	-	-
Spathulenol	**0.35±0.01**	-	-	-	-	-	-	-	1.6	-	-	-
Caryophyllene oxide	**1.63±0.01**	0.83	1.88	-	0.24	-	-	0.23	0.2	-	1.6	-
Germacrene D	**-**	0.24	-	-	-	-	-	-	0.3	-	-	-
δ-Cadinene	**0.32±0.01**	0.52	-	-	-	-	-	-	-	-	-	-
α-Zingiberene	**0.24±0.01**	-	-	-	-	-	-	-	-	-	-	-
Cedr-8-en-13-ol	**0.34±0.01**	-	-	-	-	-	-	-	-	-	-	-
**Others**												
Eugenol	**0.26±0.01**	-	-	-	-	-	-	-	-	-	-	-
Durenol (2,3,5,6-tetramethylphenol)	**-**	-	-	-	-	-	-	-	-	-	-	-

Notes:

- Values are expressed as percentage (%) of total essential oil composition; ± values are standard deviations as reported.
- "tr" = trace; "-" = not detected/not reported; "<0.05" and similar values are given as reported.
- Numbers in square brackets in the column headers refer to the reference list at the end of this file ([S1]–[S52]). The "our study" column (bold, highlighted) is repeated in every panel for comparison.
- Compounds are listed under standardized names; synonyms reported in the original studies were merged (e.g., eucalyptol = 1,8-cineole; trans-/(E)-caryophyllene = β-caryophyllene; cymol and ρ-cymene = p-cymene; methyl thymol = thymol methyl ether; methyl carvacrol and isothymol methyl ether = carvacrol methyl ether; ledene = viridiflorene; endo-borneol = borneol; thymyl acetate = thymol acetate; carvacryl acetate = carvacrol acetate).

- Compounds shown as rows are those reported in our study, the major constituents (≥5%) of each study, and the main compounds common to the studies; other minor constituents of the original profiles are not shown.

Table S9. Essential-oil composition and chemotype of ***Thymus capitatus* and *T. vulgaris*** from this study compared with literature reports from the Mediterranean region and neighbouring areas. **Main constituents are the three most abundant compounds in each source**. The row for this study is shaded. References for the literature sources are listed at the end of this file (S1–S52) and are cited as [S1]–[S52]. The retained oils are summarised in Figure 7 of the main text. The complete constituent matrix used for the PCA and clustering is given in Table S10.

ID	Origin	Month	Plant part	Main constituents (%)	Chemotype	Reference
A. *Thymus capitatus*						
C1	Libya – Al Dhahr Al Ahmar, eastern (this study)	March	Aerial parts	Thymol 41.1 · Carvacrol 13.0 · γ-Terpinene 8.3	Thymol	This study
C2	Libya – Sidi Al-Hamry (east)	Mar–Jun	Aerial parts	Carvacrol 24.3 · γ-Terpinene 16.2 · Caryophyllene oxide 10.4	Carvacrol	[S1]
C3	Libya – Abu-draa (east)	Mar–Jun	Aerial parts	Carvacrol 58.6 · β-Caryophyllene 7.4 · Ledene/viridiflorene 6.6	Carvacrol	[S1]
C4	Libya – Al Jabal Al Akhdar	Mar–Jun	Aerial parts	Carvacrol 48.6 · Phenol,2,3,5,6 tertramethyl 12.2 · β-Caryophyllene 7.4	Carvacrol	[S2]
C5	Libya – Zintan (NW)	May	Aerial parts	Carvacrol 68.1 · Thymol 12.3 · p-Cymene 3.2	Carvacrol	[S3]
C6	Libya – Msallata (NW)	December	Aerial parts	Carvacrol 56.3 · p-Cymene 9.5 · γ-Terpinene 8.4	Carvacrol	[S4]
C7	Egypt – north coast	April	Aerial parts	Carvacrol 33.0 · Thymol 32.8 · Borneol 9.2	Carvacrol/thymol	[S5]
C8†	Egypt – Mediterranean coast	Apr–Jun	–	Thymol 62.9 · 15,15-bi-1,4,7,10,13 Pentaoxacyclohexadecane 7.4 · 2,5-Octadecadiynoic acid, methyl ester 5.4	Thymol	[S6]
C9	Tunisia – Matmata	April	Leaves	Thymol 89.1 · p-Cymene 5.0 · γ-Terpinene 3.2	Thymol	[S7]
C10	Tunisia – Bizerte	March	Leaves	Thymol 81.5 · α-Terpinene 3.8 · α-Cubebene 3.4	Thymol	[S8]
C11	Tunisia – Sousse	March	Leaves	Thymol 70.0 · 2-Phenylethanol 3.2 · (E)-β-Ocimene 3.1	Thymol	[S8]
C12	Tunisia – Kairouan	Feb–Mar	Aerial parts/leaves	Carvacrol 65.4 · γ-Terpinene 6.8 · p-Cymene 6.2	Carvacrol	[S9]
C13	Tunisia – Zaghouan	January	leaves	Carvacrol 76.5 · γ-Terpinene 5.7 · p-Cymene 5.3	Carvacrol	[S10]
C14	Tunisia – Monastir	November	Aerial parts	Carvacrol 82.4 · p-Cymene 5.3 · Borneol 1.6	Carvacrol	[S11]
C15	Tunisia – Jendouba/Haouaria/Aïn Tounine	Jan; Jul–Aug	Aerial parts	Carvacrol 68.3 · p-Cymene 9.2 · γ-Terpinene 9.2	Carvacrol	[S12]
C16	Tunisia – Mograne	–	Leaves	Carvacrol 58.7 · p-Cymene 13.2 · γ-Terpinene 7.8	Carvacrol	[S13]
C17	Algeria	February	Leaves	Thymol 51.2 · Carvacrol 12.6 · γ-Terpinene 10.3	Thymol	[S14]
C18	Morocco	May	Leaves	Carvacrol 79.2 · o-Cymene 5.1 · α-Terpinolene 4.3	Carvacrol	[S15]
C19	Palestine – Ramallah	March	Aerial parts	Carvacrol 31.2 · γ-Terpinene 30.9 · o-Cymene 16.8	Carvacrol	[S16]
C20	Palestine – Hebron	March	Aerial parts	Thymol 40.4 · β-Caryophyllene 13.2 · Carvacrol methyl ether 10.7	Thymol	[S16]
C21†	Palestine – Jenin	March	Aerial parts	γ-Terpinene 67.0 · cis-B-Terpineol 12.9 · Carvacrol 6.4	p-Cymene/γ-terpinene	[S16]
C22	Cyprus	–	–	Carvacrol 34.6 · Thymol 29.9 · p-Cymene 9.9	Carvacrol/thymol	[S17]
C23	Greece – island	–	Aerial parts	Thymol 39.8 · p-Cymene 31.0 · Carvacrol 5.7	Thymol	[S18]
C24	Italy – Lascari (Sicily)	May	Aerial parts	γ-Terpinene 34.4 · p-Cymene 22.8 · Carvacrol 14.2	p-Cymene/γ-terpinene	[S19]

ID	Origin	Month	Plant part	Main constituents (%)	Chemotype	Reference
C25	Italy	–	Aerial parts	Carvacrol 79.9 · p-Cymene 4.4 · γ-Terpinene 3.7	Carvacrol	[S20]
C26	Spain	–	–	Carvacrol 59.3 · p-Cymene 13.2 · γ-Terpinene 8.7	Carvacrol	[S21]
C27†	Portugal	May	–	p-Cymene 4.8 · Myrcene 1.1 · α-Terpinene 0.9	p-Cymene/γ-terpinene	[S22]
C28	Turkey – İzmir	July	–	Carvacrol 35.6 · p-Cymene 21.0 · Thymol 18.6	Carvacrol	[S23]
C29	Turkey – Balıkesir	August	Aerial parts	Carvacrol 71.2 · p-Cymene 7.7 · γ-Terpinene 5.7	Carvacrol	[S24]
C30	Turkey – Muğla	August	Aerial parts	Carvacrol 68.7 · p-Cymene 10.5 · γ-Terpinene 6.8	Carvacrol	[S24]
C31	Turkey – Çanakkale	July	Aerial parts	Carvacrol 77.9 · p-Cymene 6.1 · Terpinen-4-ol 2.4	Carvacrol	[S24]
B. *Thymus vulgaris*						
V1	Libya – Wadi Al Khabta (this study)	March	Aerial parts	Carvacrol 41.4 · Thymol 20.9 · β-Caryophyllene 7.2	Carvacrol	This study
V2	Tunisia – Zaghouan	March	Leaves	Carvacrol 89.2 · α-Terpinene 4.4 · γ-Terpinene 2.1	Carvacrol	[S25]
V3	Tunisia – Sfax	April	Aerial parts	Thymol 47.4 · O-Cymene 16.6 · γ-Terpinene 7.8	Thymol	[S26]
V4	Algeria – Zemmoura	May	Aerial parts	Thymol 89.6 · γ-Terpinene 6.5 · p-Cymene 1.9	Thymol	[S27]
V5†	Algeria – Annaba	April	Leaves	Thymol 27.3 · Phenol,2-methyl-5-(1-methylethyl 21.8 · Benzene,1-methyl-2-(1-methylethyl) 20.3	Thymol	[S28]
V6	Morocco – Taza	May	Aerial parts	Carvacrol 59.7 · Thymol 16.5 · γ-Terpinene 11.1	Carvacrol	[S29]
V7	Morocco – Taounate	May	–	Carvacrol 37.6 · γ-Terpinene 24.1 · Thymol 17.4	Carvacrol	[S30]
V8	Morocco – Tiznit	Mar–Apr	–	Carvacrol 27.3 · Thymol 21.5 · γ-Terpinene 19.2	Carvacrol	[S31]
V9	Egypt – Shorouk City, Cairo	January	Leaves	Thymol 78.2 · Carvacrol 3.7 · Linalool 2.5	Thymol	[S32]
V10‡	Egypt – commercial oil (Cairo)	–	–	Thymol 41.0 · 1,8-Cineole 14.3 · γ-Terpinene 12.1	Thymol	[S33]
V11‡	Egypt – commercial oil (Cairo)	–	–	Thymol 48.0 · 1,8-Cineole 24.3 · γ-Terpinene 17.5	Thymol	[S34]
V12	Greece – Epirus	–	Leaves and flowers	Carvacrol 80.7 · p-Cymene 5.0 · β-Caryophyllene 2.3	Carvacrol	[S35]
V13	Spain – València	August	–	Camphor 46.4 · Camphene 17.0 · Borneol 11.5	Camphor	[S36]
V14	Italy – Francesca	–	Aerial parts	Thymol 67.5 · Carvacrol 5.7 · Linalool 2.7	Thymol	[S37]
V15	Italy – Frigento	–	Aerial parts	Thymol 52.4 · Carvacrol 7.1 · Caryophyllene oxide 6.5	Thymol	[S37]
V16	Italy – Salerno	–	Aerial parts	Thymol 63.0 · Carvacrol 6.1 · Citronellyl formate 2.5	Thymol	[S37]
V17	Serbia – Pomoravje	May	Aerial parts	Sabinene hydrate 30.8 · Terpinen-4-ol 9.5 · Linalool 7.9	Sabinene hydrate	[S38]
V18	Serbia – Jablanički	May	Aerial parts	Geraniol 59.8 · Geranylacetate 16.7 · Linalool 7.2	Geraniol	[S38]
V19	France – Richerenches	May	Aerial parts	Thymol 47.1 · p-Cymene 20.1 · γ-Terpinene 9.0	Thymol	[S38]
V20	France – Nyons	May	Aerial parts	Linalool 76.2 · Linalylacetate 14.3 · β-Caryophyllene 2.3	Linalool	[S38]
V21	Bosnia & Herzegovina	June	Aerial parts	Thymol 36.7 · p-Cymene 30.0 · γ-Terpinene 9.0	Thymol	[S39]
V22	Poland	June	Aerial parts	Thymol 41.3 · Carvacrol 16.0 · p-Cymene 12.1	Thymol	[S40]
V23	Romania	–	Aerial parts	p-Cymene 26.4 · Thymol 15.5 · Thymolmethyl ether 10.8	p-Cymene/γ-terpinene	[S41]
V24	Czech Republic	–	Aerial parts	Thymol 50.6 · p-Cymene 20.4 · γ-Terpinene 5.5	Thymol	[S42]
V25	Slovakia	–	Dried stalk	Thymol 48.1 · p-Cymene 11.7 · 1,8-Cineole 6.7	Thymol	[S43]
V26	Turkey – Erzincan	–	–	Carvacrol 72.5 · γ-Terpinene 4.6 · β-Caryophyllene 4.2	Carvacrol	[S44]
V27	Turkey – Diyarbakır	July	–	Thymol 55.3 · p-Cymene 11.2 · Carvacrol 8.7	Thymol	[S45]

ID	Origin	Month	Plant part	Main constituents (%)	Chemotype	Reference
V28†	Oman – Jabal Al Abyadh	April	Aerial parts	Durenol 56.2 · Thymol 15.0 · p-Cymene 8.6	Other	[S46]
V29†	Saudi Arabia – Riyadh	December	Leaves	o -Thymol 38.7 · Furan, Furan, tetra hydro-3-methyl 12.2 · Thymol 3.8	Other	[S47]
V30	Iran – Mashhad	July	Aerial parts	Thymol 43.8 · p-Cymene 21.1 · γ-Terpinene 6.6	Thymol	[S48]
V31	Iran – Shiraz	August	leaves	Thymol 28.9 · Carvacrol 26.2 · p-Cymene 13.2	Carvacrol/thymol	[S49]
V32	Iran – Gonbad	Flowering	Aerial parts	Thymol 36.8 · p-Cymene 30.9 · Carvacrol 3.2	Thymol	[S50]
V33	India – Kashmir (Ganga Choti)	Flowering	–	Thymol 60.5 · γ-Terpinene 9.5 · p-Cymene 8.6	Thymol	[S51]
V34†	India – Zamin Uthukuli	–	Leaves	Thymol 40.1 · 5-Allyl-4-[1-(p-aminophenyl)ethylidenehydrazono]-6-methyl-2-phenylpyrimidin 14.8 · Methylene-1,3a,4,5,6,6a-hexahydropentalen-1-ol 12.7	Thymol	[S52]

† Excluded from PCA and clustering because of incomplete or ambiguous compound. ‡ Commercial oil of unknown origin, excluded from PCA and clustering.

Chemotype = dominant constituent; carvacrol/thymol = both phenols ≥25% and within 1.5-fold.

Table S10. Complete constituent matrix (% of total oil) for the *Thymus capitatus* (Panel A, 8 variables) and *T. vulgaris* (Panel B, 12 variables) datasets compiled in Table S8; IDs, origins and references are those of Table S9. Values were transcribed from the original reports (this study: means). Rows for this study are shaded yellow; datasets excluded from PCA and hierarchical clustering (†, ‡; Table S9) are shaded grey and marked "No" in the "In PCA" column. The 28 retained rows per panel are the input matrix of Figure 7.

Panel A. *Thymus capitatus* (31 datasets; 28 used in PCA).

ID	Origin	In PCA	Thymol	Carvacrol	p-Cymene	γ-Terpinene	Linalool	Borneol	β-Caryophyllene	Caryophyllene oxide
**C1**	Libya – Al Dhahr Al Ahmar, eastern (this study)	Yes	41.06	13.02	5.21	8.3	0.61	0.62	5.1	4.1
C2	Libya – Sidi Al-Hamry (east)	Yes	0.07	24.28	3.71	16.18	–	–	8.59	10.43
C3	Libya – Abu-draa (east)	Yes	1.77	58.56	–	0.76	–	–	7.41	6.26
C4	Libya – Al Jabal Al Akhdar	Yes	1.77	48.56	–	0.76	–	–	7.41	6.26
C5	Libya – Zintan (NW)	Yes	12.27	68.07	3.24	3.09	1.21	1.58	1.01	0.51
C6	Libya – Msallata (NW)	Yes	6.54	56.27	9.51	8.39	3.43	1.27	3.57	–
C7	Egypt – north coast	Yes	32.82	32.98	–	–	–	9.15	6.15	3.45
C8†	Egypt – Mediterranean coast	No	62.9	–	2.97	2.13	3.7	–	–	–
C9	Tunisia – Matmata	Yes	89.06	0.09	5.04	3.19	–	–	0.68	0.10
C10	Tunisia – Bizerte	Yes	81.49	0.13	0.13	0.10	–	0.01	–	0.01
C11	Tunisia – Sousse	Yes	69.95	2.56	1.25	0.03	–	0.10	–	tr
C12	Tunisia – Kairouan	Yes	1.35	65.38	6.25	6.75	1.51	–	4.94	–
C13	Tunisia – Zaghouan	Yes	–	76.5	5.3	5.7	1.2	0.5	2.8	0.5
C14	Tunisia – Monastir	Yes	0.3	82.4	5.3	1.3	1.4	1.6	1.1	0.8
C15	Tunisia – Jendouba/Haouaria/Aïn Tounine	Yes	0.4	68.3	9.2	9.2	0.8	0.4	1.6	0.2
C16	Tunisia – Mograne	Yes	0.26	58.66	13.17	7.81	1.29	1.22	0.29	0.07
C17	Algeria	Yes	51.22	12.59	–	10.30	2.29	–	2.01	1.21
C18	Morocco	Yes	–	79.22	–	–	–	–	4.31	0.81
C19	Palestine – Ramallah	Yes	3	31.25	–	30.94	6.19	–	4.61	0.77
C20	Palestine – Hebron	Yes	40.35	0.58	8.41	3.61	–	–	13.23	1.80
C21†	Palestine – Jenin	No	5.51	6.44	1.41	67	–	–	1.65	0.11
C22	Cyprus	Yes	29.86	34.56	9.94	8.47	0.75	2.61	1.00	0.12
C23	Greece – island	Yes	39.8	5.7	31.0	4.3	3.8	0.9	1.1	0.2
C24	Italy – Lascari (Sicily)	Yes	–	14.2	22.8	34.4	–	–	0.6	2.2
C25	Italy	Yes	0.3	79.9	4.4	3.7	0.9	0.4	2.3	0.2
C26	Spain	Yes	6.4	59.3	13.2	8.7	1.9	0.3	2.2	0.2
C27†	Portugal	No	–	–	4.8	–	–	–	–	–
C28	Turkey – İzmir	Yes	18.6	35.6	21.0	12.3	0.5	–	0.2	tr
C29	Turkey – Balıkesir	Yes	0.29	71.16	7.71	5.65	0.94	0.90	0.70	0.45
C30	Turkey – Muğla	Yes	0.30	68.72	10.48	6.83	0.34	0.84	0.67	0.61
C31	Turkey – Çanakkale	Yes	0.33	77.93	6.10	2.37	0.97	1.01	0.79	0.81

Panel B. *Thymus vulgaris* (34 datasets; 28 used in PCA).

ID	Origin	In PCA	Thymol	Carvacrol	p-Cymene	γ-Terpinene	Linalool	Borneol	β-Caryophyllene	Caryophyllene oxide	Geraniol	Sabinene hydrate	Camphor	1,8-Cineole
**V1**	Libya – Wadi Al Khabta (this study)	Yes	20.91	41.38	2.57	3.5	1.73	7.02	7.15	1.63	–	–	–	1.39
V2	Tunisia – Zaghouan	Yes	–	89.24	–	2.08	–	–	1.83	–	–	–	–	–
V3	Tunisia – Sfax	Yes	47.44	–	0.09	7.80	4.41	2.02	2.09	–	–	–	1.15	–
V4	Algeria – Zemmoura	Yes	89.58	–	1.89	6.46	0.69	–	0.08	–	–	–	–	–
V5†	Algeria – Annaba	No	27.28	–	–	–	–	–	0.86	–	–	–	–	–
V6	Morocco – Taza	Yes	16.50	59.70	–	11.11	1.41	–	1.44	1.88	–	–	–	–
V7	Morocco – Taounate	Yes	17.35	37.63	10.56	24.13	1.36	–	2.51	–	–	–	–	–
V8	Morocco – Tiznit	Yes	21.51	27.31	9.12	19.17	1.98	–	2.40	0.24	–	–	–	–
V9	Egypt – Shorouk City, Cairo	Yes	78.21	3.69	2.27	1.06	2.5	–	1.62	1.6	–	–	–	1.53
V10‡	Egypt – commercial oil (Cairo)	No	41.04	2.77	10.50	12.06	2.80	–	0.43	–	–	–	–	14.26
V11‡	Egypt – commercial oil (Cairo)	No	48.00	11.00	13.50	17.50	4.59	–	2.45	–	–	–	–	24.30
V12	Greece – Epirus	Yes	0.98	80.68	5.00	2.03	1.42	1.81	2.27	0.23	–	–	–	–
V13	Spain – València	Yes	–	–	1.1	–	0.3	11.5	–	0.2	–	0.5	46.4	3.4
V14	Italy – Francesca	Yes	67.5	5.7	tr	tr	2.7	0.2	0.8	1.7	0.2	0.5	–	tr
V15	Italy – Frigento	Yes	52.4	7.1	tr	tr	1.8	0.5	0.6	6.5	0.7	0.2	–	tr
V16	Italy – Salerno	Yes	63.0	6.1	–	tr	0.5	–	0.2	2.2	0.2	0.1	–	–
V17	Serbia – Pomoravje	Yes	tr	–	1.09	4.58	7.89	0.28	2.03	0.10	0.24	35.75	0.10	0.30
V18	Serbia – Jablanički	Yes	0.42	–	0.18	0.09	7.15	1.00	3.67	0.50	59.75	0.31	0.11	0.31
V19	France – Richerenches	Yes	47.06	3.24	20.07	9.03	5.00	1.50	1.79	0.13	0.05	0.17	1.42	0.72
V20	France – Nyons	Yes	–	0.05	0.09	–	76.15	0.40	2.27	0.41	–	–	1.79	0.37
V21	Bosnia & Herzegovina	Yes	36.70	3.60	30.00	9.00	2.40	1.70	0.40	–	–	–	0.70	1.40
V22	Poland	Yes	41.34	15.97	12.12	10.20	2.44	1.56	3.64	0.43	–	2.19	0.57	0.88
V23	Romania	Yes	15.52	1.85	26.4	3.6	–	1.85	3.14	1.4	–	2.74	0.04	–
V24	Czech Republic	Yes	50.65	–	20.40	5.55	2.82	3.78	2.13	1.71	–	–	4.21	3.74
V25	Slovakia	Yes	48.1	5.5	11.7	6.1	4.4	2.2	2.3	0.3	tr	–	1.3	6.7
V26	Turkey – Erzincan	Yes	1.47	72.47	2.87	4.61	–	2.14	4.21	0.12	–	0.22	0.18	tr
V27	Turkey – Diyarbakır	Yes	55.3	8.7	11.2	3.4	1.7	–	4.2	0.9	–	–	–	–
V28†	Oman – Jabal Al Abyadh	No	15.0	–	8.6	–	0.6	–	–	0.5	–	–	–	–
V29†	Saudi Arabia – Riyadh	No	3.82	–	2.77	1.18	0.38	0.90	0.91	0.51	–	–	–	–
V30	Iran – Mashhad	Yes	43.8	5.7	21.1	6.6	2.4	2.1	3.6	1.4	–	0.9	0.4	0.4
V31	Iran – Shiraz	Yes	28.92	26.18	13.25	5.09	2.14	–	2.49	0.73	–	–	–	0.44
V32	Iran – Gonbad	Yes	36.81	3.16	30.90	2.10	2.68	1.66	2.80	0.83	–	0.21	0.53	0.40
V33	India – Kashmir (Ganga Choti)	Yes	60.55	3.36	8.55	9.48	0.10	1.26	1.75	0.35	–	0.85	0.13	0.69
V34†	India – Zamin Uthukuli	No	40.14	–	–	–	–	–	–	–	–	–	–	–

– = not reported or not detected; tr = trace (t, tr). Both were entered as 0% in the PCA, which used square-root-transformed, mean-centred values (Section 4 of the main text). Geraniol, sabinene hydrate, camphor and 1,8-cineole were used for *T. vulgaris* only.

References

S1. El-Jalel, L.F.; et al. Difference in chemical composition and antimicrobial activity of *Thymus capitatus* L. essential oil at different altitudes. *Future J. Pharm. Sci.* **2018**, *4*, 156–160.

S2. El-jalel, L.F.; et al. Evaluation of the antimicrobial effect of *Thymus capitatus* essential oil (EO) extract on bacteria isolated from urinary tract infection. *JAJBSR* **2021**, *3*, 39–46.

S3. Džamić, A.M.; et al. Libyan *Thymus capitatus* essential oil: antioxidant, antimicrobial, cytotoxic and colon pathogen adhesion-inhibition properties. *J. Appl. Microbiol.* **2015**, *119*, 389–399.

S4. Suwaydan, A.; Altraiki, M.J. GC-MS analysis of essential oil components of the Libyan *Thymus capitatus*. *Lebda Med. J.* **2025**, *10*, 1–11.

S5. Salama, M.M.; Taher, E.E.; El-Bahy, M.M. Molluscicidal and mosquitocidal activities of the essential oils of *Thymus capitatus* Hoff. et Link. and *Marrubium vulgare* L. *Rev. Inst. Med. Trop. Sao Paulo* **2012**, *54*, 281–286.

S6. Mohyeldin, M.M.; et al. Comparative anti-herpes simplex virus type 1 and chemical profiling of *Thymus capitatus* and *Artemisia herba-alba* collected from North Africa. *Nat. Prod. Res.* **2023**, *37*, 4038–4041.

S7. Mkaddem, M.G.; et al. Essential oil of *Thymus capitatus* Hoff. et Link. from Matmata, Tunisia: gas chromatography-mass spectrometry analysis and antimicrobial and antioxidant activities. *J. Med. Food* **2010**, *13*, 1500–1504.

S8. Chedia, A.; et al. Comparison of chemical composition, antioxidant and antimicrobial activities of *Thymus capitatus* L. essential oils from two Tunisian localities (Sousse and Bizerte). *Int. J. Agron. Plant Prod.* **2013**.

S9. Zaïri, A.; et al. Chemical composition, fatty acids profile and biological properties of *Thymus capitatus* (L.) Hoffmanns, essential oil. *Sci. Rep.* **2019**, *9*, 20134.

S10. Megdiche-Ksouri, W.; et al. Potential use of wild *Thymus algeriensis* and *Thymus capitatus* as source of antioxidant and antimicrobial agents. *J. New Sci.* **2015**, *23*, 1046–1056.

S11. Hizem, A.; et al. Activity of *Thymus capitatus* essential oil components against in vitro cultured *Echinococcus multilocularis* metacestodes and germinal layer cells. *Parasitology* **2019**, *146*, 956–967.

S12. Bounatirou, S.; et al. Thermal stability of the essential oils isolated from Tunisian *Thymus capitatus* Hoff. et Link.: Effect on the chemical composition and the antioxidant and antibacterial activities. *Acta Aliment.* **2010**, *39*, 299–307.

S13. Hosni, K.; et al. Enzyme-assisted extraction of essential oils from thyme (*Thymus capitatus* L.) and rosemary (*Rosmarinus officinalis* L.): Impact on yield, chemical composition and antimicrobial activity. *Ind. Crops Prod.* **2013**, *47*, 291–299.

S14. Goudjil, M.B.; et al. Biological activities of essential oils extracted from *Thymus capitatus* (Lamiaceae). *S. Afr. J. Bot.* **2020**, *128*, 274–282.

S15. Benoutman, A.; et al. Phytochemical composition, antioxidant and antifungal activity of *Thymus capitatus*, a medicinal plant collected from northern Morocco. *Antibiotics* **2022**, *11*, 681.

S16. Barkat, A. Variations of the chemical components and biological activities of *Thymus capitatus* essential oil from three regions in Palestine. Thesis, An-Najah National University, Nablus, Palestine, 2022.

S17. Xylia, P.; et al. Quality characteristics and essential oil properties of *Thymus capitatus*, *Mentha piperita*, and *Sideritis cypria* dried under different conditions. *Plants* **2024**, *13*, 3150.

S18. Maniki, E.; et al. Chemical composition, antioxidant, and antibiofilm properties of essential oil from *Thymus capitatus* plants organically cultured on the Greek Island of Lemnos. *Molecules* **2023**, *28*, 1154.

S19. Casiglia, S.; et al. Influence of harvesting time on composition of the essential oil of *Thymus capitatus* (L.) Hoffmanns. & Link. growing wild in northern Sicily and its activity on microorganisms affecting historical art crafts. *Arab. J. Chem.* **2019**, *12*, 2704–2712.

S20. Gonçalves, J.C.R.; et al. Essential oil composition and antinociceptive activity of *Thymus capitatus*. *Pharm. Biol.* **2017**, *55*, 782–786.

S21. Grande-Tovar, C.D.; et al. Chitosan films incorporated with *Thymus capitatus* essential oil: mechanical properties and antimicrobial activity against degradative bacterial species isolated from tuna (*Thunnus sp.*) and swordfish (*Xiphias gladius*). *J. Food Sci. Technol.* **2018**, *55*, 4256–4265.

S22. Alves-Silva, J.M.; et al. Effect of *Thymbra capitata* (L.) Cav. on inflammation, senescence and cell migration. *Nutrients* **2023**, *15*, 1930.

S23. Goren, A.C.; et al. Analysis of essential oil of *Coridothymus capitatus* (L.) and its antibacterial and antifungal activity. *Z. Naturforsch. C J. Biosci.* **2003**, *58*, 687–690.

S24. Özek, T.; et al. Composition of the essential oil of *Coridothymus capitatus* (L.) Reichb. fil. from Turkey. *J. Essent. Oil Res.* **1995**, *7*, 309–312.

S25. Benmoussa, H.; et al. Effect of extraction methods on kinetic, chemical composition and antibacterial activities of Tunisian *Thymus vulgaris* L. essential oil. *Sep. Sci. Technol.* **2016**, *51*, 2145–2152.

S26. Akermi, S.; et al. In-depth study of *Thymus vulgaris* essential oil: Towards understanding the antibacterial target mechanism and toxicological and pharmacological aspects. *Biomed Res. Int.* **2022**, *2022*, 3368883.

S27. Medjadji, B.; Benahmed, F.; Kadi, I. GC-MS analysis of *Thymus vulgaris* L. and *Ptychotis verticillata* (Desf.) essential oils: multi-bioactive potential as natural therapeutic alternatives. *Farmacia* **2025**, *73*.

S28. Abid, F.; et al. GC/MS analysis of *Thymus vulgaris* essential oil and antibacterial effect towards multidrug-resistant *Staphylococcus aureus*. *J. Chem. Health Risks* **2025**.

S29. Chahboun, N.; et al. Chemical composition, biological activities, and anti-corrosion performance of Moroccan essential oil *Thymus vulgaris* from the Oued Amlil region, Taza. *Int. J. Electrochem. Sci.* **2024**, *19*, 100859.

S30. Belbachir, Y.; et al. Preliminary characterization of volatile compounds of *Thymus vulgaris* from Morocco by headspace solid-phase microextraction HS-SPME and GC-MS. *Preprints.org* **2023**.

S31. Labiad, M.; et al. Biological properties and chemical profiling of essential oils of *Thymus (vulgaris, algeriensis and broussonettii)* grown in Morocco. *Chem. Data Collect.* **2022**, *37*, 100797.

S32. AbdelRazek, M.M.; et al. Comparative chemical and biological study of essential oils and n-hexane extracts of *Thymus vulgaris* and *Thymus serpyllum* (Lamiaceae). *Sci. Rep.* **2026**.

S33. Aljabeili, H.S.; Barakat, H.; Abdel-Rahman, H.A. Chemical composition, antibacterial and antioxidant activities of thyme essential oil (*Thymus vulgaris*). *Food Nutr. Sci.* **2018**.

S34. Saied, A.M.; et al. Harnessing natural feed additives for sustainable production and economics: the role of *Thymus vulgaris* L. oil as an antimicrobial agent and a growth promoter in improving production and health of broiler chickens. *Front. Immunol.* **2025**, *16*, 1695478.

S35. Sidiropoulou, E.; et al. In vitro antioxidant, antimicrobial, anticoccidial, and anti-inflammatory study of essential oils of oregano, thyme, and sage from Epirus, Greece. *Life* **2022**, *12*, 1783.

S36. Llorens-Molina, J.A.; et al. Chemodiversity of wild populations of aromatic plants as source of valuable essential oil profiles. A study on *Thymus vulgaris* L. from Valencia (Spain). *Nat. Volatiles Essent. Oils* **2020**, *7*, 29–50.

S37. Mancini, E.; et al. Studies on chemical composition, antimicrobial and antioxidant activities of five *Thymus vulgaris* L. essential oils. *Molecules* **2015**, *20*, 12016–12028.

S38. Satyal, P.; et al. Essential oil characterization of *Thymus vulgaris* from various geographical locations. *Foods* **2016**, *5*, 70.

S39. Niksic, H.; et al. Cytotoxicity screening of *Thymus vulgaris* L. essential oil in brine shrimp nauplii and cancer cell lines. *Sci. Rep.* **2021**, *11*, 13178.

S40. Wesolowska, A.; Grzeszczuk, M.; Jadczak, D. Comparison of the chemical composition of essential oils isolated by water-steam distillation and hydrodistillation from garden thyme (*Thymus vulgaris* L.). *J. Essent. Oil Bear. Plants* **2016**, *19*, 832–842.

S41. Popa, C.L.; et al. Chemical and biochemical characterization of essential oils and their corresponding hydrolats from six species of the Lamiaceae family. *Plants* **2021**, *10*, 2489.

S42. Gupta, N.; et al. Synthetic polyploidization induces enhanced phytochemical profile and biological activities in *Thymus vulgaris* L. essential oil. *Sci. Rep.* **2024**, *14*, 5608.

S43. Galovičová, L.; et al. *Thymus vulgaris* essential oil and its biological activity. *Plants* **2021**, *10*, 1959.

S44. Aksit, H.; et al. Chemical composition and antifungal activities of the essential oils of Thymus species (*Thymus pectinatus*, *Thymus convolutus*, *Thymus vulgaris*) against plant pathogens. *J. Essent. Oil Bear. Plants* **2022**, *25*, 200–207.

S45. Gedikoğlu, A.; et al. Evaluation of *Thymus vulgaris* and *Thymbra spicata* essential oils and plant extracts for chemical composition, antioxidant, and antimicrobial properties. *Food Sci. Nutr.* **2019**, *7*, 1704–1714.

S46. Mohammed, Z.; et al. Composition of essential oil of *Thymus vulgaris* grown in Oman. *J. Essent. Oil Bear. Plants* **2016**, *19*, 475–478.

S47. Al-Asmari, A.K.; et al. Chemical composition of essential oil of *Thymus vulgaris* collected from Saudi Arabian market. *Asian Pac. J. Trop. Biomed.* **2017**, *7*, 147–150.

S48. Soleimani, M.; et al. Analysis of the volatile compounds in *Thymus vulgaris* L. using improved HS-SPME-GC-MS and comparison with conventional methods. *J. Essent. Oil Bear. Plants* **2014**, *17*, 1233–1240.

S49. Khalili, G.; et al. Solvent-free microwave extraction of essential oils from *Thymus vulgaris* L. and *Melissa officinalis* L. *Ind. Crops Prod.* **2018**, *119*, 214–217.

S50. Moghaddam, M.; Mehdizadeh, L. Chemical composition and antifungal activity of essential oil of *Thymus vulgaris* grown in Iran against some plant pathogenic fungi. *J. Essent. Oil Bear. Plants* **2020**, *23*, 1072–1083.

S51. Ahmad Khan, A.; Amjad, M.S.; et al. GC-MS analysis and biological activities of *Thymus vulgaris* and *Mentha arvensis* essential oil. *Turk. J. Biochem.* **2019**, *44*, 388–396.

S52. Kathirvel, K.; et al. In vitro evaluation of antimicrobial, anti-inflammatory and cytotoxic property against HepG2 cell line of essential oil extracted from *Thymus vulgaris* leaves. *J. Microbiol. Biotechnol. Food Sci.* **2025**, *14*, e11404.
